# Whole genomes from Angola and Mozambique inform about the origins and dispersals of major African migrations

**DOI:** 10.1038/s41467-023-43717-x

**Published:** 2023-12-02

**Authors:** Sam Tallman, Maria das Dores Sungo, Sílvio Saranga, Sandra Beleza

**Affiliations:** 1 University of Leicester, Department of Genetics & Genome Biology, University Road, Leicester, LE1 7RH UK; 2 Universidade 11 de Novembro, Rua das Mangueiras, Cabinda, Cabinda Province Angola; 3 Universidade Pedagógica, Avenida Eduardo Mondlane, CP 2107 Maputo, Mozambique; 4 Present Address: Genomics England, 1 Canada Square, London, E14 5AB UK

**Keywords:** Genetic variation, Genetic variation

## Abstract

As the continent of origin for our species, Africa harbours the highest levels of diversity anywhere on Earth. However, many regions of Africa remain under-sampled genetically. Here we present 350 whole genomes from Angola and Mozambique belonging to ten Bantu ethnolinguistic groups, enabling the construction of a reference variation catalogue including 2.9 million novel SNPs. We investigate the emergence of Bantu speaker population structure, admixture involving migrations across sub-Saharan Africa and model the demographic histories of Angolan and Mozambican Bantu speakers. Our results bring together concordant views from genomics, archaeology, and linguistics to paint an updated view of the complexity of the Bantu Expansion. Moreover, we generate reference panels that better represents the diversity of African populations involved in the trans-Atlantic slave trade, improving imputation accuracy in African Americans and Brazilians. We anticipate that our collection of genomes will form the foundation for future African genomic healthcare initiatives.

## Abstract (web summary)

African human genome variation remains under-sampled. Here, the authors present a collection of 350 whole genome sequences from Angola and Mozambique and model the timing and extent of significant demographic events in African history.

## Introduction

With over 300 million speakers (5% of the global population) spanning a region of sub-Saharan Africa of 10 million km^2^, the Bantu languages represent one of the world’s largest language groups. This vast distribution has been largely attributed to the Bantu Expansion, a succession of dispersals originating in the inland Savannahs of Central-West Africa some 6000–5000 years before the present-day (BP)^1–3^, spanning the African Iron Age, and likely driven by the development of agriculture^4,5^ and periods of habitat change^6,7^^.^ Historical records (www.slavevoyages.org) show that Bantu speaking communities were also heavily affected by the forced movement of peoples to the Americas during the trans-Atlantic slave trade, contributing over half of all slaves to have disembarked across the New World. Despite this significant role in the histories of both Africa and the Americas, Bantu speaking communities remain under-represented in human genomics research.

Today, advances in next-generation sequencing technologies have begun to facilitate the curation of whole-genome sequencing (WGS) data representing the full spectrum of variation across diverse human populations^8,9^. Such endeavours are critical next steps towards understanding how genetic diversity is structured globally and providing reference variation catalogues for a broad range of medical genetics initiatives^10,11^. Although Bantu speaking communities have been involved in several WGS projects to date, such as the 1000 Genomes Project (1000G)^12^, African Genome Variation Project (AGVP)^13^, Ugandan Genome Resource^14^ and H3Africa-Baylor dataset (H3AB)^15^, gaps remain including a scarcity of data from populations on the edge of the Bantu Expansion such as those from Angola and Mozambique.

Prior analyses of autosomal SNP array^16,17^ and linguistic data^18^ from regions surrounding Angola and Mozambique have proved crucial in forming our understanding of major dispersal routes undertaken during the Bantu Expansion. This includes favouring the so-called late-split model concerning the diversification of Western and Eastern Bantu languages^19^. Furthermore, archaeological data^20,21^ has hinted at additional, more complex patterns including multi-step dispersals involving Bantu speaking communities across sub-Saharan Africa, suggesting our understanding of migrations into and out of Angola and Mozambique remains incomplete. Methods leveraging additional WGS data have the potential to shed further light on these events.

Moreover, as former Portuguese colonies, Angola and Mozambique are recorded as being the origin of over 5 million and 500,000 slaves to have crossed the Atlantic respectively from 1526–1875 (www.slavevoyages.org). Indeed, 96% of all slaves to arrive in south-east Brazil left from ports located in Angola and Mozambique, whilst 25% of all slaves to arrive in the USA originated from Angola in addition to those that disembarked from ports across much of coastal west Africa. With limited genomic data available from these important embarkation regions, current reference variation panels lack the complete diversity of African populations that have contributed ancestry to populations throughout the Americas^16,22^ potentially leading to asymmetries in our ability to impute variation for analysis using genome-wide association studies^23^.

To support the continued discovery and cataloguing of genomic variation in human populations and to further our understanding of the Bantu Expansion, we sequenced the genomes of 300 individuals from Cabinda, a northern exclave of Angola, and 50 individuals from Maputo, the capital of Mozambique. Utilising the power and flexibility of these WGS datasets, we discover rare variation, fine-scale population structure, and perform analyses using haplotype-based inference tools and our own model-based simulation framework to reconstruct complex dispersals of Bantu speaking populations across sub-Saharan Africa. Here, we show that the Bantu expansion conforms to a series of founder events starting from western Africa south of the equatorial rainforest, where Bantu communities differentiated into branches that either continued further south into Namibia, or east into the regions surrounding Zambia (likely associated with the proliferation of Eastern Bantu languages) and further into east and south Africa. We infer distinct periods and intensity of admixture during the Bantu dispersal. During the initial stages of the Bantu dispersals into Cabinda and Angola in the west, and into Mozambique in the east, admixture with local populations was limited; this was followed by more extensive admixture in later stages of the Bantu dispersals in south-west (in Namibia and south-western Botswana) and south-east (south-eastern Botswana and South Africa) Africa. Our results bring together concordant views from genomics, archaeology, and linguistics to paint an updated view of the complexity of the Bantu Expansion.

Moreover, we generate reference panels that better represents the diversity of African populations involved in the Atlantic slave trade, improving imputation accuracy in African Americans and Brazilians over the 1000 Genomes Project. Overall, this dataset represents a timely addition to the growing number of whole-genome sequences from Africa, provides insights into the history of Bantu speaking migrant communities, and takes another step towards ensuring the potential benefits of genomics extends to all parts of the globe.

## Results

### A novel collection of genomes from Cabinda, Angola and Maputo, Mozambique

Genomic DNA was extracted using saliva samples collected with informed consent and sequenced using the Illumina HiSeq X™ platform to an average autosomal read depth of ~12*X* from 300 individuals sampled in Cabinda and 50 individuals sampled in Maputo (Table 1) labelled according to ethnolinguistic groups^24^ derived from self-reported parental and grand-parental language (Supplementary Data 1). Among individuals collected in Cabinda (CAB), 79% reported as having a single familial language belonging to one of many closely related Kikongo (Kongo) dialects, the predominant language spoken in the region. Those collected from Maputo (MOZ) were more ethnolinguistically heterogeneous, with 62% of individuals reported as speaking Tswa-Ronga (Tsonga) or Chopi languages commonly found in the south of Mozambique and 28% speaking Makua dialects commonly found in the north. Place-of-birth largely mirrored expectations corresponding to language distributions (Supplementary Data 1).

**Table 1:** Summary of newly sequenced individuals and autosomal genomic variation in our CAB and MOZ datasets

Dataset	CAB	MOZ
Ethnolinguistic Group	Kongo (*n* = 238), Kimbundu (*n* = 14), Ovimbundu (*n* = 15), Ovambo (*n* = 2), Chokwe (*n* = 2), Other (*n* = 26)	Tsonga (*n* = 18), Chopi (*n* = 13), Makua (*n* = 14), Sena (*n* = 3), Shona (*n* = 1), Other (*n* = 1)
Total samples (post QC)	297	50
Mean coverage (X)	11.56 ± 1.51	12.07 ± 1.40
Mean reads mapped hg19/GRCh137 (%)	90.19 ± 6.83	93.94 ± 2.86
Total SNPs	27,116,464	17,064,063
Total INDELs <50 bp	2,964,806	1,840,852
Nonsynonymous	134,936	67,349
Synonymous	112,122	63,876
Downstream	273,729	163,500
Upstream	257,040	152,531
5’ UTR	80,086	44,898
3’ UTR	327,251	184,218
Intronic	11,548,432	6,774,720
Intergenic	14,662,426	8,828,426
Splicing	2509	1066
ncRNA	3,950,561	2,347,461

Other includes Luba (*n* = 1), Ngala (*n* = 1), Lunda (*n* = 1) and individuals with mixed parental and/or grand-parental language groups. ± Shows one standard deviation. Further details regarding sequencing statistics, linguistic affiliations and place-of-birth can be found in Supplementary Data 1.

After sample processing, variant calling, and quality-control we identified 33.1 million total variants among CAB and MOZ, including 29.9 million SNPs and 3.9 million short INDELs, with an average of 4.1 million SNPs per sampled genome (Table 1). Approximately 2.9 million SNPs were novel when compared to the dbSNP155 (https://ftp.ncbi.nih.gov/snp/), 91% of which were singletons. Modest differences in genetic diversity between CAB and MOZ were apparent, with CAB showing an increased coverage-adjusted average heterozygosity ratio compared to MOZ (CAB = 1.99, MOZ = 1.95, bootstrap *p* < 0.0001) (Supplementary Fig. 1).

Overall, 21% of autosomal SNPs identified in CAB or MOZ were not observed in the 1000G, AGVP, or H3AB. Of these 6.1 million total dataset-specific SNPs, ~72% were singletons and 95% were rare (minor allele frequency (MAF) < 0.05). Upon examining shared *f2* alleles^25^, we find the largest proportion of rare variants genotyped among CAB and MOZ are shared with previously sequenced Bantu speaking groups, with notable yet reduced sharing with African-derived populations from the Americas (Supplementary Table 1).

### Population structure in a pan African context

To investigate population structure and diversity of CAB and MOZ in a pan-African context, we merged genotypes called across newly sequenced individuals with a single familial language group, unrelated to the 4th degree (as estimated using KING^26^), and with <5% European ancestry (as estimated using ADMIXTURE^27^) (Supplementary Data 1) with sequenced African groups from the 1000G, AGVP, H3AB, the Simons Genome Diversity Project (SGDP)^28^, and three high-coverage ancient African genomes^29–31^ (Supplementary Data 2).

**Fig. 1:**
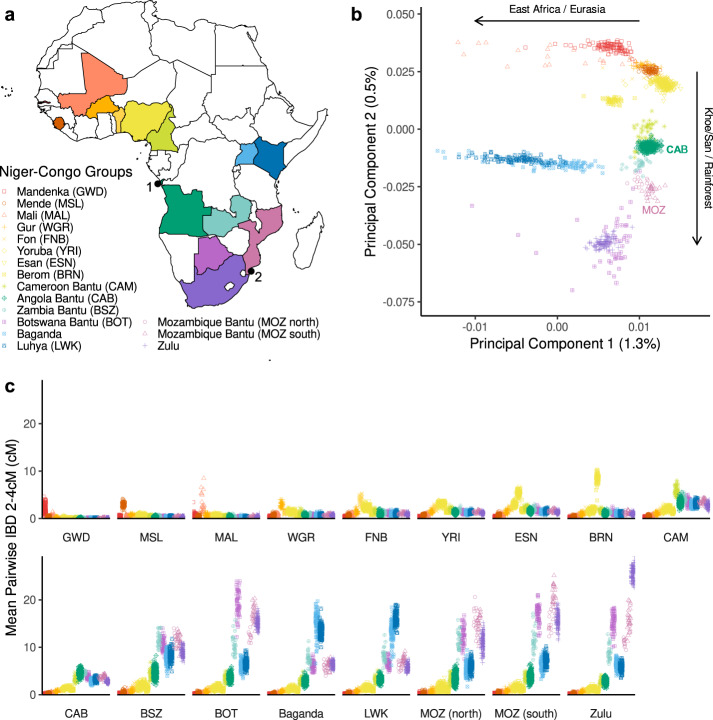
Population structure of CAB and MOZ in the context of sequenced Niger-Congo groups. **a** Map denoting colour and shape corresponding to each Niger-Congo group and their country-of-origin present in a merged dataset consisting of CAB and MAP, 1000G, AGVP, and H3AB, and modern and ancient African genomes (Supplementary Data 2). Collection sites in Cabinda (1) and Maputo (2) are also shown. We emphasise that sequenced groups do not reflect the complete genetic or ethnolinguistic diversity of their country-of-origin. Map made with Natural Earth. Free vector and raster map data @ naturalearthdata.com. **b** Top two principal components of PCA calculated on African groups in the merged dataset (Supplementary Data 2). PC1 and PC2 explain 1.2% and 0.5% of observed variation respectively. PCs here are zoomed to emphasise Niger-Congo population structure. Arrows denote approximate direction of additional super-groups present in the unzoomed PCA (see Supplementary Fig. 2b for unzoomed PCA). **c** Average cumulative length of IBD haplotypes between 2 and 4 cM that individuals share with another individual from each Niger-Congo population in the merged dataset. Bantu speakers from outside of Cameroon are ordered according to geographic distance from Cabinda (see also Supplementary Table 3). Mandenka from The Gambia; MSL, Mende from Sierra Leone; MAL, People from Mali; WGR, Gur from Burkina Faso; FNB Fon from Benin; YRI, Yoruba from Nigeria; ESN, Esan from Nigeria; BRN, Berom from Nigeria; CAM, Bantoid and Bantu speakers from Cameroon; CAB, Bantu speakers collected in Cabinda, Angola; BSZ, Bantu speakers from Zambia; Baganda from Uganda; LWK, Luhya from Kenya; MOZ (north), Makua or Lomwe peoples from Mozambique; MAP (south) Tsonga (Tswa-Ronga) or Chopi peoples from Mozambique; BOT, Bantu speakers from Botswana; Zulu from South Africa.

PCA^32^ performed using this merged WGS dataset captures population structure among Niger-Congo peoples largely reflecting regional admixture in addition to isolation by distance (Fig. 1b, Supplementary Fig. 2). PC2 separates Bantu speakers from other West African groups, with CAB and MOZ appearing closest to Bantu speakers from Cameroon (CAM) and Zambia (BSZ). As supported by *f4* statistics^33^ (Supplementary Fig. 3), such clustering is likely a result of the modest impact of admixture from non-Bantu speaking groups (e.g. Afro-Asiatic, Khoe/San) relative to Bantu speakers from eastern (Baganda, Luhya (LWK)) and southern Africa (Botswana (BOT), Zulu).

Within MOZ, population structure is apparent on a PCA largely separating the northern Makua (MOZ (north)) from the southern Tsonga and Chopi peoples (MOZ (south)). *f4* statistics suggest only limited difference in east African or Khoe/San related ancestries between these groups (*Z* > −2 and *Z* < 2) (Supplementary Fig. 3). However, we do observe significant statistics testing clade structure compared to the South African Zulu (*f4*(MOZ (south), MOZ (north); Zulu, Chimp), *Z* = 4.4) (Supplementary Table 2), with positive values of *f4* indicating elevated allele sharing with MOZ (south) and suggesting population structure within MOZ likely also reflects the genetic differentiation of north Mozambican from south Mozambican and South African Bantu speakers over time in addition to any subtle differences in east African or Khoe/San admixture components.

Examining Identical-By-Descent (IBD) haplotypes^34^ shared across sequenced Niger-Congo speaking groups in the dataset, we observe geographic stratification of recent ancestries. Longer, more recent haplotypes (>8 cM, approximately <250 years before present (BP)^35^) (Supplementary Fig. 4a) are shared almost exclusively within groups, consistent with more restricted population movements in recent centuries. For CAB, CAM and non-Bantu speaking west Africans, this is also true of intermediate haplotypes (4–8 cM, ~750 BP) (Supplementary Fig. 4b). However, individuals among MOZ still share a number of ancestors with Bantu speakers among the Zulu, BSZ and BOT in this period, illustrating their recent common histories. When focusing on shorter, more ancient haplotypes (2–4 cM, ~1500 BP), shared ancestry across all Bantu speaking groups is observed (Fig. 1c). However, geographic structure is still apparent, revealing an increasing gradient of pairwise IBD from west to east and south. Here, MOZ shares higher mean pairwise IBD with BSZ than with CAB (BSZ = 11.5 cM > CAB = 3.8 cM, permutation test *p* < 0.0001), supporting inference that Zambia was an intermediate location for Bantu speaker migrations into the region surrounding present-day Mozambique^15^.

Examining ancient IBD (2–4 cM) sharing within groups (Fig. 1c), clear differences between regions become apparent. MOZ (south) share higher mean pairwise IBD than MOZ (north) (MOZ (north) = 16.1 cM < MOZ (south) = 20.8 cM, permutation test *p* < 0.0001), corroborating the recent discovery of north to south serial founder events in the genetic history of the region^17^. This pattern is similarly observed when analysing short runs of homozygosity (ROH)^36^ (Supplementary Fig. 5). Extending these findings, among Bantu speakers from outside of CAM, we observe a strong correlation between within-group IBD sharing and geographic distance from Cabinda (Pearson’s *r* = 0.89, *p* = 0.003) (Supplementary Table 3), evidencing a progressive reduction in genetic diversity associated with the expansion of Eastern Bantu speakers into and across east and south-east Africa^1,15–17^. Conversely, no evidence of founder events reflecting a southward dispersal of Bantu speakers through the equatorial rainforests^6,15,17^ are apparent when examining differences in mean pairwise IBD sharing within CAM or CAB (CAM = 4.6 cM <CAB = 5.4 cM, permutation test *p* > 0.05).

To further explore the population structure of CAB and MOZ, we merged our extended WGS dataset with a selection of modern and ancient individuals genotyped at sites present on the Human Origins Array (HOA)^29,31,37–43^ (Supplementary Data 3) or a second dataset composed of individuals genotyped on various Illumina array panels, including previously genotyped Angolan and Mozambican Bantu speakers^13,16,17,44–49^ (Supplementary Data 4) and performed PCA and haplotype-based clustering using fineSTRUCTURE^50^. PCAs align with those performed on our WGS dataset (Fig. 1b). CAB and MOZ cluster closely across the top PCs (PC1 to PC4, Supplementary Fig. 6a, b and Supplementary Fig. 7a, b) that largely separate groups according to variation in local admixture components. PC5 (Illumina, Supplementary Fig. 6c) and PC6 (HOA, Supplementary Fig. 7c) instead appear to reflect Niger-Congo specific population structure resulting from isolation by distance. Here, CAB and MOZ cluster distinctly alongside neighbouring Western and South-Eastern Bantu-speaking groups respectively. Further supporting our inference using WGS data (Fig. 1b), fineSTRUCTURE infers substructure among MOZ separating Tsonga and Chopi from the Makua peoples, with newly sequenced individuals clustering alongside members of their respective ethnolinguistic groups previously collected across Mozambique (Supplementary Fig. 8b). Makua peoples also cluster closely with Malawian Bantu speakers in the HOA dataset (Supplementary Fig. 6, Supplementary Fig. 8a), aligned with the considerable geographic and cultural overlap between Malawi and northern Mozambique^24^. Within CAB, fineSTRUCTURE clusters broadly separate Kongo from Ovimbundu and Kimbundu peoples predominantly born in the central-western regions of Angola (Supplementary Data 1). Notably, however, whilst 85% of Kongo peoples among CAB clustered largely independently from any other ethnolinguistic group, 75% of Kongo peoples previously collected from the capital of Luanda appeared within ethnolinguistically heterogenous fineSTRUCTURE clusters (Supplementary Fig. 8b), signifying a complex relationship between language and genetics across the region. Together, these results highlight the recent appearance of present-day national borders relative to the emergence of genetic structure across sub-Saharan Africa.

### Hunter-gatherer related admixture

The genetic architecture of sub-Saharan Africa has been shaped by admixture involving Bantu speaking migrants and local populations. However, consistent with a history involving almost complete replacement of local genetic diversity across large parts of central-west and south-eastern Africa^15,17,42^, and supporting inference using PCA (Fig. 1b, Supplementary Fig. 6, Supplementary Fig. 7), *f4* statistics (Supplementary Fig. 3), and Y-chromosome and mitochondrial DNA lineages (Supplementary Fig. 17, Supplementary Note 3), SOURCEFIND^51^ analyses performed using our extended HOA dataset (Supplementary Fig. 9), suggests that CAB and MOZ are best represented as having 99% (minimum (min) = 90%, maximum (max) = 100%, sd ± 2%) and 97% (min = 93%, max = 97%, sd ± 2%) Bantu speaker related ancestry (represented by the Cameroonian Lemande) respectively. These results are broadly recaptured using ADMIXTURE^27^ clustering (Supplementary Fig. 10).

**Fig. 2:**
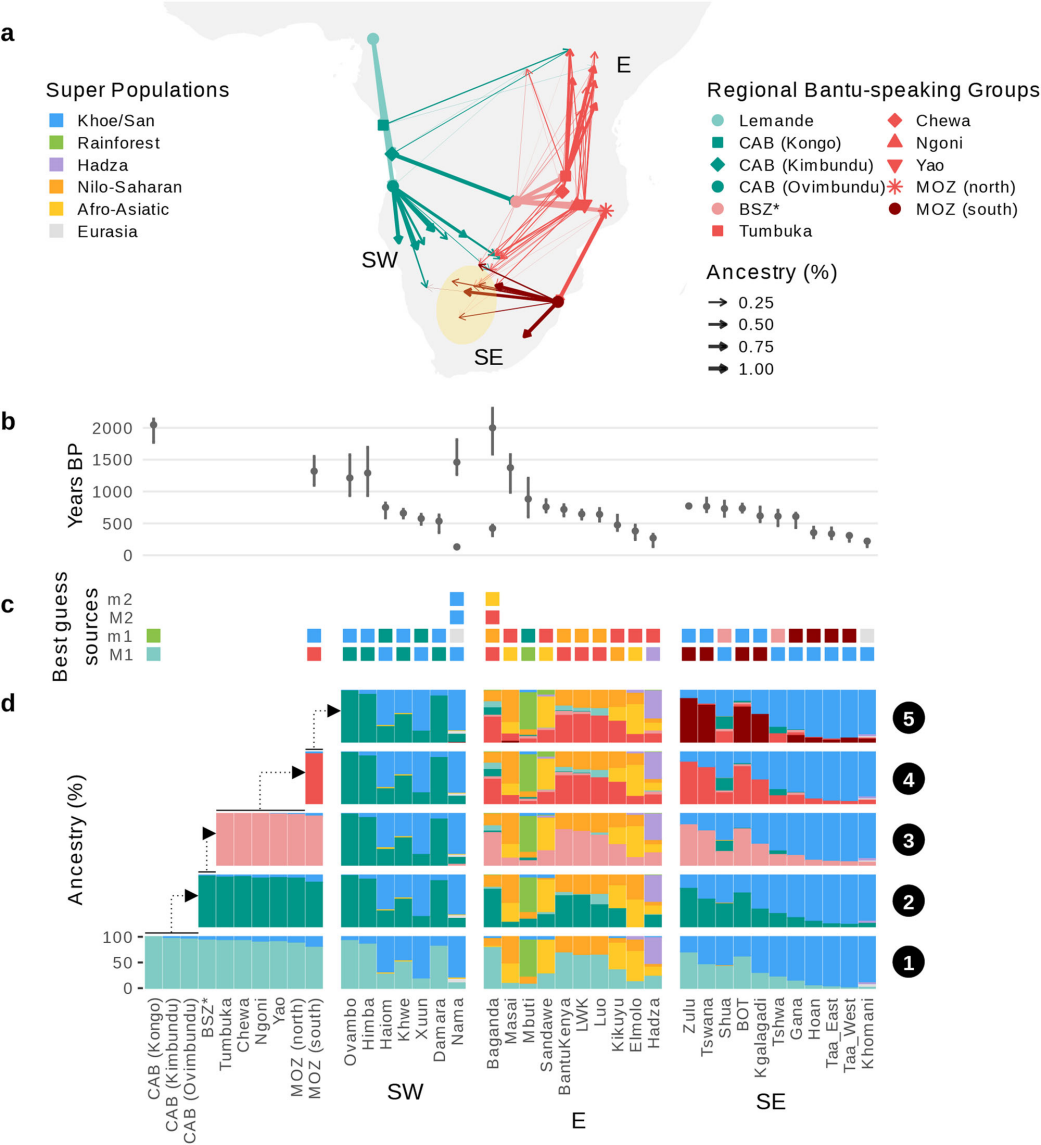
Bantu speaker dispersals into and out of Angola and Mozambique and admixture. **a** Map of sub-Saharan Africa with arrows connecting target groups in the HOA dataset to respective source population(s) (“Regional Bantu speaking Groups”) as inferred using SOURCEFIND under a stepwise approach. The origin, colour, and size of each arrow details the proportion of ancestry from the closest Bantu speaking source inferred to contribute some ancestry proportion to each target group in the final step, as shown in (**d**). Coordinates of source and target groups were inferred using the approximate region of Africa where that language is primarily spoken^24^, collection site, or place-of-birth information (Supplementary Data 1). BOT was excluded due to within-group heterogeneity (Supplementary Fig. 11). Three geographic regions of extensive admixture with local populations are labelled as South-West (SW), East (E), and South-East (SE). Shaded yellow area represents the Kalahari semi-desert. Arrows do not necessarily reflect direct migration but are instead indicative of relative patterns of shared ancestries. Map made with Natural Earth. Free vector and raster map data @ naturalearthdata.com. **b** Date(s) of admixture estimated using fastGLOBETROTTER. Generation time = 27 years per generation^54^. 95% confidence intervals were estimated using bootstrap resampling over 100 replicates (n’s are in Supplementary Data 1 and 3; date estimates are in Supplementary Data 5). **c** Best guess of major (M) and minor (m) admixing source populations for either event 1 (latest) or 2 (earliest) inferred using fastGLOBETROTTER. **d** Ancestry proportions in target groups estimated by SOURCEFIND using a five-step approach. Starting from step 1 where Cameroonians are the only Bantu speaking group included as potential source population, ancestry proportions in each target group were re-estimated after additional Bantu speaking groups were cumulatively added as potential source populations in each step (as shown by dotted arrows), whilst also being excluded as target groups in that and successive steps (Supplementary Note 5). Colours represent groups merged into “Super Populations or “Regional Bantu speaking Groups” as shown in (**a**). BSZ*, Bantu speakers from Zambia that cluster independently from Malawians as inferred using fineSTRUCTURE (Supplementary Note 5); other population acronyms are in Fig. 1.

Using fastGLOBETROTTER^52,53^ (Fig. 2b, c, Supplementary Data 5), we find the small 3–4% contribution from a Khoe/San-like source group (best represented by a 2000-year-old individual from Ballito Bay, South Africa^31^) in Tsonga and Chopi peoples among MOZ (south) are derived from single-date admixture events at ~1300 BP (27 years per generation^54^, 95% confidence interval (CI) 1050–1550 BP), aligned with dates and ancestry proportions observed in previously genotyped south Mozambican groups^17^ and Tsonga peoples from South Africa^55^. We also find evidence of admixture involving a small 1–5% western rainforest hunter-gatherer component (best represented by the Cameroonian Bakola) in the Kongo peoples among CAB estimated to have occurred ~2050 BP (CI 1800–2150 BP), identical to those previously estimated in this group^16^. Ovimbundu and Kimbundu peoples among CAB and Makua peoples among MOZ (north) are modelled by SOURCEFIND as having as little as <1% rainforest hunter-gatherer or Khoe/San related ancestry respectively.

### Complex dispersals of Bantu speakers into and out of Angola and Mozambique

Whilst contact with local populations appears to have had a modest impact on the genetic diversity of Bantu speakers among CAB and MOZ (Fig. 1b, Supplementary Figs. 9, 10), previous genetic research involving Bantu speaking groups from Angola and Mozambique have revealed that migrations out of both regions had a widespread impact on the genetic diversity of sub-Saharan Africa^16,17^. Importantly, the flexibility afforded by our WGS datasets now enables such models of migration and admixture developed in these earlier studies to be tested using largely independent datasets (such modern and ancient individuals genotyped on the HOA). To reconstruct dispersal into and out of the regions surrounding Angola and Mozambique over recent millennia, we therefore used SOURCEFIND and fastGLOBETROTTER to characterise ancestries and date admixture in all present-day African groups across our extended HOA dataset (Supplementary Data 3). Here, ancestry proportions were inferred using a stepwise approach, with a cumulatively increasing number of Bantu speaking groups (including those among CAB and MOZ) added as possible sources of ancestry in each step (Supplementary Note 5).

Replicating the findings first described in Patin et al.^16^ we observe first that haplotypes among 39 present-day groups across the dataset spanning much of east and south-east Africa match more closely to Bantu speakers in CAB relative to those from Cameroon  (Fig. 2d, step 1, 2). These results are consistent with a late-split model of the Bantu Expansion, where migration into eastern Africa occurred only after an initial southward movement through the equatorial rainforests^19^.

Following this initial spread, additional patterns of relative haplotype matching suggests subsequent branching dispersals likely occurred around central-west Africa, becoming apparent when Zambian Bantu speakers are added as an additional source of ancestry (Fig. 2, step 3). Here, Ovimbundu peoples among CAB remain as the closest source of Bantu speaker ancestries among groups from Namibia, consistent with a continued movement of Western Bantu speakers through southern Angola. This was likely followed by two distinct periods of admixture involving Bantu speakers and Khoe/San groups respectively, estimated to have occurred between 1300 BP (CI 1000–1700) and 550–750 BP (CI 350–800 BP) and aligned with previous estimates^40^. However, further supporting recent research suggesting populations moved into the region surrounding Zambia before migrating further south or east^15^, we find that Zambian Bantu speakers largely replace ancestries previous matched to CAB in groups from across east and south-east Africa. Future studies that include WGS data from Central-Africa countries such as the DRC may be able to refine the modelling of eastern Africa ancestry^56^.

Building on this model of Bantu speaker migration into east and south Africa presented in Choudhury et al.^15^, we observe that intermediate Zambian ancestries (Supplementary Note 5) are replaced by Bantu speakers from Malawi (Fig. 2, Supplementary Fig. 11b, step 4, 5) in groups from Uganda, Kenya, and Tanzania, consistent with a continued association of eastward moving populations through central Africa before further dispersals towards the north-eastern Great Lakes region. Alternatively, we find MOZ (north) (Fig. 2, step 4) and then MOZ (south) (Fig. 2, Supplementary Fig. 11c, step 5) largely replace Zambian ancestries among Bantu speakers from South Africa and southern Botswana, providing additional support for models described in Semo et al.^17^ in which Bantu speaking populations from southernmost parts of the continent first spread through north to south Mozambique. As widely reported^13,15,40,55^, we find Bantu speaking groups from South Africa and southern Botswana show evidence of Khoe/San admixture dated to ~600–750 BP (CI 500–900 BP), significantly later than those estimated in Tsonga and Chopi peoples among MOZ (south) (Fig. 2c).

Interestingly, the sampling distribution of present-day Bantu speaking groups whose admixing source most closely matches to MOZ (south-east (SE), Fig. 2a, d) relative to CAB (south-west (SW), Fig. 2a, d) are perfectly subdivided by the Kalahari semi-desert, revealing this feature as a potential barrier to the expansion of Bantu speaking communities in southern Africa. In apparent contrast to these regional patterns of shared ancestry with CAB and MOZ among modern groups, ancestry in a 1100-year-old individual from the south-eastern border of Botswana (Botswana_Taukome_1100BP, SE)^43^ was recently modelled using the Ovambo (who derive 98% of their ancestry from CAB, Fig. 2d). Performing analogous qpAdm tests^57,58^, however, we find that models including MOZ (south), MOZ (north), or BSZ similarly provide working fits (Supplementary Table 4). Moreover, ADMIXTURE clusters (Supplementary Fig. 10) and PCA (Supplementary Fig. 6c) suggest Bantu speaker ancestry in Botswana_Taukome_1100BP appear most similar to South-Eastern Bantu speaking groups (such as those among MOZ) whereas Bantu speaker ancestries observed in 1400-year-old individuals from the northern Okavango Delta (Botswana_Xaro_1400BP, SW) appear more similar to Western Bantu speaking groups (such as those among CAB), mirroring patterns observed among present-day groups from neighbouring regions.

### Demographic histories and split time estimates

Observations of recent shared ancestry between Eastern Bantu speaking populations and Western Bantu speakers from regions directly south-west of the equatorial rainforest (such as Cabinda) relative to those from closer to the Bantu heartland in Cameroon ^1,3^ (Fig. 2) are widely regarded as supporting evidence for a late-split model of the Bantu Expansion^19^. However, chronologies underlying such events, and their demographic consequences, remain topics of debate^18,59,60^. Largely free from the confounding effects of substantial admixture (Fig. 1b, Supplementary Fig. 9,10), CAB and MOZ are well-placed to interrogate demography specific to their Western and Eastern Bantu speaking ancestors utilising methods enabled by WGS.

To investigate the population size (*Ne*) and separation histories of CAB and MOZ, we used the non-parametric Multiple Sequentially Markovian Coalescent (MSMC2)^61,62^ and genome-wide genealogies estimated using Relate^63^. We also investigate the demographic and separation histories under an Approximate Bayesian Computation (ABC) framework^64^, simulating 135,000 whole-chromosomes^65^ (chromosome 1) using realistic recombination rates and error rates typical of WGS data^34^ under a clean-split model (Supplementary Fig. 12), often considered a lower bound for estimating genetic splits. This simulated data was then compared to CAB and MOZ across a set of summary statistics (Supplementary Table 5), selected based on their ability to inform on various demographic parameters (Supplementary Table 6, Supplementary Fig. 13). This includes IBD and ROH haplotype-based statistics, known to improve evaluations of recent demography^66,67^.

**Fig. 3:**
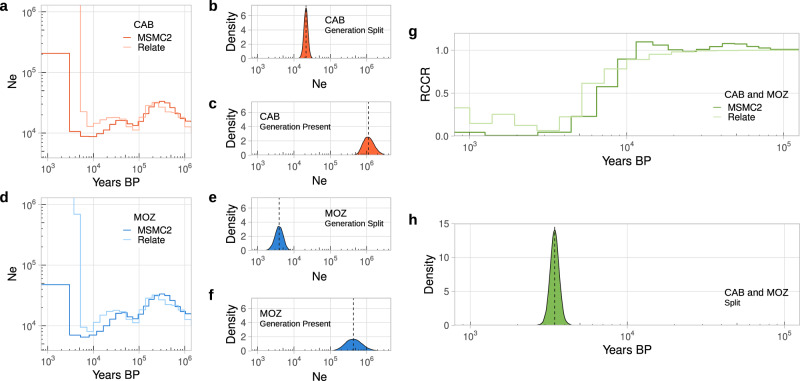
Demographic models of population size and separation histories inferred using whole genome sequencing data from CAB and MOZ. **a** Effective population size history of CAB using within-population coalescence rates estimated using Relate with 40 genomes and MSMC2 with four high-coverage (37X) genomes. **b** ABC posterior distribution for parameter denoting CAB effective population size at the generation in which CAB and MOZ separated (Generation Split). **c** ABC posterior distribution for parameter denoting CAB effective population size in most recent generation (Generation Present). **d**, **e**, **f** As in (**a**, **b**, **c**) but for MOZ. Relate was run independently for MOZ (north) and MOZ (south). **g** Separation history of CAB and MOZ estimated using Relative Cross-Coalescence Rates (RCCR) estimated using Relate with 40 genomes sampled from CAB, 27 from MOZ (south) or 13 from MOZ (north) or MSMC2 using two high-coverage (37X) genomes from either CAB or MOZ (Supplementary Note 3). Separation times were taken as the first generation going backwards-in-time in which RCCR is greater than or equal to 0.5 (**h**) ABC posterior distributions for parameters denoting the generation during which CAB and MOZ split (Generation Split) using 40 chromosomes (chromosome 1) randomly sampled from CAB and MOZ. Dashed lines represent the medians of the posterior distributions. Full ABC posterior estimates can be found in Supplementary Table 7. Generation time = 27 years per generation^54^. Population acronyms are defined in Fig. 1.

Concerning population size histories, concordant patterns from all three methodologies are observed. Here, MOZ is estimated as having a lower ancestral *Ne* than CAB (Fig. 3a, b, c, e), perhaps due to founder events associated with the dispersal of Bantu speaking communities into and within east and south-east Africa (Fig.1c, Supplementary Table 3). This was followed by rapid growth in both populations (Fig. 3a, b, d, f), likely driven by a transition to more sedentary lifestyles^68^.

Using Relative cross-coalescence rates (RCCR), the split between CAB and MOZ is estimated to have occurred at ~5900 BP with MSMC2 and 4,800 BP with Relate (generation where RCCR > 0.5, 27 years per generation^54^; Fig. 3g). These split times are more recent than those estimated between CAB or MOZ and non-Bantu speaking West African groups such as the Yoruba (YRI) (Supplementary Fig. 14). Calculating posterior parameter estimates using ABC under a clean-split model (Supplementary Figs. 12, 13) places the CAB and MOZ split somewhat later, at ~3200 BP (95% CI 2700–3700 BP) (Supplementary Table 7). Such discrepancies may suggest a gradual separation of lineages ancestral to CAB and MOZ over thousands of years, or could otherwise reflect difficulties with coalescent-based, non-parametric methods at estimating recent demography relative to those that leverage IBD or ROH haplotypes.

### Increasing reference panel diversity using newly sequenced genomes

Previous studies have consistently demonstrated the value of including population-specific reference genomes alongside a more cosmopolitan collection of samples when imputing unobserved genotypes in target datasets^13,14^. Indeed, unsurprisingly, we observe a substantial improvement in imputation accuracy among a subset of individuals among CAB and MOZ when utilising the remaining collection of whole-genome sequences across both datasets in haplotype reference panels (Supplementary Fig. 15). It is less clear, however, whether increasing the diversity of parental African populations in reference panels would result in improvements in imputation accuracy among admixed populations from the Americas.

**Fig. 4:**
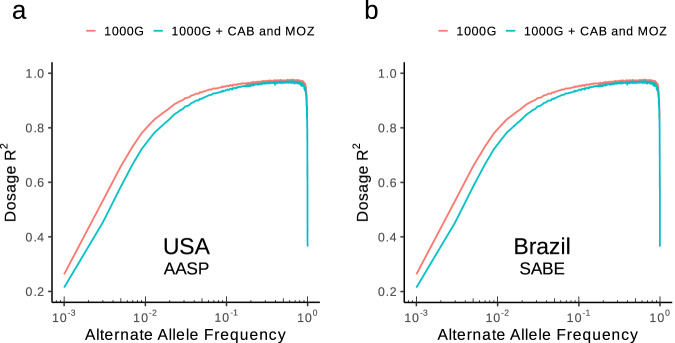
Imputation accuracy among Brazilians and African Americans from the USA after combining CAB and MOZ with the 1000 Genomes Project (1000 G) reference panel. **a** Dosage *R*^2^ (Pearson’s squared correlation coefficient) of called genotype vs genotypes imputed into African Americans from the USA (AASP)^69^ using either the 1000G reference panel only or a merged reference panel including the 1000G with and 340 newly sequenced individuals in CAB and MOZ (unrelated to the 4th degree as estimated using KING) as a function of alternate allele frequency at loci shared across both reference panels. **b** As in (**a**) but for Brazilians (SABE)^70^. When imputing genotypes using the merged reference panel, we excluded a random subset of 340 individuals from the 1000G to harmonise reference panel size with the 1000G. We emphasise that the larger sample size of CAB (291) relative to MOZ (49) means that CAB is likely to be driving these improvements in imputation accuracy.

To test this, we used two target datasets: The 23&Me African American Sequencing Project (AASP)^69^, including 2,303 individuals from the USA and the *Saúde Bem Estar e Envelhecimento* project (SABE)^70^, including 1,171 individuals from São Paulo, Brazil. ADMIXTURE clustering shows an average of 72% West African-like ancestry in individuals from the AASP (min = 30%, max = 100%, sd ± 8%) and 11% in individuals from SABE (min = 0%, max = 98%, sd ± 21%) (Supplementary Fig. 16). After masking genotypes in each dataset other than at reference coordinates present in the Illumina Omni 2.5 Array (which tags many of the SNPs present in the 1000G), we imputed genotypes using either the 1000G reference panel or a combined reference panel including the 1000G alongside CAB and MOZ. Aligned with our understanding of slave origins^22^, the addition of CAB and MOZ to the 1000G reference panel resulted in improvement in imputation accuracy in both individuals from the USA and Brazil (Fig. 4), especially for rare (0.01 < MAF ≤ 0.05: AASP 1000G *R*^2^ > 0.77, AASP 1000G + CAB and MOZ *R*^2^ > 0.80; SABE 1000G *R*^2^ > 0.72, SABE 1000G + CAB and MOZ *R*^2^ > 0.77) and very rare (MAF ≤ 0.01: AASP 1000G *R*^2^ > 0.22, AASP 1000G + CAB and MOZ *R*^2^ 0.26; SABE 1000G *R*^2^ > 0.23, SABE 1000G + CAB & MOZ *R*^2^ > 0.30) variants.

## Discussion

Analysing genomic data from under-represented human populations has the potential to shed light on major events in our species’ history and to fill important gaps in the current record of global diversity. Here, we present a collection of whole genomes from Angola and Mozambique—CAB and MOZ—expanding the coverage of catalogued genetic variation from sub-Saharan Africa, enabling insights into genetic history and potentially improving the imputation of African and African-derived ancestry in the Americas (Fig. 4).

Leveraging the power of WGS data and the well-placed diversity of ethnolinguistic groups among CAB and MOZ, we recover estimates of a genetic split between Eastern Bantu speakers from Western Bantu speakers south of the equatorial rainforests occurring around 3200 BP (CI 2700–3700 BP) (Fig. 3h) using ABC under a clean-split scenario or as early as 5900 BP using MSMC2 (Fig. 3g). Supporting the later estimate, date ranges inferred using ABC align well with the separation of Kikongo (79% of languages spoken among CAB) from Eastern Bantu around 3400 BP estimated using dated language phylogenies^18^. Intriguingly, such estimates overlap with the period spanning the earliest archaeological evidence of the Bantu speaker inhabitation of the western Congo basin around 2800 BP^21,71^, whilst notably predate the earliest evidence of Bantu speaker-associated artefacts east of Lake Tanganyika ~2600 BP^72^. However, split times inferred using genomic data should be treated with caution as they can be influenced by sequencing and phasing errors or unmodelled admixture^61,62^. Moreover, we emphasize these estimates largely predict the genetic split between Kongo peoples and Bantu speakers from north and south Mozambique. Further analyses using a greater diversity of sequenced groups under more complex demographic scenarios are therefore necessary to comprehensively assess the separation of Western and Eastern Bantu speaking populations.

After this proposed split, our results suggest heterogeneity in the demographic histories of newly sequenced Bantu speakers from Angola and Mozambique prior to recent explosions in population size, with CAB shown to have maintained a larger ancestral *Ne* relative to MOZ (Fig. 3a, b, c, d, e, f). Recent *Ne* growth among Western and South-Eastern Bantu speaking groups was similarly reported by Seidenstricker et al.^21^ and Sengupta et al.^55^ respectively, with their results indicating explosions in population size largely occurred after 1000 BP. Further patterns revealed by IBD haplotype sharing (Fig. 1c) suggest these observations of reduced ancestral *Ne* among MOZ are likely a result of serial founder events that exclusively accompanied the expansion of Eastern Bantu speakers into and across eastern and southern Africa, an observation previously unseen when analysing Y-chromosomal markers^73^ but recently reported specifically among array-genotyped Mozambicans^17^. Serial founder events are widely associated with rapid range expansions^74^. As such, these findings appear to provide a genetic parallel with the archaeological record, with a larger maintained *Ne* in Bantu speakers from Cabinda aligned with predictions of a slower, more complex settlement of the equatorial rainforests^3,21^ and a progressive reduction in *Ne* among Eastern Bantu speakers aligned with the subsequent emergence of Bantu-associated Iron Age assemblages throughout much of eastern and southern Africa in little over a millennium^4,75,76^.

Furthermore, the predicted sequence of these serial founder events (Fig. 1c), alongside additional stepwise haplotype-based analyses (Fig. 2), provide a synthesis of genetically-inferred models of the Bantu Expansion^16,17,77^, using a largely independent dataset. Under this proposed model, Bantu speaking communities south of the equatorial rainforest differentiated into branches that either continued further south into Namibia, or east into the regions surrounding Zambia (likely associated with the proliferation of Eastern Bantu languages^3,18,19^). These eastern branching dispersals through central Africa then likely further differentiated before reaching the Indian Ocean coast either moving further east towards the Great Lakes region or likely continuing east of Malawi before southward dispersals through Mozambique and subsequently into South Africa and southern Botswana (Fig. 2a). We note that any such events likely occurred well after our estimated split between CAB and MOZ, with the earliest evidence of admixture associated with these dispersals appearing well after 2000 BP (Fig. 2b).

However, whether such dispersal patterns model the initial settlement of each region is difficult to ascertain unless the distribution of present-day communities reflects the original spread of Bantu speakers across the continent. Indeed, analysis of archaeological and genetic data^21^ have revealed that spread-over-spread events were a feature of the Bantu Expansion. Notably, extending the findings presented in Wang et al.^43^ our results suggest ancient peoples inhabiting the north-western and south-eastern regions of Botswana show evidence of ancestries similar to neighbouring present-day Western and Eastern Bantu speaking groups respectively. Such observations show direct evidence that population structure reflecting the independent dispersals of Western and South-Eastern Bantu speaking groups either side of the Kalahari (Fig. 2) was apparent by at least 1100 BP. Underscoring the potential complexity of such migrations, however, whilst our proposed dates of admixture with Khoe/San groups in Tsonga and Chopi peoples among MOZ (1300 BP, Fig. 2b) overlap well with the earliest evidence complete Iron Age package in Mozambique around 1200–1600 BP^75^, these estimates predate more extensive Khoe/San admixture among present-day Bantu speakers from South Africa and southern Botswana by 550–700 years (Fig. 2). As noted in Sengupta et al.^55^ such dates are well correlated with multiple waves of Bantu speaking migration into south-east Africa evidenced by the archaeological record^20^. In light of these findings, we anticipate continued sequencing and further collective analysis of modern and ancient population samples from across sub-Saharan Africa will be essential to paint a more complete picture of the Bantu Expansion.

In conclusion, this study contributes to the ongoing effort to describe global genetic diversity and to expand our knowledge of major events in our species history. The results presented here represent another step towards understanding the genetic legacy of the Bantu Expansion, with research now beginning to paint a more complete picture of human dispersals and interactions throughout sub-Saharan Africa. We note that this study remains limited by intermediate sequencing depth and imbalanced sampling of a small number of ethnolinguistic groups and that wider sampling and higher coverage sequencing of communities across Angola and Mozambique should be prioritised moving forward. However, we hope that this data will provide a reference for future research in these regions, including medical genetic studies into phenotypic variation and disease susceptibility to aid in the continued emergence of new discoveries from Africa in the genomics era.

## Methods

### Sample collection and sequencing

This project was approved by the ethics committees of the University 11^th^ of November (“Universidade 11 de Novembro”), Cabinda, Angola (REf: UoN/2016), Pedagogic University (“Universidade Pedagógica”), Maputo, Mozambique (REf: UP/2017), and the University of Leicester ethics committee (REf: 11334-sdsb1-genetics). After obtaining full participant consent, saliva samples of individuals of both sexes and >18 years were collected from Cabinda, Angola (CAB) and Maputo, Mozambique (MOZ) and isolated at the University of Leicester. The participants provided their, their parents’, and their grandparents’ ethnolinguistic affiliation. Individuals who reported speaking the same language as their parents and grandparents were classified into major linguistic groups using the Ethnologue database (www.ethnologue.com)^24^. Isolated DNA from 300 Angolan participants and 50 Mozambicans were shipped for 15X target WGS. Reads of length 150 base pairs (bp) were generated by Illumina HiSeq X™. Four individuals from Cabinda and four individuals from Mozambique were additionally selected for high-coverage PCR-free 40X sequencing (used in MSMC2 analyses: Methods 14, see Supplementary Note 3).

### Processing sequencing data and variant calling

minimap2 v2.11-r797^78^ (mode: sx) was used to map FASTQ formatted paired-end reads generated from each newly sequenced sample against the GRCh37 reference genome (https://www.ncbi.nlm.nih.gov/assembly/GCF_000001405.13/) to align with the coordinate system used by the majority of comparative datasets used in this study (Supplementary Data 2, 3, 4). Resultant CRAM files were sorted according to linear reference coordinates, and duplicate reads were marked with samtools v1.9 markdup^79^. Base quality-scores were re-calibrated with GATK v4.0.2.1^80^ BaseRecalibrator and ApplyBQSR. Read depth statistics were generated from CRAM files using mosdepth v0.2.3^81^ with contaminated or low-quality samples removed. CRAM files were used as input for GATK v4.0.2.1 to jointly call variants across all remaining samples using the HaplotypeCaller command. This set of samples and variants were filtered and refined as described in Supplementary Note 3. Gene-based annotation of SNPs was performed using ANNOVAR^82^ utilising the GENCODE release 31 (http://ftp.ebi.ac.uk/pub/databases/gencode/Gencode_human/release_31/) and the dbSNP155 (https://ftp.ncbi.nih.gov/snp/) aligned to hg19/GRCh37.

### *f2* alleles

Shared *f2* alleles^25^ were identified using a custom R script (https://github.com/spTallman/f2) for 40 randomly selected samples from either CAB, MOZ, and all other sequenced groups within the 1000 Genomes Project Phase 3 (1000G)^12^ and the AGVP^13^. Variants within low-complexity regions (https://github.com/lh3/varcmp/raw/master/scripts/LCR-hs37d5.bed.gz) and regions of known segmental duplications (https://humanparalogy.gs.washington.edu/build37/build37.htm) were ignored.

### Dataset merging and curation

SNP genotypes generated from a filtered subset of individuals from our CAB and MOZ datasets (as described in Supplementary Data 1) were combined with either (a) genotype data from a selection of African populations sequenced and genotyped as part of the 1000G, AGVP, H3Africa-Baylor (H3AB)^15^, Simons Genome Diversity Project (SGDP)^28^ and three high-coverage ancient African whole genomes^29–31^ (Supplementary Data 2) (WGS) (b) an additional 2394 modern and ancient individuals from 261 populations across 506,721 filtered SNPs present in the Human Origins Array panel (HOA)^29,31,37–43^ (Supplementary Data 3) and (c) 3207 modern individuals from 118 populations across 276,024 filtered SNPs present across various Illumina SNP array panels^13,16,17,44–49^ (Supplementary Data 4) (ILLUMINA). Extended details of each dataset, and our merging, and quality control procedure can be found in Supplementary Note 4.

### ADMIXTURE

ADMIXTURE v1.22^27^ was applied to a matrix of genotypes from a subset of individuals from the HOA dataset (Supplementary Fig. 9). We first pruned the data to keep common sites in approximate linkage-equilibrium using PLINK v2.00a^83^ with parameters –indep-pairwise 50 5 0.5 whilst also excluding sites in regions of long-range linkage disequilibrium (LD)^84^. Ten independent, unsupervised replicates of the software were run for values *K* = 2,..,12. For each value *K*, we retain the run with the highest log-likelihood after convergence. Ancient genomes (Supplementary Data 3) were projected (-P) onto learned allele frequencies generated by modern individuals to mitigate to mitigate errors associated with aDNA degradation patterns and missingness.

### Principal Components Analysis (PCA)

We performed PCA on genotype matrices generated from our WGS, HOA, and ILLUMINA datasets using the smartpca programme (outlierremoval: NO) from the EIGENSOFT v7.2.1 tool-suite^32^. The HOA dataset was subsampled to enrich for African groups (Supplementary Fig. 6). Rare and LD correlated SNPs were removed a priori using PLINK v2.00a^83^ with parameters –indep-pairwise 50 5 0.5 –maf 0.05. SNPs is regions of known long-range LD^84^ were also removed. Modern individuals were used to construct eigenvectors and least-squares projection (lqproject: YES) was performed to overlay data from ancient genomes present in the WGS and HOA datasets, with shrinkmode: YES used to mitigate errors associated with aDNA degradation patterns. We additionally performed PCA on the WGS dataset after down sampling each group to a maximum of ten randomly selected individuals.

### *f4 s*tatistics

We calculate *f4* using the R package admixr^85^ across all possible (non-redundant) three-population arrangements of groups in our WGS dataset with panTro5 (https://www.ncbi.nlm.nih.gov/assembly/GCF_000001515.7/) set as the outgroup. Groups were sub-sampled to a maximum of ten randomly selected individuals. Significance (Z-scores) and standard errors are estimated using a weighted block-jacknife over segments of 5-centimorgans (cM).

### Identity by Descent (IBD)

IBD haplotypes were estimated across all Niger-Congo speakers in our WGS dataset using IBDSeq^34^ with default parameters, after filtering for SNPs segregating with a MAF > 0.01 in each group. We removed gaps between IBD segments that have at most one discordant homozygote and are <0.6 cM in length as well as IBD segments in regions of low SNP-density. Significant (directional) differences in the mean pairwise IBD sharing between groups were inferred using a one-tailed permutation test. Correlations between geographic distance and within-population IBD sharing among Bantu speakers outside of CAM were performed with CAB selected as the reference point owing to its proximity to original staging location for the dispersal of Eastern Bantu speakers proposed under a late-split model^19^. Distances (km) from Cabinda were calculated with approximate latitude and longitude coordinates for each group (Supplementary Table 3) using the geosphere R package (https://github.com/rspatial/geosphere).

### Runs of Homozygosity (ROH)

ROH were estimated across all Niger-Congo speakers in our WGS dataset after filtering for SNPs segregating with a MAF > 0.01 in each group using PLINK 1.9^83^ with parameters --homozyg-snp 50 --homozyg-kb 300 --homozyg-density 50 --homozyg-gap 1000 --homozyg-window-snp 50 --homozyg-window-threshold 0.05.

### CHROMOPAINTER

After phasing genotypes using SHAPEITv2^86^ alongside the 1000 G reference panel (https://mathgen.stats.ox.ac.uk/impute/1000GP_Phase3/), and following the stepwise procedure to estimate global mutation/emission (-M) and switch rate (-n) parameters as outlined in previous studies^37,87^ CHROMOPAINTERv2^50^ was run on all diploid individuals within either our HOA or ILLUMINA datasets using two distinct donor-recipient population configurations: (i) all individuals and/or groups in the dataset are included as both recipients and donors of shared haplotypes (using the -a flag, HOA all-copying model and ILLUMINA all-coping model) and (ii) (specifically for the HOA dataset), as in (i), but with all Bantu speaking groups other than the Cameroonian Lemande (used to describe Bantu speaker related ancestry in previous studies^29,41^) excluded as donors (HOA no-Bantu-copying model).

### fineSTRUCTURE

fineSTRUCTURE v2.1.3^50^ was run independently on both (a) Niger-Congo speakers within our HOA dataset (Supplementary Fig. 8a) using the chunk counts sharing matrix output from the HOA all-copying model, and (b) Niger-Congo speakers within out ILLUMINA dataset (Supplementary Fig. 8b) using the analogous chunk counts matrix output from the ILLUMINA all-copying model. For each analysis, we sampled cluster assignments every 10^5^ iterations across 10^6^ total MCMC iterations after 10^6^ burn-in steps. All other individuals were fixed int super-populations (Supplementary Data 3, 4). We next performed an additional 10^5^ hill-climbing iterations, starting from the MCMC sample with highest posterior probability. This resulted in a classification of 61 clusters in the HOA dataset and 126 clusters in the ILLUMINA dataset that were each subsequently merged into trees using fineSTRUCTURE’s greedy algorithm.

### SOURCEFIND

For individuals in all African groups in our HOA dataset (Supplementary Data 5), we first used the Bayesian mixture modelling approach employed by SOURCEFINDv2^51^ to identify the relative proportions of ancestry that each individual shares with each given donor group using the chunk lengths sharing matrix output from the HOA no-Bantu-copying model with all donor populations provided as possible surrogates. We then performed a second, stepwise analysis using the chunk lengths matrix from the HOA all-copying model whereby individual ancestry proportions were estimated by SOURECEFINDv2 multiple times across all African groups in the dataset and specifying a cumulatively growing number of additional Bantu speaking donor groups as possible surrogates in each step (see Supplementary Note 5), we report results from those with evidence of Bantu-related ancestry more closely related to CAB than Cameroonian Bantu speakers in Fig. 2. For all runs of SOURCEFINDv2 the truncated Poisson prior on the number of surrogate groups that contribute ancestry to each target individual to was fixed to four, allowing eight total groups to contribute some proportion of ancestry at each MCMC iteration. We ran 200,000 total MCMC iterations and 50,000 burn-in steps, sampling mixture coefficients every 5000 iterations. Final ancestry proportions are reported as the average of these mixture coefficients across all posterior samples.

### fastGLOBETROTTER

For all 39 African populations in our HOA dataset with evidence of ancestry closer to CAB than Cameroonian Bantu speakers (Supplementary Data 5), we also used fastGLOBETROTTER^52,53^ to estimate admixture. Specifically, fastGLOBETROTTER requires both chunk lengths sharing matrices and individual painting sample files as inputs. Thus, to avoid self-copying between individuals within their own population, which may mask signatures of recent admixture^87^, we use painting sample files generated for each target population generated by re-running CHROMOPAINTERv2 for each target population whilst providing all other populations as donors – excluding very closely related individuals from different ethnolinguistic group labels that cluster together using fineSTRUCTURE (Supplementary Fig. 8a)—and using the same global mutation/emission and switch rate parameters as estimated with CHROMOPAINTER (Methods 10). Chunk length sharing matrices were generated using the original CHROMOPAINTERv2 run under the HOA all-Bantu-copying model. For each target population, fastGLOBETROTTER was then run after specifying as surrogates those donor groups modelled by SOURECEFINDv2 to contribute >1% ancestry to the target population. For each fastGLOBETROTTER run, we performed five iterations of the algorithm, generating p-values and 95% confidence intervals using bootstrap re-sampling of groups over 100 replicates. As recommended, we report results with the null.ind parameter set to 1 to avoid inference based on spurious decay signals not attributable to genuine admixture. To gain further insights into the specific donor populations being used as distinct admixing sources, we performed a visual inspection of the coancestry curves generated for each population with strong evidence of admixture and report those with an *R*^2^ > 0.5.

### qpAdm

We used the R package admixr^85^ to model admixture among Botswana_Taukome_1100BP. Botswana_Xaro_1400BP^43^, and South_Africa_400BP^31^ using the *qpAdm* command to perform two-way admixture tests. Specifically, we test combinations of the reference populations: SA_Ovambo, Tswana, Kgalagadi, BSZ, MOZ (north), MOZ (south), with South_Africa_12000BP^38^ and Ballito Bay A^31^ as source groups, with Mende (MSL), Mbuti, Khomani, Dinka, Iran_Neolithic, Levant_Neolithic, Ami, Karitiana, Punjabi, Onge, French, Sardinian groups present in our extended HOA dataset (Supplementary Data 3) as reference groups. Statistically significant model fits were taken as those with a *p* value > 0.05, with implausible models involving negative ancestry proportions discarded. All calculations were performed using transversion sites only to mitigate errors associated with aDNA degradation patterns.

### MSMC2

MSMC2^61,62^ was used estimate within-population (four individuals per population) and cross-population (two individuals per population) coalescence rates using high-coverage (mean autosomal read depth of ~37X) genomes representing individuals from both the CAB and MOZ (Supplementary Note 3) as well as Niger-Congo groups (Yoruba, Mende, Mandenka, BantuKenya, BantuTswana) sequenced as part of the SGDP^28^. SNP calls and coverage masks for each genome were generated directly from sample-specific BAM files using the bamCaller.py script from the MSMC GitHub repository (https://github.com/stschiff/msmc-tools) and subsequently phased using SHAPEITv2^86^ alongside 1000 G reference panel (https://mathgen.stats.ox.ac.uk/impute/1000GP_Phase3/). Following recommendations, we use sample-specific masks to exclude genotypes present in regions of low coverage relative to the genome-wide average with coverage statistics generated using mosdepth^81^. We also exclude genotypes across all samples using Heng Li’s universal mask^28^. The mutation rate used to scale time was 1.25 ×10−^8^ per base-pair per generation^88^.

### Relate

Relate v1.1^63^, was used estimate genome-wide genealogies using 40 subsampled genomes from CAB, 13 from MOZ (north), and 27 from MOZ (south). Genotypes were phased using SHAPEITv2^86^ and filtered at sites marked as “not passing” in the 1000G accessible genome pilot mask (ftp://ftp.1000genomes.ebi.ac.uk/vol1/ftp/release/20130502/supporting/accessible_genome_masks/StrictMask/). The 6-EPO multiple alignment estimation of the human ancestral genome (http://ftp.1000genomes.ebi.ac.uk/vol1/ftp/phase1/analysis_results/supporting/ancestral_alignments/) was used to identify the most likely ancestral allele for each locus. Within-population and cross-population coalescence rates were calculated using the EstimatePopulationSize.sh script from the Relate GitHub repository (https://myersgroup.github.io/relate/).

### Approximate Bayesian Computation (ABC)

We first define a simple, two-population split model (Supplementary Fig. 12) describing a clean-split between two populations. Here populations *P*_*CAB*_ and *P*_*MOZ*_ represent populations of Bantu speakers from the CAB and MOZ datasets respectively. Moving backwards-in-time, for each population *P*_*CAB*_ and *P*_*MOZ*_, population *P*_*n*_ is initialised with a diploid effective population size of *N*_n_ *at Generation Present* and an exponential growth/decay rate of *α*_n=_log(*N*_n_*/N*_n_^’^)*/Generation Split*, where *Generation Split* is defined as the generation at which populations *P*_*CAB*_ and P_*MOZ*_ merge (i.e., lineages can coalesce freely) to form *P*_*Ancestral*_ and *N’*_*n*_ is the diploid effective population size of *P*_n_ at *Generation Split*.

To reduce computation time, we additionally include two fixed parameters wherein the merged population *P*_*Ancestral*_ instantaneously changes to a diploid population size of 12,000 (*N*_*Fixed*_) at generation 7586. Values were based on the model of human population history presented in Tennessen et al.^89^. Using the coalescent simulator msprime^65^, we generated 135,000 simulations of chromosome with values for each parameter randomly drawn from their corresponding prior distributions (Supplementary Table 5). A mutation rate of 1.25 × 10^−^^8^ per base-pair per generation was selected and variable recombination rates across the ~249 Mb sequence of chromosome 1 were input using inferred genetic distances between sites. Simulated tree sequences were subsequently converted into phased VCF files. To additionally simulate genotyping error rates associated with low or intermediate coverage sequencing data, we also applied a genotype error rate of 0.001^34^ independently for each simulated VCF. Specifically, error was introduced by converting homozygote genotypes to heterozygote and by converting heterozygote genotypes to a randomly chosen homozygote using a custom R script (https://github.com/spTallman/vcfErr). As our observed data, we used 40 randomly sub-sampled genomes from CAB and 40 randomly sub-sampled genomes from MOZ. This data was subsequently restricted to ~1.5 million biallelic SNPs present on chromosome 1, segregating in these 80 individuals and phased using SHAPEITv2^86^. For every simulated and observed VCF, we calculate a set of 46 summary statistics as described in Supplementary Table 5 (adapted from Gladstein et al.^67^).

To estimate posterior distributions and median point estimates of each demographic parameter value, we use the ‘neuralnet’ method implemented as part of the R-package *abc*^64^ with a logit transformation applied to each parameter. We assess the accuracy of the median points of the posterior distributions by calculating the Mean Absolute Error, Mean Squared Error and Root Mean Squared Error using the *Metrics* R-package (https://github.com/mfrasco/Metrics) by comparing with pseudo-observed parameter values from 1000 randomly selected simulations (Supplementary Table 7). We further ensured posterior distributions captured true uncertainty in parameter estimates by calculating the frequency with which pseudo-observed parameter values associated with 1000 randomly selected simulations appear within the 2.5 and 97.5 percentile bounds of their corresponding posterior distributions. We found that 4 neurons in the hidden layer and a 10% tolerance level minimised the average prediction error. Finally, we use our observed summary statistics computed using SNP data from the subsampled CAB and MOZ genomes alongside the complete set of 135,000 simulations from the clean-split model to calculate posterior distributions and median point estimates independently for each parameter (Supplementary Table 7).

### Imputation

As our target datasets for imputing genotypes, we use phased, biallelic, autosomal SNPs from 2301 self-reported African Americans from the USA sequenced as part of the AASP^69^ and 1171 Brazilians sequenced as part of SABE^70^. To ensure compatibility with reference panels, we use UCSC LiftOver alongside the hg38toHg19 chain file (https://hgdownload.cse.ucsc.edu/goldenpath/hg38/liftOver/hg38ToHg19.over.chain.gz) to convert AASP and SABE SNP coordinates from hg38 (https://www.ncbi.nlm.nih.gov/assembly/GCF_000001405.26/) to hg19/GRCh37. For a third analysis, we also used 50 randomly sub-sampled individuals from CAB and 10 randomly subsampled individuals from MOZ as additional target dataset to test improvements in imputation accuracy afforded by the addition of CAB and MOZ to reference panels. Prior to imputation, SNPs across all three target datasets were phased using SHAPEITv2^86^ and masked at all-but ~2.5 million autosomal loci present in the Illumina Omni 2.5 array panel (selected as this panel is optimised to tag SNPs uncovered as part of the 1000G, https://emea.illumina.com/products/by-type/microarray-kits/infinium-omni25-8.html). African ancestry proportions in SABE and the AASP were estimated using ADMIXTURE after merging data from either cohort with genotype data from the 1000G individuals from our newly sequenced CAB and MOZ datasets and applying the same procedure as outlined in Methods section “ADMIXTURE”. Haplotypes were split into 5 Mb chunks and provided in parallel to the IMPUTE2^90^ software to impute reference panel genotypes using either (a) the 1000G Panel (b) the 1000G reference panel merged with 340 CAB and MOZ genomes (all biologically unrelated individuals shown in Supplementary Data 1) using the –merge_reference_panels command. To ensure any difference in imputation accuracy was not simply the result of increased reference panel size, we randomly excluded 340 individuals from the 1000 G reference panel when performing imputation using the merged panel. When imputing genotypes into the 60 randomly sampled target individuals from the CAB and MOZ datasets specifically, these individuals were removed from the reference panels that included CAB and MOZ. Imputed genotypes with an INFO score (*r*^2^ < 0.3) were filtered out. As a metric of imputation accuracy, for each reference panel across the set of loci present across both panels, we calculate Pearson’s Correlation Coefficient (Dosage R^2^) using imputed genotype dosages across the target dataset and the original, unmasked genotypes as a function of non-reference allele frequency.

### Reporting summary

Further information on research design is available in the Nature Portfolio Reporting Summary linked to this article.

### Supplementary information

Supplementary Information

Description of Additional Supplementary Files

Supplementary Data 1-5

Reporting Summary

## Data Availability

Individual-level sequence datasets (compressed BAM files or CRAM files) and variant calling datasets (VCF files) generated in this study have been deposited at the European Genome-phenome Archive (EGA) under EGA data accession number EGAD00001011992. This data is allowed for general research use, including health/medical/biomedical purposes and other biological research such as the study of population origins or ancestry. Access to this dataset is contingent to signing a Data Access Agreement (DAA) with the University of Leicester. Conditions of access, including timeframe to response to requests and details of any restrictions imposed on data can be obtained from EGAC00001003360. Corresponding data on ethnolinguistic group is reported on Supplementary Data 1. AGVP genomic data is available in EGA under accession code EGAD00001001663. H3A data was obtained is available in EGA under accession codes: EGAD00001004220; EGAD00001004316; EGAD00001004393; EGAD00001004533; EGAD00001004505; EGAD00001004334; EGAD00001004557; and EGAD00001004448. Three high-coverage African Ancient Genomes were obtained from https://www.ebi.ac.uk/ena/browser/view/PRJNA295861; https://www.ebi.ac.uk/ena/browser/view/PRJEB22660; https://reich.hms.harvard.edu/datasets. The SGDP was obtained from https://reichdata.hms.harvard.edu/pub/datasets/sgdp/. The HOA was obtained from https://reich.hms.harvard.edu/datasets; https://ega-archive.org/datasets/EGAD00010002100; https://www.ebi.ac.uk/ena/browser/view/PRJEB36063. The ILLUMINA dataset was obtained from https://ega-archive.org/datasets/EGAD00010000965; https://ega-archive.org/datasets/EGAD00010000496; https://www.ebi.ac.uk/biostudies/arrayexpress/studies/E-MTAB-8450; https://datadryad.org/stash/dataset/doi:10.5061/dryad.bs06h; http://sbimb.core.wits.ac.za/data/SNPgenotyping_01.html; http://mega.bioanth.cam.ac.uk/data/Ethiopia; https://ega-archive.org/datasets/EGAD00010000616; https://www.ebi.ac.uk/biostudies/arrayexpress/studies/E-MTAB-1259; https://github.com/bmhenn/khoesan_arraydata. AASP genomic dataset was obtained from dbGAP: dataset no. phs001798.v2.p2, and SABE genomic dataset was kindly provided by the authors but can now be found at from EGA under accession number EGAD00001008640.

## Code availability

Custom R scrips used to identify *f2* alleles and to additionally simulate genotyping error rates can be found in https://github.com/spTallman/.
