# Supplementary material for: Whole genomes from Angola and Mozambique inform about the origins and dispersals of major African migrations
Source: Nat Commun. 2023 Dec 2;14:7967. doi: 10.1038/s41467-023-43717-x (PMC10693643; doi:10.1038/s41467-023-43717-x)
Supplement: Supplementary file 1 — Supplementary Information [file 41467_2023_43717_MOESM1_ESM.pdf]

Supplementary Notes

Supplementary Note 1. DNA Extraction & Sequencing

After obtaining full participant consent, 300 saliva samples were collected from Cabinda, Angola (henceforth referred to as CAB) and 50 from Maputo, Mozambique (henceforth referred to as MOZ) using the Oragene® DNA saliva kit (Catologue # OG-610; DNA Genotek) collection kit. All sampling was performed in agreement with the Helsinki accords Section 4c (https://www.osce.org/files/f/documents/5/c/39501.pdf). DNA was extracted with prepIT•L2P according to manufacturer's manual protocol from 0.5ml of sample (Catologue # PT-L2P-45; DNA Genotek), with minor deviations. RNA was degraded via introduction of a 30-minute, 37°C incubation with 10mg/ml RNAseA (Catalogue# EN0531; Thermo Fisher Scientific) directly after initial denaturation and DNA release incubation, centrifugation times increased to 5-minutes, ethanol volume increased to 1.5X supernatant volume for DNA precipitation, ethanol wash strength increased to 85% and final resuspension in 10mM Tris HCl pH8.5 (Catalogue # 80526, Sigma Aldrich).

Aliquots holding 2-5ug of isolated DNA from 300 CAB participants were shipped to NGX Bio (URLs) for intermediate-coverage whole genome sequencing. DNA from 50 MOZ participants were shipped to Novogene (URLs) for intermediate-coverage, whole-genome sequencing. Reads of length 150 bp were generated by Illumina HiSeq X in both cases, totaling 15,761 gigabases (Gb) of raw sequence data. Paired end reads in FASTQ format were received for all 350 samples (Supplementary Data 1).

Supplementary Note 2. Sample Processing and Quality Control

2.1 Read Depth

minimap2 v2.11-r797 (sx mode)[1] was used to map FASTQ format-ted paired-end reads generated from each sample against the human reference genome build hg19/GRCh37 (URLs). Resultant BAM files were sorted according to linear reference coordinates, and duplicate reads were marked with samtools v1.9 mark-dup[2]. Base quality-scores were re-calibrated with GATK v4.0.2.1[3] BaseRecalibrator and ApplyBQSR; using the most recent (as of 9th May 2018) build of the dbSNP155 (URLs) as the set of known variants upon which to build the recalibration/covariation model applied to our sorted BAM files. BAM files were subsequently merged at the individual level, converted to CRAM format and then further compressed with crumble v0.8.1[4] using default (compression level 9) parameters.

Read depth statistics were generated from CRAM files using mosdepth v0.2.3[5] and the sex of each sample was confirmed by comparing the ratio of coverage between the X and Y chromosomes

(X/Y ratio). We found two individuals whose estimated sex was ambiguous or significant outlying compared to the average female or male X/Y ratio. We take this as evidence of cross-contamination between samples. A further three individuals had an estimated sex which disagreed with their given sex. We find no evidence for sample-duplication or contamination in either of these three samples and as such, they were included in all downstream analyses.

After read alignment and duplicate removal, the average autosomal read depth for intermediate-coverage samples was calculated to be 11.56X for CAB and 12.07X for MOZ respectively. Across both sets of individuals, an average of 93.94% +/-2.86 of total reads mapped to the reference genome, validating DNA collection from saliva as a means of collecting high-quality WGS data from human samples. As notable exceptions, two samples had significantly lower read depth and a higher percentage of unmapped reads than expected, likely due to contamination with pathogenic bacterial DNA during sample collection (see below). These samples were removed from the dataset. The remaining samples had a genome-wide per-site read depth which followed an expected negative-binomial distribution.

2.2 Contamination

To further investigate potential cross-sample/human DNA contamination, we used VerifyBamID2[6] to calculate ancestry agnostic estimates of contamination fractions per sample (α) using a training set of 100,000 sites from the 1000 Genomes Project Phase 3 (1000G)[7]. After applying the algorithm to each individual BAM file, we find two samples which show elevated levels of contamination (α: 0.12 and 0.32; previously flagged above). Both were removed from further downstream analyses along with one individual exhibiting extremely low coverage. The remaining 347 samples had an estimated contamination level of <1%.

2.3 Unmapped Reads

Recent studies have shown that whole-genome sequencing libraries extracted from saliva samples provide a potentially rich source of information regarding the microbial composition of the oral cavity. In order to assess the origin of potentially contaminating bacterial sequences present in our dataset, we used KrakenUniq[8] to perform k-mer based metagenomic classification of reads for which failed to map to the human reference genome. Prior to running KrakenUniq, samtools v1.9 was applied to sample-specific CRAM files to extract high-quality (Q>30) reads for which both mate-pairs were not aligned to the reference build hg19/GRCh37. Reads were then remapped to hg38/GRCh38 (URLs) and an additional 125,715 contigs (296Mb total) recovered as part of an extended African human pan-genome[9]. FASTQ files containing all remaining unmapped reads (UMRs) were used as input for KrakenUniq, and taxa-abundance estimates for each sample were generated by comparing sequences with a 31-mer indexed database comprised of all complete microbial genomes deposited in RefSeq (URLs).

As in earlier studies[10], we find unmapped reads generated as part of our sequencing efforts -represent an abundance of known colonizers of the oral cavity. The most represented genera estimated across each individual sequencing library are Prevotella (mean 5:64 3:5% UMRs) and Haemophilus (mean 5:4 3:2% UMRs) followed by Streptococci (mean 4:5 2:6% UMRs) and Neisseria (mean 4:1 2:2% UMRs), with the remaining UMRs mapping to a further 420 unique microbial genera. Interestingly, we find that the two samples which appeared as significant outliers with regards to the proportion of reads mapped to the human reference were also inferred by KrakenUniq to have by far the highest number of non-human reads derived from a single bacterial species. In one individual, 305,905,813 (77.23% of total UMRs) reads mapped to *Stenotrophomonus maltophilia* and in the other 226,791,973 (73.51% of total UMRs) mapped to *Klebsiella varicola* both of which are pathogens known to cause upper-respiratory tract infections in humans[11, 12]. Although all precautions were taken prior to this study to avoid sampling from such individuals, these results clearly highlight the fact that sampling saliva in patients who are, for example, suffering from upper-respiratory tract infections is highly unreliable for use in genomic sequencing projects.

Supplementary Note 3. Variant Calling, Filtering & Annotation

3.1 Autosomes & X-chromosome

CRAM files were used as input for GATK v4.0.2.1[3] to jointly call variants across all 347 remaining samples sequenced to approximately 12X autosomal coverage (Supplementary Data 1) following the Best Practices Pipeline (URLs) modified where necessary to fit our intermediate-coverage sample set. Specifically, GVCF files for each sample were generated using the HaplotypeCaller command. The human reference genome was then split into 348 8Mb contigs (shorter at telomeres) covering autosomes and the X-chromosome and used to parallelize the collection and genotyping of all samples using GenomicsDBImport and GenotypeGVCFs respectively. The resulting set of raw, unfiltered variant calls included 37,048,597 Single-Nucleotide-Polymorphisms (SNPs) (Ts/Tv= 2.00), 9,681,152 short (<150bp) insertions or deletions (INDELs) with 3,903,720 observed multi-allelic sites.

To filter out spurious variant calls, e.g. potential sequencing errors or mapping artifacts, we applied the following quality-control procedure (in order):

1. Variants with significant departure from Hardy-Weinberg equilibrium in the direction of excessive heterozygosity (p <3.4e-06, phred-scaled >54.69) using Fisher's exact test were filtered out. Such variants have been shown to be associated with genotyping error.
2. For SNPs, the Variant Quality-Score Recalibration (VQSR) functionality of GATK v4.0.2.1 was applied with recommended features using the training sets: (1) HapMap 3.3 (truth = true, training = true, known = false, prior 15.0) (2) Omni 2.5 overlapping with

1000G Phase 1 (truth = true, training = true, known = false, prior = 14) (3) 1000G Phase 1 call-set (truth = false, training = true, known = false, prior = 12.0) (4) dbSNP150 (truth = false, training = false, known = true, prior = 2.0) in -mode SNP. A tranche-sensitivity value of 99.5 was selected and variants outside of this cut-off were removed.

3 For short INDELs, the VQSR functionality of GATK v4.0.2.1 was applied with recommended features using the training sets: (1) Mills and 1000G gold-standard Indel set (truth = true, training = true, known = false, prior 12.0) (2) Axiom Poly Exome Plus Array set (truth = false, training = true, known = false, prior = 10.0) (3) dbSNP150 (truth = false, training = false, known = true, prior = 2.0) in -mode INDEL. A tranche-sensitivity value of 99 was selected and variants outside of this cut-off were removed.
4 Variants with >2 variants at a single position (multi-allelic) were removed i.e., only biallelic REF/ALT variants were processed. This includes VCF 4.3 (https://samtools.github.io/hts-specs/VCFv4.3.pdf) formatted spanning deletions (as represented by * in VCF files) as well as overlapping INDEL calls.
5 Variants with >10% missingness across our sample-set were removed.

After filtration, a total of 29,076,696 SNPs (Ts/Tv=2.05) and 3,767,719 INDELs (67% of which were single point changes) present along the autosomes remained along with 885,705 SNPs and 156,559 INDELs on the X-chromosome. Note that uniparental markers (Y-chromosome and mtDNA) were processed separately as outlined in Methods 8.

3.2 Genotype Refinement

It has been previously shown that exploiting correlation in linkage-disequilibrium (LD) between large numbers of individuals alongside genotype uncertainty can improve the quality of genotypes by correcting calls in disagreement with the underlying haplotype[13].We calculated posterior genotype probabilities (GP) using Beagle v4.1[14] with default parameters, using phred-scaled genotype likelihoods (PLs) generated by GATK v4.0.2.1 HaplotypeCaller[3] at 29,962,401 biallelic, genome-wide SNPs and 3,861,191 INDELs which passed our filtration criteria. Male heterozygous (REF/ALT) PLs on the non-pseudo autosomal regions (PAR) of the X-chromosome were truncated to avoid spurious diploid calls. To speed up computation, we parallelized Beagle v4.1 across windows of 25,000 sites with a 1,500-site overlap and subsequently merged generated VCF files. PLINK formatted genetic maps for reference build hg19/GRCh37 used in this processing step were downloaded from the Beagle webpage (URLs). As we find that INDELs across our dataset likely contain a high proportion of false-positives due to the difficulty of calling these variants in intermediate-coverage sequencing data[15] we only retain INDELs with a posterior genotype probability >0.9.

3.3 Heterozygosity Comparison between Collection Sites

To compare the average genomic diversity of individuals from the CAB and MOZ groups, we calculated to average autosomal heterozygosity ratio across individuals within the two datasets and tested for differences between collection sites using linear regression controlling for the small negative correlation between heterozygosity ratio and coverage (spearman's Rho = -0.11, p = 0.04) in the form lm(het. Ratio ~ collection site + autosomal coverage) (R: URLs) and then calculated p-values using bootstrap resampling (boot_summary from the boot.pval R package, URLs).

3.4 Inaccessible Genome Mask

Not all positions across the human genome are equal in terms of relative sequence content or uniqueness. Thus, certain regions of the reference genome may be inaccessible to short-read sequencing technologies and therefore exhibit significantly variable genotyping properties. To evaluate study-specific genomic accessibility, we generated a set of "not-passing" bases using a procedure adapted from the 1000G. Specifically, the base is inferred to be "not-passing" if (1) The reference base is an N. (2) The mean study-wide coverage at that base is less than half the average. (3) The mean study-wide coverage at that base is more than double the average. (4) More than 20% of the reads at that base have a mapping quality (MQ) of 0. After applying the above criteria, we infer a total of 260,731,626 autosomal bases (9% of total) across the autosomes as being "not-passing" or inaccessible based on a study-wide autosomal average read depth of 4461X calculated using mosdepth v0.2.3[5] and a further 9,131,659 bases (6% of total) across the X-chromosome (evaluated separately due to haploid males) based on an average read depth of 3400X. Across the entire call set, we find only 50,607 (0.2%) called SNPs fall outside of our accessible genome mask, suggesting our variant calling and filtration procedure was reasonably effective in removing potentially spurious variant calls within these regions. Although we do not explicitly filter out these loci from our release, we do exclude these variants from all downstream population genetic analyses, as such regions are likely to be enriched for false positives.

3.5 SNP Density

To further validate our variant-calling procedure, SNP density across the autosomes was computed using VCFTOOLS v1.014[16] using non-overlapping bins of 100kb. When comparing the normalized SNP density observed in our dataset to that of the 1000G (Pearson's $r^2 = 0.92$) or the AGVP (Pearson's $r^2 = 0.97$) we find a high correspondence. The lower correlation observed with the 1000G is likely due to variant calling being performed across a larger, more cosmopolitan set of samples relative to this study and the AGVP.

3.6 Variant Annotation

3.6.1 Novel Variation

To assess the novelty of variation across our sequencing dataset, we annotated all SNPs, autosomal, X-chromosomal) using the dbSNP155 (URLs) with the bcftools v1.9[17] annotate command excluding SNPs in low-complexity regions of the human genome that are more likely to be sequencing errors. This resulted in a final list of 2,857,384 novel SNPs on the autosomes and 37,958 novel SNPs on the X-chromosome. Similarly, we compared the approximately 29 million autosomal SNPs present in our call set with three additional, external datasets: the 1000G (8X), sequencing data from the AGVP (4X) (Supplementary Data 2), and high-coverage (30X) sequencing data from the H3Africa-Baylor study[18] genotyped and filtered as described below (section 4.2).

3.6.2 Ancestral States

Ancestral states were inferred for all 29 million autosomal SNPs with the fill-aa tool from the VCFTOOLS v1.014 suite using the 6-EPO multiple alignment estimation of the human ancestral genome released as part of the 1000G (URLs). To explore the distribution of this variation, and further validate our SNP calling procedures, we generated derived Allele Frequency Spectra (AFS) of SNPs with high-confidence ancestral calls using VCFTOOLS v1.014 – counts –derived across 40 randomly sampled, unrelated individuals from CAB or MOZ (with no recent European ancestry as estimated by ADMIXTURE, see Supplementary Data 1) and compared this to the derived AFS of other Niger-Congo speaking populations (and British, GBR) sequenced as part of the 1000G or the AGVP finding expected distributions corresponding closely with other African populations, with an abundance of rare variants relative to GBR.

3.7 Y-Chromosome

As in Poznik et al.[19], we first define accessible regions of the Y-chromosome using 184 male-specific CRAM files present in our study (see Methods 3.1) by emitting sites whereby (1) Read-depth deviated from a study-wide exponentially weighted moving average (EWMA) estimated across contiguous 1kb intervals (2) More than 10% of the reads at that site had a MQ of 0.

After applying the above procedure, we called SNPs across 11.6 MB of accessible Y-sequence using GATK v3.8 Unified Genotyper[3] with additional flags –stand_call_conf 30 -mbq 30 -gt mode DISCOVERY. Heterozygous genotype calls were set to missing and we subsequently filtered out sites with greater than 10% missingness across the sample set. The median accessible study-wise read depth was 1259X (as calculated by mosdepth v0.2.3) and sites with a depth of more than three median absolute deviations above or below this value were excluded along with multi-allelic sites. A total of biallelic 13,091 SNPs remained, with 3,352 included in the most recent (as of 5th July 2020) International Society of Genetic Genealogy (ISOGG) database (URLs). We used Yhaplo[19] to assign haplogroups to each sample and compared this with haplogroups called in 1,233 males from the 1000G, estimated using the same procedure.

Analysis of paternally inherited NRY indicates low diversity among CAB and MOZ, with 84% of CAB and 75% of MOZ estimated as carrying the E1b1a haplogroup associated with the Bantu

expansion[20] (Supplementary Figure 15). Worthy of note, however, is the appearance of two basal R1b1* haplogroups (~1% frequency) among Kikongo speakers (Supplementary Data 1) (previously found among Angolan speakers in Brito et al.[21]), which had previously been attributed demographic events in central-Africa unrelated and prior to the Bantu expansion[20].

3.8 Mitochondrial DNA

mtDNA sequences from 340 high-quality, biologically unrelated (see section 3.9) samples were processed using a consensus calling strategy similar to the one employed in Li et al[22]. Specifically, reads not mapped to the nuclear genome (hg19/GRCh37) were remapped to the Revised Cambridge Reference Sequence (URLs) to a mean depth of 1136X. Consensus sequences were called using the majority rule, requiring minimum base quality of 30 with N (no call) generated if the alternative allele frequency was greater than 0.3. Resultant whole mtDNA FASTA files were subsequently aligned along with 2,534 sequences from the 1000G using MAFFT v7.222[23]. Haplogrep2 v2.1.1[24] was used to predict haplogroups with PhyloTree build 17[25] and maximum-likelihood based phylogenetic trees were constructed using FastTree v2.1[26] with flags -nt -gtr -spr 4.

Owing to the greater diversity of maternal lineages in ancestral West African populations[27], mtDNA haplogroup variation is substantially greater than that observed for the Y-chromosome, with L0a (CAB: 10%, MOZ: 26%), L1b (CAB: 4%, MOZ: 2%), L1c1b (CAB: 2%, MOZe: 2%), L1c2 (CAB: 10%, MOZ 2%), L2a (CAB: 10%, MOZ 34%), L2c (CAB: 1%, MOZ: 2%), L3b (CAB: 6%, MOZ: 4%), L3d (CAB: 4%, MOZ: 10%), L3e (CAB: 26%, MOZ: 14%) segregating moderate to high frequencies across both datasets (Supplementary Figure 14). The present-day distribution of such groups has been previously attributed to the migration of Bantu-speaking populations across Africa[28, 29].

As per the lower observed levels of Khoe/San related ancestry across the autosomes relative to other Southern African Bantu-speaking populations (e.g., Zulu, BOT – see Figure 1b), we find only a single HG-related mitochondrial haplogroup (L0d2c1) among all sequenced individuals in MOZ (Supplementary Data 1), with previous studies suggesting HG-related haplogroup frequency of 22-50% in South African groups such as the Zulu[30]. That is, despite previous studies indicating sex-biased mating practices between Bantu-speaking migrants and autochthonous populations in the South of the continent[31]. Such findings may suggest that substantial genetic exchange between expanding farmers and Khoe/San populations primarily occurred as Bantu speaking groups moved further south beyond the region surrounding present-day Mozambique. The presence of rainforest hunter gatherer -related L1c1a (7%), L1c4 (~1%) and L1c5 (~1%) haplogroups in CAB aligns with previous suggestions of gene-flow between Bantu speakers and ancient central-African forager populations over thousands of years[27] and our inference of rainforest hunter-gatherer like admixture among the Kongo peoples in CAB (see Supplementary Figure 7).

3.9 Familial Relatedness Estimation

We used the KING-robust algorithm[32] implemented in PLINK2[33] (URLs) to estimate cryptic relatedness or sample-duplication between all pairs of individuals in our intermediate-coverage sample-set, filtering relationships <4th degree. We first removed all variants within (1) Our accessible genome mask (see Methods 5.3). (2) Heng Li's Low-Complexity Region (LCR) mask (URLs). (3) The hg19 ENCODE blacklist (URLs). (4) Segmental duplications from UCSC (URLs) and then filtered SNPs in high-LD using –indep-pairwise 50 5 0.05. 8 pairs of individuals (one from MOZ, seven from CAB, with one individual being independently related to two others) were estimated as being 3rd relatives or above. We took the minimal set of unrelated individuals, resulting in a final set of 49 biologically unrelated individuals from MOZ and 291 biologically unrelated individuals from CAB who were utilized for downstream analyses.

3.10 Eight high-coverage whole genomes used for MSMC2

MSMC2 requires high-coverage whole genomes to reliably use[34]. We therefore sequenced an additional eight high-coverage whole genomes (4 collected from Cabinda, 2 Kongo, 1 Ovimbundu, 1 Kimbundu) and 4 collected from Maputo (2 Tsonga, 2 Makua) that were shipped to Novogene for 40X target 150bp paired-end, PCR-free whole genome sequencing using Illumina HiSeq X. To enable direct comparisons with genomes sequenced to similar coverage using the same technologies in the SGDP[35], individual-level FASTQ files for all 8 newly sequenced samples were processed according to the procedure outlined in Supplementary Section 1 ('Processing and Alignment') of Mallick et al.[35]. Specifically, paired-end reads were merged using SEQTK (URLs) *mergepe*. These reads were then mapped to the hs37d5 reference genome (URLs) using BWA mem[36] and sorted using samtools v1.8 *sort*[2] to generate sorted, individual-level BAM files. Autosomal coverage of these samples was approximately 37X as calculated using mosdepth. BAM files were processed and analysed for use with MSMC2[34]. We plan to extend analysis of these high-coverage whole genomes in future iterations of this work.

Supplementary Note 4. Merging with external data

4.1 Defining Sample Origin in newly sequenced dataset

Estimates for the number of Bantu languages across sub-Saharan Africa range from between 440 (Guthrie) and 680 often dividing regions both geographically and culturally. Ethnologue (URLs) notes 44 and 42 extant Bantu languages in Angola and Mozambique respectively, with many regional dialects only beginning to be described in detail[37]. To assign group labels to each study participant, we follow a stepwise process whereby individuals are described using (1) their dataset of origin (CAB, MOZ) (2) language/ethnic group (e.g., Kongo, Ovimbundu, Makua, Tsonga,

Chopi) based on self-reported primary language/dialect of both maternal and paternal parents and grandparents. Individuals with mixed ethnolinguistic groups (e.g., Kikongo + Kimbundu) were excluded from the HOA/Illumina dataset merging to aid in interpretability of geographic locations and fine-scale population structure across groups. Each individual's population label and language group in our merged dataset can be found in Supplementary Data 1, as well as their place-of-birth details and summarized in Table 1.

To improve power in our analyses (e.g., *f4* statistics, ROH/IBD, SOURCEFIND), we merged individuals from the genetically closely related Chopi and Tsonga ethnic groups into a single group (MOZ (south)), as these individuals show little apparent population structure or difference in genetic ancestry (see fineSTRUCTURE analyses, Supplementary Figure 6). However, we make no claims regarding the cultural homogeneity of these groups.

4.2 H3Africa-Baylor high-coverage whole genomes

333 high coverage 30X African whole genomes described in Choudhury et al.[18] as the H3Africa-Baylor data were downloaded from the European Genome Phenome Archive (EGA) after successfully applying for dataset access. Files were received in raw, per-chromosome, per-sample gVCF format. We genotyped and aggregated variant calls using the procedure outlined in Supplementary Note 3, whereby GATK4 GenomicsDBImport and GenotypeGVCFs were used to genotype all samples collectively to generate raw, unfiltered variant calls. After applying filters for genotype quality (GQ>=10) and depth of coverage (DP>=15) and missingness (F_MISSING<0.1) using bcftools *view*, we recovered 27,690,041 filtered autosomal SNPs, which were used to merge with additional datasets as described below (4.6/7). Individuals in this dataset were labelled as MAL, WGR, FNB, BRN, BSZ, BOT, CAM as specified in Choudhury et al.[18].

4.3 High-coverage ancient genomes

BAM files from three high-coverage ancient samples: a 2,000 year old individual from South Africa (Ballito Bay A; baa001)[38], a 4,500 year old individual from Ethiopia (Mota; GB20)[39], and an 8,000 year old individual from Cameroon (Shum Laka; I10871)[40] were downloaded, processed and subject to diploid variant calling using the procedure outlined in Schlebusch et al.[38], Supplementary Materials 4,"Defining Sample Origin in newly sequenced dataset". Specifically, for each BAM file, we set base quality scores (BQ) of Ts in the first 5bp of each read and As in the last 5bp of each read to 2. GATK v3.8[3] was used to realign INDELs with the 1000G callset used as a reference. GATK Unified Genotyper[3] was subsequently used to call diploid genotypes with the parameters -*stand_call_conf 50.0 –mbq 40 –contamination 0.02 -out_mode EMIT_ALL_SITES -gt_mode GENOTYPE GIVEN ALLELES* with variants within the dbSNP142 (URLs) given as known sites using the *–alleles* flag. Calls flagged as "Low Quality" were subsequently filtered out.

4.4 Chimpanzee reference genome

As a representative outgroup to all human populations, we use haploid genotypes from the Chimpanzee reference genome (URLs) aligned to the human reference hg19/GRCh37 (GRCh37). axtNet alignment files were downloaded from the UCSC (Chimp Human Alignment) and used to generate haploid VCF files.

4.5 Merging procedure

Throughout the study, we analyse our novel collection intermediate coverage samples in the context of autosomal genotype data generated by earlier studies. Each curated dataset is summarized in Supplementary Data 2 (WGS), Supplementary Data 3 (HOA), and Supplementary Data 4 (ILLUMINA) including given population labels and references. Usage of each merged dataset is dependent on the question of interest and specified in the main text, when necessary, alongside any further analysis-specific filtration of sites. For each dataset, prior to merging individuals across studies each REF/ALT allele was checked against the reference build hg19/GRCh37 using bcftools v1.9 norm –check-ref[17], flipped when necessary and subsequently annotated using a hexadecimal 64-bit VariantKey[41] encoding CHR/POS/REF/ALT to ensure maximum compatibility between datasets. A/T and C/G alleles were removed to mitigate strand ambiguity and SNPs at shared coordinates were merged using bcftools v1.9 merge. To reduce the effects of spurious genotype calls on downstream population genetic analyses, SNPs in difficult to sequence regions were filtered using a merged BED file (generated using bedtools v2.28.0[42] multiinter) including: (1) Our accessible genome mask (see Methods 5.3). (2) Heng Li's Low-Complexity Region (LCR) mask (URLs). (3) The hg19 ENCODE blacklist (URLs). (4) Segmental duplications from UCSC (URLs). We also filtered out one of every pair of duplicated or biologically related individuals (< 4th degree) using the KING-robust algorithm[32] implemented in PLINK2[33]. Each dataset was converted into the format required for the specific analysis in question, specifically VCF (IBDseq), EIGENSTRAT (*f4*, qpAdm, smartpca), PLINK bed/bim/fam (ROH), CHROMOPAINTER-specific hap/sample/ids format (CHROMOPAINTER) converted after phasing PLINK files using SHAPEITv2 as described in the main text. Genetic distances were set using the 1000G genetic map (URLs) for PLINK, EIGENSTRAT or CHROMOPAINTER.

4.6 Dataset descriptions

4.6.2 WGS dataset (to identify outlying individuals with recent European ancestry)

Prior to curating the WGS dataset described in the main text, we utilized a dataset specifically including all individuals 2,503 individuals from twenty-six populations in the 1000G, and all 320 individuals from six populations from the AGVP (AGVP; 4X WGS)[43] and all biologically unrelated, high-quality samples within CAB and MOZ (340 individuals, Data1). This dataset was used

specifically to identify EU ancestry among CAB and MOZ using ADMIXTURE[44] in an identical manner to that described for the HOA dataset in the main text (Methods 5), whilst additionally filtering for MAF < 0.05. We identified 25 individuals across CAB and MOZ with >5% European ancestry, likely very recently derived from contact with European migrants in Africa. These individuals were excluded from all population genetic analyses presented in the main text and to improve inference of population structure/admixture specific to events that occurred during or prior to the Bantu Expansion (e.g. European admixture may appear in fastGLOBETROTTER analyses and may influence clustering patterns on a PCA, making inference of fine-scale population structure difficult to interpret). These individuals and their European ancestry proportions are specifically detailed in Supplementary Data 1. We plan on performing a more comprehensive analysis of the nature of European mixture into sub-Saharan Africa in the future.

4.6.1 WGS dataset (main text)

To investigate population structure and relationships between our newly sequenced samples from CAB and MOZ datasets and other African populations with available WGS sequences, we used the above procedure to merge and filter genotypes from all individuals biologically unrelated, high-quality samples, without mixed familial ethnolinguistic groups, with less than <5% European ancestry (315 individuals, see Supplementary Data 1) with genotype data from African populations from 1000G, the African Genome Variation Project (AGVP; 4X WGS), and the H3Africa high-coverage whole genomes (H3A; 30X WGS). After removing multi-allelic variants, the 1000G (AFR, excluding the admixed ASW and ACB) dataset consisted of approximately 35 million autosomal, biallelic SNPs and 486 individuals from five populations (LWK, GWD, MSL, YRI, ESN), the AGVP consisted of approximately 33 million autosomal, biallelic SNPs and 320 individuals from six populations, and the H3A consisted of approximately 28 million autosomal, biallelic SNPs and 333 individuals from 7 (broadly defined) populations. We then combined this dataset with 14 individuals from 6 populations (Ju_hoan_North, Khomani, Mbuti, Biaka, Dinka) sequenced and genotyped as part of SGDP, selected based on the fact they are from populations (Nilo-Saharan, Khoe/San, rainforest hunter-gatherers) that represent potential analogues for groups known to have admixed with Bantu-speaking populations from previous research[43, 45] and three high-coverage ancient African whole genomes (baa001, GB20, I10871) processed and genotyped as described in Supplementary Note 4, “High-coverage ancient genomes”. The precise composition of individuals (e.g. a subset of individuals) and filtration of SNPs across this large WGS dataset depends on the specific analysis in question and as detailed in the relevant methods section in the main text. The chimpanzee reference genome was added specifically for calculation of *f4* statistics (Methods 7, Supplementary Table 2, Supplementary Fig. 3). All analysis of this dataset was performed as described in the Methods 6/7/8/9 of the main text.

4.6.2 HOA dataset

For further analysis including a wider range of sampled populations, we downloaded publicly available data from modern and ancient individuals genotyped at ~600k autosomal SNPs present on the Affymetrix Human Origins Array as part of the Allen Ancient DNA Resource (AADR) from the Reich Lab website (URLs), also genotyped in the Human Origins Array. We then filtered for only diploid individuals in this dataset, thus excluding ancient pseudo-haploid individuals and non-human genomes (e.g., Gorilla, Chimp, Macaque). The only non-diploid individuals that were kept were putative ancient Bantu-speaker related genomes as well as Levant_Neolithic, Iran_Neolithic, and South_Africa_1200BP ancient genomes used as reference groups or sources for qpAdm as described in Wang et al.[46]. Bantu associated individuals included: four South_Africa_400BP individuals from Schlebusch et al.[38], a single individual Tanzania_Pemba_700BP from Skoglund et al.[47], a single individual Kenya_IA_Deloraine from Prendergast et al.[48], and the genomes labelled Uganda_Musa_500BP, Botswana_Xaro_1400BP, Botswana_Taukome_1000BP, Congo_Kindoki_240BP from Wang et al.[46] (Botswana_Nqoma_900BP and Congo_Kindoki_150BP were excluded due to extremely high missingness in these samples (>98.5%)). Non-diploid genomes were only analyzed as part of the ADMIXTURE or qpAdm analysis performed on this dataset, and not on any downstream CHROMOPAINTER based analysis owing to the requirement for phased, diploid genotypes for use of this method. This HOA dataset was then merged with Niger-Congo speaking populations from the WGS dataset (Supplementary Data 2). Specifically, MAL, WGR, FNB, BRN, BSZ, BOT, CAM groups from the H3A dataset, Zulu and Baganda populations from the AGVP dataset, and 315 individuals among CAB and MOZ datasets that were sequenced as part of this study (and subset as described in Supplementary Data 1). 1000G AFR populations were not included as they were already represented among the AADR resource dataset. We also included the three high-coverage ancient African whole genomes (baa001, GB20, I10871) processed and genotyped as described in Supplementary Note 4, "High-coverage ancient genomes", as well as genotypes from high-coverage African genomes described in Fan et al.[49] downloaded after signed letter consent from the Simons Genome Diversity Project website (URLs). After merging, we filtered the diploid dataset (not including ancient pseudo-haploid genomes) for samples with missingness <5% after filtering for genotypes with missingness <5% (as required for imputation of missing genotypes using SHAPEITV2, a requirement for CHROMOPAINTER) resulting in a final set of 506,971 sites. All analysis of this dataset was performed as described in the Methods 5/10/11/12/13/14 of the main text. We stepwise SOURCEFIND analysis which we expand upon below (section 5).

4.6.2 ILLUMINA dataset

We additionally curated a dataset composed of genotype data from various studies (see Supplementary Data 4) largely genotyped using Illumina array technologies. These studies were selected to maximize the diversity of populations from across Africa in the dataset (including Bantu speaking groups) to aid in the ability to detect fine-scale population structure across CAB and MOZ. We first merged SNPs across all autosomes present in these studies as described above (4.5) and

additionally combined this dataset (as with the HOA) with Niger-Congo speaking populations from the WGS dataset. Specifically, MAL, WGR, FNB, BRN, BSZ, BOT, CAM groups from the H3A dataset, Zulu and Baganda populations from the AGVP dataset, and CAB and MOZ datasets that were sequenced as part of this study and present in the WGS dataset described in the main text. We then filtered the genotypes for those with <5% missingness across the sample set, resulting in a total of 276,024 filtered autosomal SNPs. This dataset was used specifically for the fineSTUCTURE analysis described in Methods 11.

Supplementary Note 5. A note on the stepwise SOURCEFIND and fastGLOBETROTTER analysis

Across many previous studies[18,43,45,50,51,52] work has been done to understand the timing and nature of migrations involving Bantu-speaking communities in sub-Saharan Africa and the subsequent admixture with local (or autochthonous) populations often represented by modern populations in South Africa (e.g. Khoe/San), Central Africa (e.g. the Aka rainforest hunter-gatherers) and Eastern Africa (e.g. Nilo-Saharan or Afro-Asiatic groups such as the Sudanese Dinka) as well as, more recently, with ancient genomes such as those from Shum Laka Cameroon[40] or Ballito Bay South Africa[38,46]. In this analysis, presented in Results 4 and Figure 2 in the main text, we aimed to gain insights into the timing of admixture events involving Bantu-speaking groups and possible dispersal patterns, with a particular focus on those related to migrations into and out of the regions surrounding present day Angola (as sampled in CAB) and Mozambique (as sampled in MOZ) from which our newly sequenced samples were collected.

5.1 SOURCEFIND and fastGLOBETROTTER

A combination of SOURCEFIND[53] and fastGLOBETROTTER[54] – which both leverage shared matrices of shared haplotypes between individuals in a dataset as estimated by CHROMOPAINTER[55] – has been shown to be a powerful means of identifying the closest matching mixture sources and dating and describing admixture across the past ~4,500 years respectively to gain insights into recent human migrations (for example, see Lopez et al.[56] for a recent example of this in a large dataset of present-day Ethiopians).

Specifically, SOURCEFIND is a sample-size correcting, Bayesian method that can be used to describe an individual (or group of individuals) as a convex combination of ancestry proportions most closely matched to a given selection of surrogate (source) populations and has been shown to outperform GLOBETROTTER alone at identifying true mixture sources and proportions[53]. However, as detailed in the SOURCEFINDv2 manual (URLs), the results of SOURCEFIND need not be related to admixture but, rather, is simply a measure of the relative amounts of shared recent ancestry between the target individual or population and the set of groups given as surrogates. As such, careful curation of and comparisons between surrogates and target population sets are

required to reasonably interpret the results of SOURCEFIND. For example, under and all vs all scenario, where all groups are given as both targets and surrogates, two neighbouring populations - X and Y - may be inferred by SOURCEFIND to derive 100% of their ancestry from each other (X = 100% Y and Y= 100% X) making inference about potential earlier routes of migration or shared admixture events difficult to ascertain.

5.3 A stepwise migration model of Bantu-migrations

To gain test support for migration routes taken by Bantu-speakers into and out of the regions surrounding present-day Angola (CAB) and Mozambique (MOZ) and to mitigate the masking of such information caused by the sharing of recent ancestry between closely related groups within our HOA dataset, we devised a stepwise analysis. Specifically, we start with the best fitting model of Bantu migrations described in Choudhury et al.[18] (Supplementary Fig.18), whereby (1) Cameroonian Bantu speakers (origin of the Bantu language group) first migrate to southwards through the rainforest the region surrounding present-day Angola (2) followed by eastward migration into Zambia (3). We also find support for such a model using IBD haplotypes calculated using WGS data (see Figure 1b) that extend this stepwise migration model by indicating the presence of serial founder events in the form CAB -> BSZ -> MOZ (north) -> MOZ (south). Explicitly: migration into Zambia in is then followed by migration that continues from Zambia to North Mozambique (4) followed by a southward migration into South Mozambique (5) (Supplementary Fig. 18).

Based on this model, we specifically curate the set of Bantu-speaking groups that are included as surrogates in each SOURCEFIND ancestry prediction step (1-5) using present-day groups surrounding the relevant geographic locations described above as proxies for the groups that migrated onwards from these locations, with each subsequent step including those groups that were included in the previous step. Explicitly for each step:

(1) We exclude all Bantu-speaking groups in our HOA dataset (see Supplementary Data 4 for details) other than Cameroonian Bantu and Bantoid groups H3A CAM, Lemande, Ngumba, Tikar South, Mbo, Aghem, Bafut, Bangwa as surrogates H3A CAM was separated into 4 clusters identified using fineSTRUCTURE (see Supplementary Figure 6a).
(2) We include CAB (Kongo), CAB (Kimbundu), and CAB (Ovimbundu) as possible surrogates in the model in addition to those in (1).
(3) We include H3A BSZ that cluster independently from Malawians (as estimated by fineSTRUCTURE and TVD, see section 5.4 below) as possible surrogates in the model in addition to those in (1) and (2).
(4) We include the Malawians genotyped in Skoglund et al.[47], MOZ (north) as possible surrogates in the model. fineSTRUCTURE shows that these newly sequenced MOZ individuals that speak north Mozambican languages cluster closely with these neighboring Malawian groups in addition to those in (1), (2), and (3).
(5) We include MOZ (south) as a potential surrogate in the model in addition to those in (1), (2), (3), and (4).

Using the selection of surrogates included in each step, we then run SOURCEFIND five times (once per step) as described in the main text (using the *HOA-all-copying model* chunk lengths matrix as input) to infer the proportion of DNA that each target individual shares with each given surrogate population. Target individuals include those individuals from all 92 African groups within the HOA dataset in steps ½ and 39 groups (including the 3 CAB groups) with evidence of Bantu-related ancestry from outside of Cameroon (see Supplementary Data 5) in steps 3/45, excluding those who were added as additional surrogates in the current step and all subsequent steps. For example, CAB (Kongo) individuals were targets in step 1, but not in steps 2,3,4 and 5, MOZ (north) were included as targets in steps 1,2,3 but not in steps 4 and 5 and the Zulu were included as targets in all steps. This is to avoid the proxy ancestral populations being described as a mixture of proxy derived populations by SOURCEFIND.

For each of these target groups we then run fastGLOBETROTTER as described in the main text (Methods 13) providing all groups (in addition to closely related autochthonous groups from the same broad ethnolinguistic group e.g., Nilotic, Khoe/San) that SOURCEFIND estimates as contributing 1% or more of their total DNA in the latest step in which the target was included. For example, the AGVP Baganda could best be described in step 5 as a mixture of Tumbuka (39%), Lemande (11%), Sengwer (19%), CAB (Kongo) (9%), Iraqw (2%) and Mbuti (1%), BSZ* (1%), Tikar_South (1%). We therefore provided Tumbuka, Lemande, CAB (Kongo), Tikar_South, Chewa and all Nilotic, Afro-Asiatic, and rainforest hunter-gatherer groups as possible surrogates for fastGLOBETROTTER to estimate the timing and nature of admixture events in this target group. CAB groups were an exception to this. Here, many individuals showed on average <1% hunter-gatherer ancestry from a single source group. However, a smaller subset of individuals showed >1% rainforest hunter-gatherer related ancestries collectively (and also show evidence for HG ancestries in the no Bantu copying model), so we included all rainforest hunter-gatherers and the Lemande and groups among CAM as input to fastGLOBETROTTER Results of this stepwise SOURCEFIND and fastGLOBETROTTER analysis, including the specific surrogate populations, dates of admixture, and other results from fastGLOBETROTTER (e.g., single or multiple date admixture) that best describe the admixture event in each target group can be found in Supplementary Table 4.

5.4 Bantu-speakers from Southern Zambia (BSZ) as an intermediate group

Whilst the model presented in Choudhury et al.[18], as well as our own analysis of IBD haplotypes (Figure 1c in main text) suggest the sequenced BSZ group from Southern Zambia to be a good proxy for the intermediate splitting population between Angolans and Eastern Bantu speakers, we note that the model in Choudhury et al.[18] did not include any neighboring groups from Malawi or Mozambique and also note that this group contains multiple ethnolinguistic groups corresponding to Chikund/Soli (south-east), Tumbuka (north-east) and Bemba (central). As such, especially considering the linguistic overlap between some individuals labelled as BSZ in Choudhury et al.[18] and

Malawians included in our HOA dataset (e.g. Tumbuka speakers), we did not simply use all individuals in BSZ as an intermediate group between CAB groups and Malawians or MOZ (north).

Instead, we start from the results of the fineSTRUCTURE analysis performed using the HOA *all-copying model* chunk counts matrix. This analysis shows that a the BSZ group contains substructure (see Supplementary Data 5). Here, we identify three groups: (1) individuals within BSZ that cluster independently from Malawians, and (2/3) groups of individuals within BSZ that variably cluster alongside Malawian groups. We suspect that the BSZ (2/3) set of individuals should not be used as an intermediate group between CAB and Malawians or north Mozambicans (who also cluster closely with Malawians according to fineSTRUCTURE) in the stepwise analysis described above, and are instead more closely related to these two later groups – aligned with the considerable linguistic overlap between these neighboring countries. However, the BSZ (1) group may be a better proxy for this. Owing to lack of precise ethnolinguistic information related to individuals among BSZ, we therefore did not decide to use BSZ (2/3) groups sources in this model but note that ancestries among these groups are very similar to Malawians and that any ancestry matched to Malawians are likely to also be matched to those from eastern Zambia (if not closer in some instances).

To test that BSZ (1) group is a better proxy for an intermediate group between CAB and Malawians or north Mozambicans, we calculated Total Variation Distance (*TVD*) for all pairs of modern Bantu-speaking individuals in our HOA dataset based on the chunk lengths matrices generated as part of the HOA *all-copying model*. Starting with our "chunklengths" copying matrices which describe the lengths of autosomal DNA which individuals *(i, j)* are inferred to share a MRCA with K distinct given donor populations, that is, the lengths of DNA from each population (*k*) which CHROMOPAINTERv2 has used to "paint" an individual, we calculate:

$$TVD_{i,j} = 0.5 \sum_{k=1}^{K} |f_i^k - f_j^k| \quad (1)$$

where f^k_j is the total proportion of autosomal DNA that individual *i* is inferred to match to individuals from donor group k. As in Lopez et al.[56] we estimate 1- TVD_{ij} as a measure of genetic similarity, averaging this score across all pairs of individuals (*i,j*) when reporting this measure within or between groups. Average pairwise *1-TVD* between populations can be seen in Supplementary Figure 19.

We then use a simple permutation test to test the hypothesis that the BSZ (1) groups are intermediate between CAB groups and Malawian or MOZ (north) groups. Under this assumption, we should expect BSZ (1) to be genetically closer to CAB groups relative to Malawians and MOZ (north) groups (owing to reduced differentiation from CAB) whilst being genetically closer to Malawians or MOZ (north) relative to CAB (owing to the fact Malawians and Mozambicans are expected to be derived from populations closer to BSZ (1) in our stepwise model).

Specifically, we take the two vectors of pairwise *1-TVD* values $TVD_{A,B}$ and $TVD_{A,C}$ of length *n* and *m* for populations A, B, and C and calculate:

$$d0 = mean(TVD_{A,B}) - mean(TVD_{A,C}) \quad (2)$$

To test the null hypothesis that population B is genetically closer to population A than population C is to population A, we resample two vectors $TVD_{A,X}$ and $TVD_{A,Y}$ of length *m* and *n* from the combined vector of ($TVD_{A,B}$, $TVD_{A,C}$), calculate:

$$d = mean(TVD_{A,X}) - mean(TVD_{A,X}) \quad (3)$$

and take the proportion of instances of 9,999 permutations of this approach where $d > d0$ as our one-sided p-value.

From this, we find that BSZ (1) is closer genetically to CAB (Kongo), CAB (Kimbundu), and CAB (Ovimbundu) (mean $1\text{-}TVD_{CAB,BSZ} = 0.82$) than all Malawians and MOZ (north) (mean $1\text{-}TVD_{CAB,Malawi} = 0.74$) (permutation test $p < 0.0001$). We also find that BSZ (1) is closer genetically to Malawians and MOZ (north) (mean $1\text{-}TVD_{BSZ,Malawi} = 0.88$) than all CAB groups (permutation test $p < 0.00001$). Similar findings are apparent indicating Tumbuka/BSZ (2) as being a closer intermediate to Ugandans/Kenyans and Makua being a closer intermediate to Chopi/Tsonga or South Africans ($p < 0.0001$).

Finally, we test whether BSZ (1) can be described as an admixture of Malawians or MOZ and CAB (which could lead to them appearing as an intermediate group) using fastGLOBETROTTER providing all possible donor populations as surrogates and using the HOA *all-copying-model* chunk length file as input. We find bootstrap resampled dates of admixture equal to 1 when performing this analysis which we take as little or no evidence for admixture. However, we note the small sample size of BSZ (1) and the close relatedness between CAB, MOZ, and Malawians makes admixture difficult to detect, and that further analysis and wider sampling of Zambia is required to rule this possibility out. Here though, we utilize the model described in Choudhury et al.[18] and take BSZ (1) as an intermediate group between Angolans (CAB) and Malawians in the succession of Bantu migrations into South-Eastern Africa for the model presented in the main text. We use a general location in the center of Zambia to represent this group in Figure 2a, owing to lack of precise sampling location/ethnolinguistic affiliations.

5.5 Alternative models where MOZ(north)/Malawians are derived from migrations out of Uganda or MOZ (south) are derived from migrations out of Botswana appear less parsimonious

Distinct model of migration may also be plausible, for example ones in which Western Bantu speakers first migrated east to the Great Lakes region prior to dispersals into the regions surrounding Malawi Mozambique to the south, as opposed to the best fitting model of Choudhury et al.[18] where the Ugandan Bantu-speaking groups were best modelled as being derived from migrations out of the region surrounding Zambia (represented by BSZ). We note that in the stepwise model

presented in Figure 2, the Ugandan Baganda are shown to match additional ancestry to the Cameroonian Lemande group in step 3 specifically when BSZ (1) are added and in all subsequent steps, which may suggests that ancestry in this group represents an earlier branching dispersal into East Africa from regions such as the southern Democratic Republic of the Congo (rejected by Choudhury et al.[18]) which are not well represented in our model of Bantu-speaker dispersals.

Notably, adding single individuals (Lunda, Chokwe, Nngala, Luba) from further east of Angola and the DRC present within CAB (see Supplementary Data 1) in step does mitigate this signal of matching to the Lemande relative to BSZ (1), with the Baganda (previously represented as 64% BSZ(1), 11% Lemande, 20% Sengwer, 1% Mbuti, 1% Tikar (South), 1% Kimbundu, 1% Kongo, 1% Iraqw in the step 3 of the model described above instead represented as 49% BSZ (1), 20% Sengwer, 18% Lunda, 5% Chokwe 1% Ngala, 1% Mbuti, 2% Iraqw. Similar patterns of continued shared ancestry to CAB groups are apparent among groups from north-east of the Kalahari in the northernmost parts of Botswana (Shua, Tshwa) (who instead retain ancestry to the Ovimbundu peoples) in addition to BSZ (1). Taken together these models may indicate a more diffuse eastward migration through Zambia and broader parts of central Africa into the Great Lakes region and northern Botswana branched before further migrations into Malawi, Mozambique, South Africa and southern Botswana (or multi-wave dispersal with admixture).

To test whether Uganda represented a good proxy for an intermediate between Angolans and Malawians or MOZ (north), we performed a second stepwise SOURCEFIND analysis replacing BSZ (1) with the Ugandan Baganda in step 2 of the model and instead moving BSZ to step 3 alongside Malawian groups, which, if reasonable, could be indicative of a migration that primarily occurred from the Great Lakes region (first) and then into Malawi/Mozambique rather than a primary migration through the region surrounding Zambia (Supplementary Data 5, step 3 alt). However, here, BSZ (1), Malawians and Makua peoples retain significant proportions of their ancestry (between 64% in BSZ (1) and 50% in Mozambicans and Malawians) to the CAB Kimbundu relative to the Baganda. We thus further tested whether, instead, Malawians and north Mozambicans could be described as an admixture of the Baganda and groups from CAB. However, we find no evidence for this, with increasing coancestry curves indicating Baganda and CAB are from the same source population (data not shown). We therefore suggest this model of migration directly south from the Great Lakes region is less parsimonious than that presented in Figure 2 in the main text.

The model presented in Choudhury et al.[18] also indicated the possibility of direct migration from Zambia to Botswana. In that case, it is possible that MOZ (south) could be better represented by Botswanans as an intermediate than MOZ (north). We therefore also tested whether MOZ (south) could equally be well explained as being derived from groups from Botswana by including only Botswanans (BOT, Kgalagadi, Tswana) in step 4 (Supplementary Data 5, step 4 (alt)) instead of adding Malawians and MOZ (north) to the model. Here, we find MOZ (south) retains between 36-56% of their ancestry to the intermediate population BSZ (1) relative to Kgalagadi, Tswana, or BOT, similarly suggesting that MOZ (south) was earlier branching than these Botswanan groups (although we note that BOT is a heterogeneous group which may influence these results). These

patterns, in addition to. the earlier dates of admixture in MOZ (south) around 1,300 BP relative to those in Botswana of around 550-650 BP also supports a model wherein MOZ (south) should be added into the model prior to Botswanan groups as more parsimonious. However, we note exceptions below.

5.6 Heterogeneity among Bantu-speakers from Botswana (BOT) from H3Africa-Baylor

As noted in Choudhury et al.[18], BOT are a meta-group sampled from across Botswana and, therefore, are likely to have somewhat heterogeneous ancestry. As we were unable to get access to the precise ethno-linguistic affiliations of these individuals, we first explored whether this group showed variation in ancestry matching to different Bantu-speaking source groups using our SOURCEFIND analysis (step 5 above). As Botswana as a nation span both sides of the Kalahari - which we infer largely separates groups that share ancestry closer to CAB (Ovimbundu) than MOZ (south) (Figure 2a) - not having precise distribution of sampled ethnolinguistic group origins leads to difficulty in estimating whether any such heterogeneity is mediated by geography as appears to be the case for many of the other groups surrounding the Kalahari Desert.

In Supplementary Figure 9, we show that BOT shows some degree of heterogeneity in whether ancestry is most closely matched to CAB, Malawians, or MOZ (south) or Khoe/San groups – likely due to variation in such ancestry appearing across various Botswanan ethnolinguistic groups from different parts of the country. We therefore excluded this group from Figure 2a, as any inference of dispersal patterns may be incorrectly interpreted.

Supplementary Tables

Supplementary Table 1. Total *f2* doubletons shared between CAB or MOZ and populations from the 1000G and the AGVP (randomly subsampling 40 individuals per group; Supplementary Data 2). * Includes individuals whose country-of-origin was in Ethiopia as collected in the AGVP.

Dataset ID	Population ID	CAB *f2*	MOZ *f2*
Study	CAB	0.203	0.187
Study	MOZ	0.174	0.192
AGVP	Zulu	0.071	0.206
AGVP	Baganda	0.092	0.078
AGVP	Ethiopia*	0.031	0.036
1000G	Mandenka (GWD)	0.029	0.019
1000G	Mende (MSL)	0.037	0.024
1000G	Yoruba (YRI)	0.044	0.027
1000G	Esan (ESN)	0.052	0.032
1000G	Luhya (LWK)	0.093	0.083
1000G	African Americans (ASW)	0.052	0.039
1000G	African-Carribeans (ACB)	0.064	0.031
1000G	Toscani (TSI)	0.002	0.002
1000G	Finnish (FIN)	0.001	0.001
1000G	British (GBR)	0.001	0.001
1000G	CEPH/Utah Residents (CEU)	0.002	0.001
1000G	Iberian (IBS)	0.002	0.002
1000G	Han Beijing (CHB)	0.002	0.002
1000G	Southern Han (CHS)	0.002	0.002
1000G	Dai (CDX)	0.002	0.002
1000G	Kihn (KHV)	0.002	0.002
1000G	Japanese (JPT)	0.002	0.002
1000G	Mexican (MXL)	0.005	0.004
1000G	Puerto Rican (PUR)	0.013	0.008
1000G	Colombians (CLM)	0.007	0.005
1000G	Peruvians (PEL)	0.005	0.004
1000G	Gujarati (GIH)	0.002	0.002
1000G	Punjabi (PJL)	0.002	0.002
1000G	Bengali (BEB)	0.002	0.002
1000G	Tamil (STU)	0.002	0.002
1000G	Telugu (ITU)	0.002	0.002

Supplementary Table 2. *f4* statistics in the form *f4*(MOZ (south), MOZ (north); X, Chimp) where X is any Niger-Congo groups with n>10 from CAB, the AGVP, the 1000G, or H3A (Supplementary Data 2). Each population was subsampled to 10 individuals to avoid sample size influencing Z scores/fstats. Positive scores denote increased allele sharing with MOZ (south) and X relative to the proposed 4 population phylogeny. Red denotes statistically significant (Z > 3) and orange denotes almost significant Z scores (Z ~ 3).

X	*f4*	standard error	Z score
CAB	1.28E-04	8.00E-05	1.608
Zulu	0.000331	7.50E-05	4.393
Baganda	9.70E-05	8.00E-05	1.216
GWD	0.000117	7.90E-05	1.471
MSL	6.00E-05	7.60E-05	0.791
YRI	9.50E-05	8.00E-05	1.187
ESN	0.00014	8.20E-05	1.7
LWK	0.000105	7.80E-05	1.334
MAL	5.40E-05	7.90E-05	0.684
WGR	3.90E-05	8.00E-05	0.491
FNB	0.000119	7.90E-05	1.517
BRN	0.000129	8.10E-05	1.587
CAM	0.000106	7.70E-05	1.367
BSZ	0.000106	7.90E-05	1.341
BOT	0.000221	8.10E-05	2.956

Supplementary Table 3. Mean cumulative pairwise IBD (2-4cM) inferred using IBDseq vs. geographic distance from Cabinda.

Population ID	Mean pairwise cumulative within population IBD 2-4 (cM)	Standard Deviation (cM)	Approximate Distance from Cabinda (km)
CAB	4.6	0.6	0
BSZ	11.7	1.4	1950
BOT	19.2	2.6	2500
Baganda	15.1	2.4	2600
LWK	16.1	1.3	2850
MOZ (north)	16	2.5	3050
MOZ (south)	20.8	2.3	3150
Zulu	25.7	1.3	3225

Supplementary Table 4. qpAdm two-way admixture models testing ancestry among ancient Bantu speaker associated genomes. Reference groups and ancient southern African hunter-gather related source populations were defined as per Wang et al.[46]. Models with $p < 0.05$ and those with infeasible model fits (negative ancestry proportions) are highlighted in red. Increased Khoe/San related admixture among present day Tswana and Kgalagadi from Botswana is almost certainly the cause of a lack of model fit among these ancient genomes. Prop., Proportion.

Target	p-value	Source 1	Source 2	Prop. (1)	Prop. (2)
Botswana_Taukome_1100BP	0.2600	SA_Ovambo	Ballito Bay A	0.879	0.121
Botswana_Taukome_1100BP	0.3245	BSZ	Ballito Bay A	0.885	0.115
Botswana_Taukome_1100BP	0.3852	MOZ (north)	Ballito Bay A	0.888	0.112
Botswana_Taukome_1100BP	0.1830	MOZ (south)	Ballito Bay A	0.894	0.106
Botswana_Taukome_1100BP	0.0013	Kgalagadi	Ballito Bay A	1.347	-0.347
Botswana_Taukome_1100BP	0.4272	Tswana	Ballito Bay A	1.125	-0.125

Botswana_Xaro_1400BP (XAR001)	0.4427	SA_Ovambo	S_Africa_1200BP	0.688	0.312
Botswana_Xaro_1400BP (XAR001)	0.1262	BSZ	S_Africa_1200BP	0.686	0.314
Botswana_Xaro_1400BP (XAR001)	0.1034	MOZ (north)	S_Africa_1200BP	0.693	0.307
Botswana_Xaro_1400BP (XAR001)	0.1411	MOZ (south)	S_Africa_1200BP	0.691	0.309
Botswana_Xaro_1400BP (XAR001)	9.14E-19	Kgalagadi	S_Africa_1200BP	1.265	-0.265
Botswana_Xaro_1400BP (XAR001)	3.49E-06	Tswana	S_Africa_1200BP	0.955	0.045

Botswana_Xaro_1400BP (XAR002)	0.6631	SA_Ovambo	S_Africa_1200BP	0.602	0.398
Botswana_Xaro_1400BP (XAR002)	0.6834	BSZ	S_Africa_1200BP	0.606	0.394
Botswana_Xaro_1400BP (XAR002)	0.5713	MOZ (north)	S_Africa_1200BP	0.611	0.389
Botswana_Xaro_1400BP (XAR002)	0.5686	MOZ (south)	S_Africa_1200BP	0.612	0.388
Botswana_Xaro_1400BP (XAR002)	1.25E-13	Kgalagadi	S_Africa_1200BP	1.075	-0.075
Botswana_Xaro_1400BP (XAR002)	0.0009	Tswana	S_Africa_1200BP	0.829	0.171

South_Africa_400BP	0.0497	SA_Ovambo	Ballito Bay A	0.906	0.094
South_Africa_400BP	0.5530	BSZ	Ballito Bay A	0.901	0.099
South_Africa_400BP	0.3083	MOZ (north)	Ballito Bay A	0.909	0.091
South_Africa_400BP	0.3129	MOZ (south)	Ballito Bay A	0.912	0.088
South_Africa_400BP	0.1604	Kgalagadi	Ballito Bay A	1.373	-0.373
South_Africa_400BP	0.8740	Tswana	Ballito Bay A	1.139	-0.139

Supplementary Table 5. Intra- and inter-population statistics calculated for ABC analysis.

Statistic	Intra-Population	Inter-Population	Software	Command	Reference
No. Segregating Sites (S)	Yes	No	scikit-allel	count_segregating()	https://scikit-allel.readthedocs.io/en/stable/
No. Singletons	Yes	No	""	count_singletons()	https://scikit-allel.readthedocs.io/en/stable/
Pi	Yes	No	""	sequence_diversity()	https://scikit-allel.readthedocs.io/en/stable/
Tajima's D	Yes	No	""	tajima_d()	https://scikit-allel.readthedocs.io/en/stable/
Mean Heterozygosity	Yes	No	""	heterozygosity_observed()	https://scikit-allel.readthedocs.io/en/stable/
Variance Heterozygosity	Yes	No	""	heterozygosity_observed()	https://scikit-allel.readthedocs.io/en/stable/
Number of *f2* Alleles	Yes	Yes	""	count_doubletons()	https://scikit-allel.readthedocs.io/en/stable/
Hudson's F_{ST}	No	Yes	""	average_hudson_fst()	https://scikit-allel.readthedocs.io/en/stable/
Mean Number of ROH	Yes	No	PLINK	PLINK --homozyg -homozyg-kb 800	Purcell et al.[32]
Mean Cumulative ROH	Yes	No	""	""	Purcell et al.[32]
Variance Cumulative ROH	Yes	No	""	""	Purcell et al.[32]
Mean Number of IBD (>2cM)	Yes	Yes	GERMLINE	GERMLINE -bits 128 -haploid -h_extend err_hom 1	Gusev et al.[57]
Mean Cumulative IBD (>2cM)	Yes	Yes	""	""	Gusev et al.[57]
Variance Cumulative IBD (>2cM)	Yes	Yes	""	""	Gusev et al.[57]
Median Cumulative IBD (>2cM)	Yes	Yes	""	""	Gusev et al.[57]
Cumulative IBD 10% Quantile (>2cM)	Yes	Yes	""	""	Gusev et al.[57]
Cumulative IBD 25% Quantile (>2cM)	Yes	Yes	""	""	Gusev et al.[57]
Cumulative IBD 75% Quantile (>2cM)	Yes	Yes	""	""	Gusev et al.[57]
Cumulative IBD 90% Quantile (>2cM)	Yes	Yes	""	""	Gusev et al.[57]

Supplementary Table 6. Selection of parameters and associated prior distributions used to simulate SNP data under a two population split models using msprime for describing a clean split scenario.

Parameter Name	Description	Scenario	Prior bounds [low,high]	Distribution
NCAB	Ne of Mozambique at Gen. Present	a,b	[3,5]	log10 uniform
NMOZ	Ne of Angola at Gen. Present	a,b	[3,5]	log10 uniform
Gen. Split	Generation at which Mass Migration occurs to form single ancestral meta-population	a,b	[10, 1000]	uniform
N'CAB	Ne of CAB at Gen. Split	a,b	[4,6]	log10 uniform
N'MOZ	Ne of MOZ at Gen. Split	a,b	[4,6]	log10 uniform

Supplementary Table 7. Posterior parameter estimates for CAB and MOZ using the abc neuralnet function alongside training data generated by calculating summary statistics on 135,000 simulations of clean split model. Model evaluation was performed using 1,000 pseudo-observed simulations. MAE, Median Absolute Error; MSE, Median Squared Error; RMSE, Root Median Squared Error.

Parameter Name	Posterior Estimate (Median)	95% CI	MAE	MSE	RMSE	% of pseudo observed inside 95% CI
NCAB	6.4	[6.08, 6.7]	0.07	0.01	0.13	96.5
NMOZ	5.6	[5.1, 6.1]	0.07	0.01	0.11	97.5
Gen. Split	118.2	[100,5, 136.8]	23.3	1195.2	34.7	97.2
N'CAB	4.3	[4.1,4.4]	0.05	0.007	0.09	93.1
N'MOZ	3.5	[3.1,3.7]	0.05	0.005	0.07	94.1

Supplementary Figures

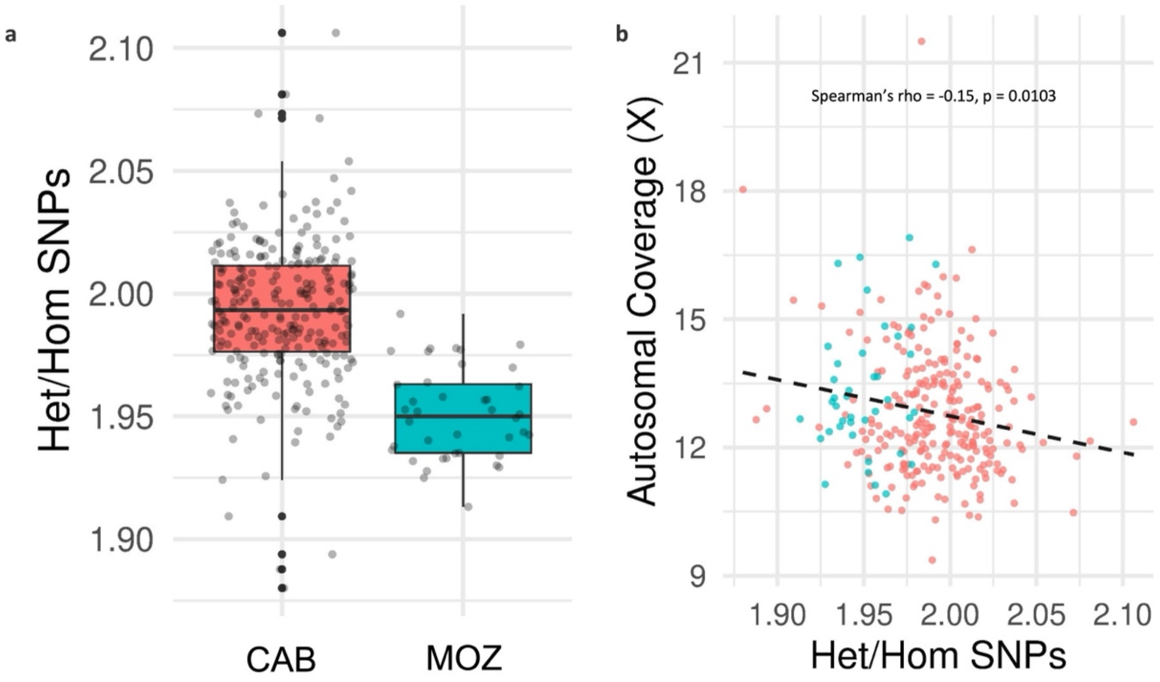

Supplementary Figure 1. **(a)** Boxplot showing ratio of heterozygous (Het) to homozygous (Hom) autosomal SNPs across n = 271 biologically independent individuals from CAB and n = 41 biologically independent samples from MOZ. Individuals with significant (>1%) European ancestry were excluded as admixture is known to increase the heterozygosity ratio (Supplementary Table 1). For each of CAB and MOZ, centre lines show median values (Q2, CAB = 1.991, MOZ = 1.951), with limits showing 25th (Q1, CAB = 1.976, MOZ = 1.935) and 75th (Q3, CAB = 2.011, MOZ = 1.963) quartiles. Whiskers above and below the box show the maximum (CAB = 2.053, MOZ = 1.991) and minimum (CAB = 1.924, MOZ = 1.913) values within 1.5 times the interquartile range (IQR) above or below Q3 or Q1 respectively. Points show outlier values more extreme than these minimum and maximum values. **(b)** Modest negative correlation between Het/Hom ratio and autosomal SNPs, indicative of increased error rate among those sequenced to a lower depth of coverage. The dashed line shows regression line calculated using a linear model with formula y~x.

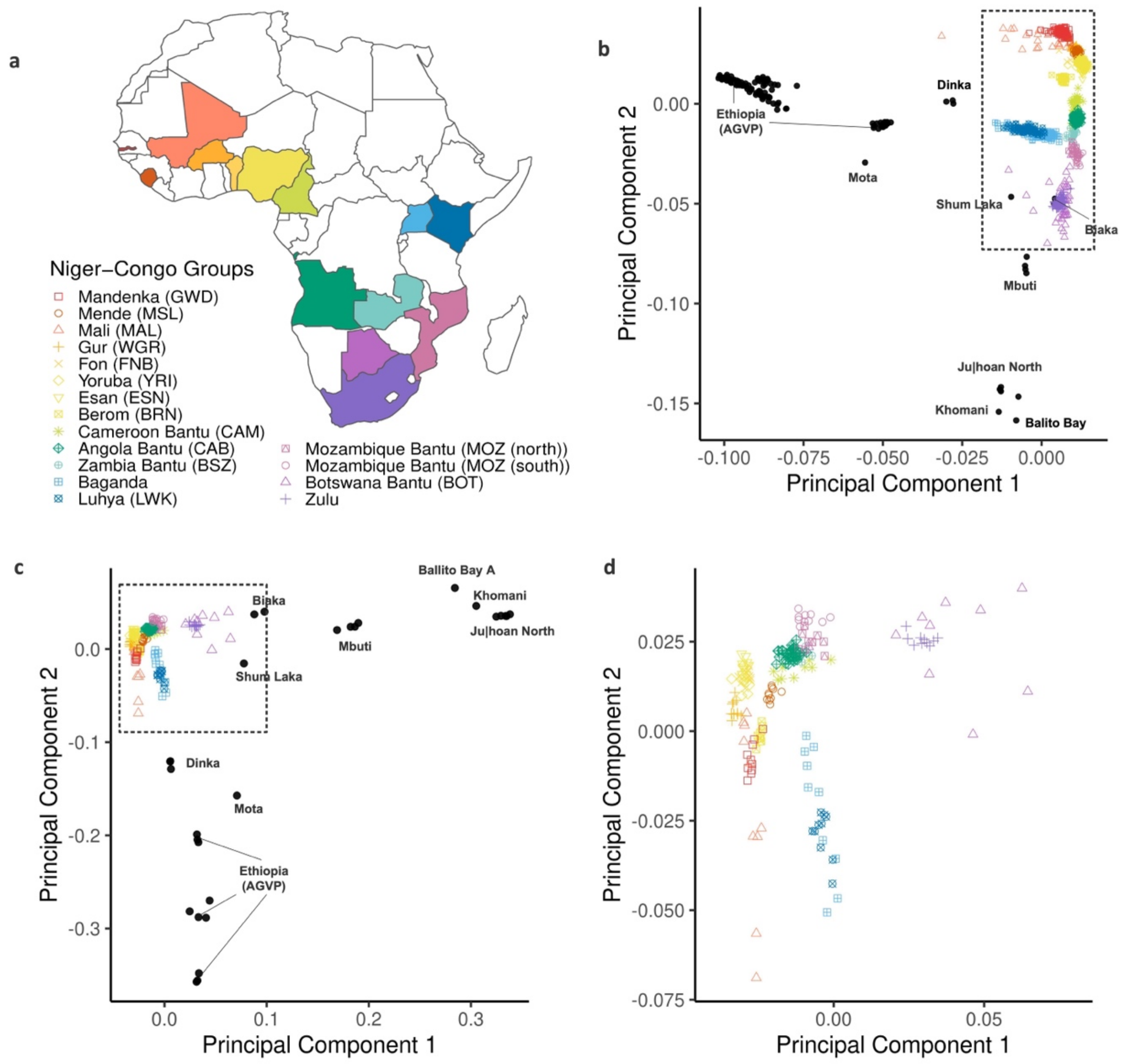

Supplementary Figure 2. (a) Map of Africa showing country of origin and legend for each group. Map made with Natural Earth. Free vector and raster map data @ naturalearthdata.com. **(b)** Full Principal Components Analysis (PCA) with eigenvectors (PC1 and PC2) constructed using modern African populations from the merged WGS dataset (Supplementary Table 2), ancient African genomes (Mota, Shum Laka, Ballito Bay) were projected onto these axes. Highlighted area shows zoomed in PCA shown in Figure 1b of the main text. Population structure follows clines primarily in East African (Dinka, Ethiopia, Mota) related ancestry and Khoe/San (Ju|hoan North, Khomani, Ballito Bay) related ancestry as shown by analysis using *f4* statistics (Supplementary Figure 2). **(c)** As in (a) but groups all Niger Congo groups and AGVP Ethiopians were down sampled to 10 individuals. Dotted line shows zoomed in PCA in (d). **(d)** Zoomed in PCA showing only Niger Congo speakers from (c).

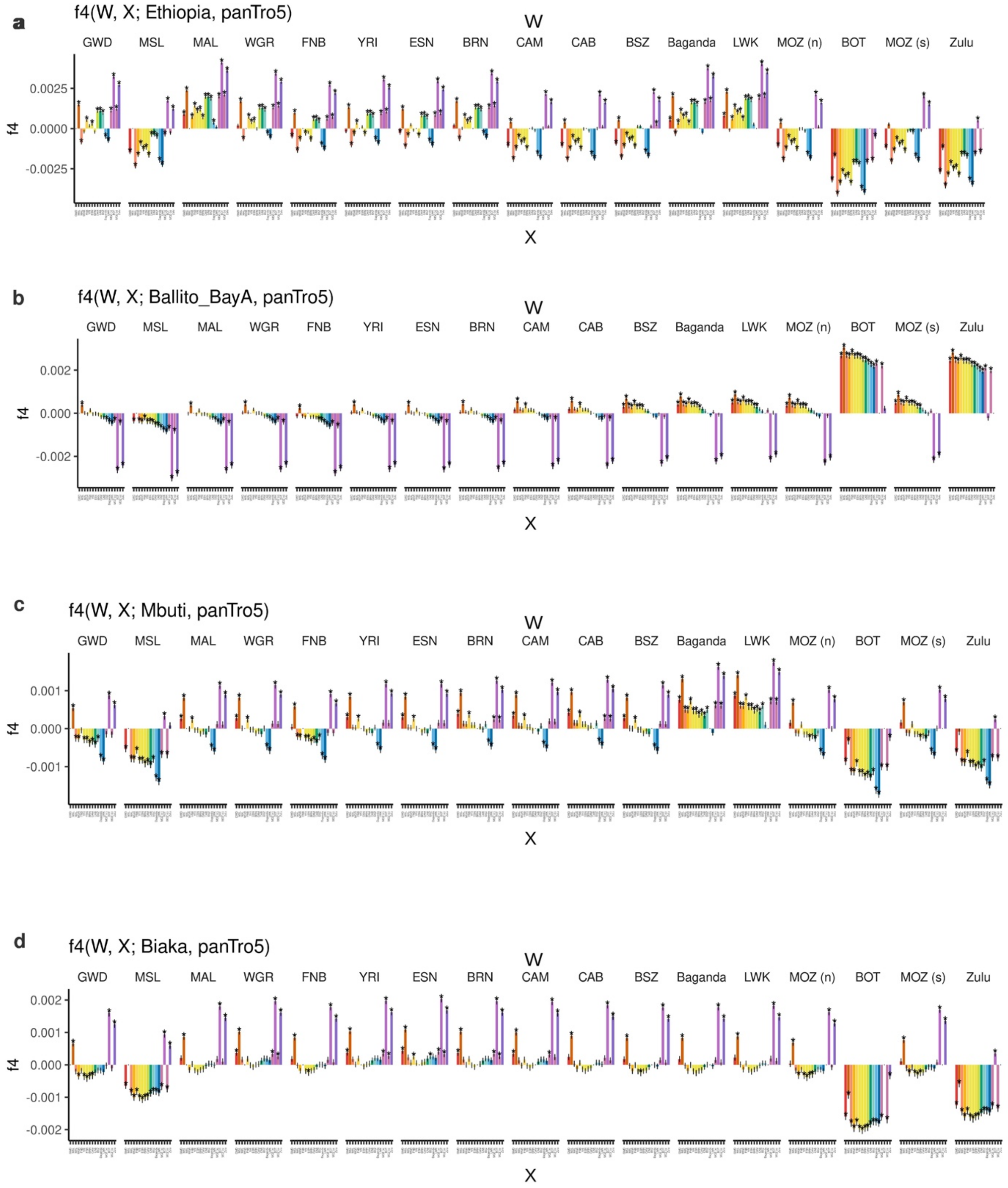

Supplementary Figure 3. *f4* statistics in the form *f4(W, X; Y, panTro5)* comparing relative allele sharing between pairs of Niger-Congo speaking populations (*W, X*) and other modern and ancient African genomes (*Y*) **(a)** Ethiopia (AGVP) **(b)** Ballito Bay A **(c)** Mbuti **(d)** Biaka. Each group (*W, X*) were down sampled to n = 10 biologically independent individuals. * indicates significant *f4*

statistics ($Z < -3$ or $Z > 3$). Error bars show standard errors (+/- SE) of *f4* statistics. Z-scores and standard errors were estimated using weighted block-jacknife over segments of 5-centimorgans (cM).

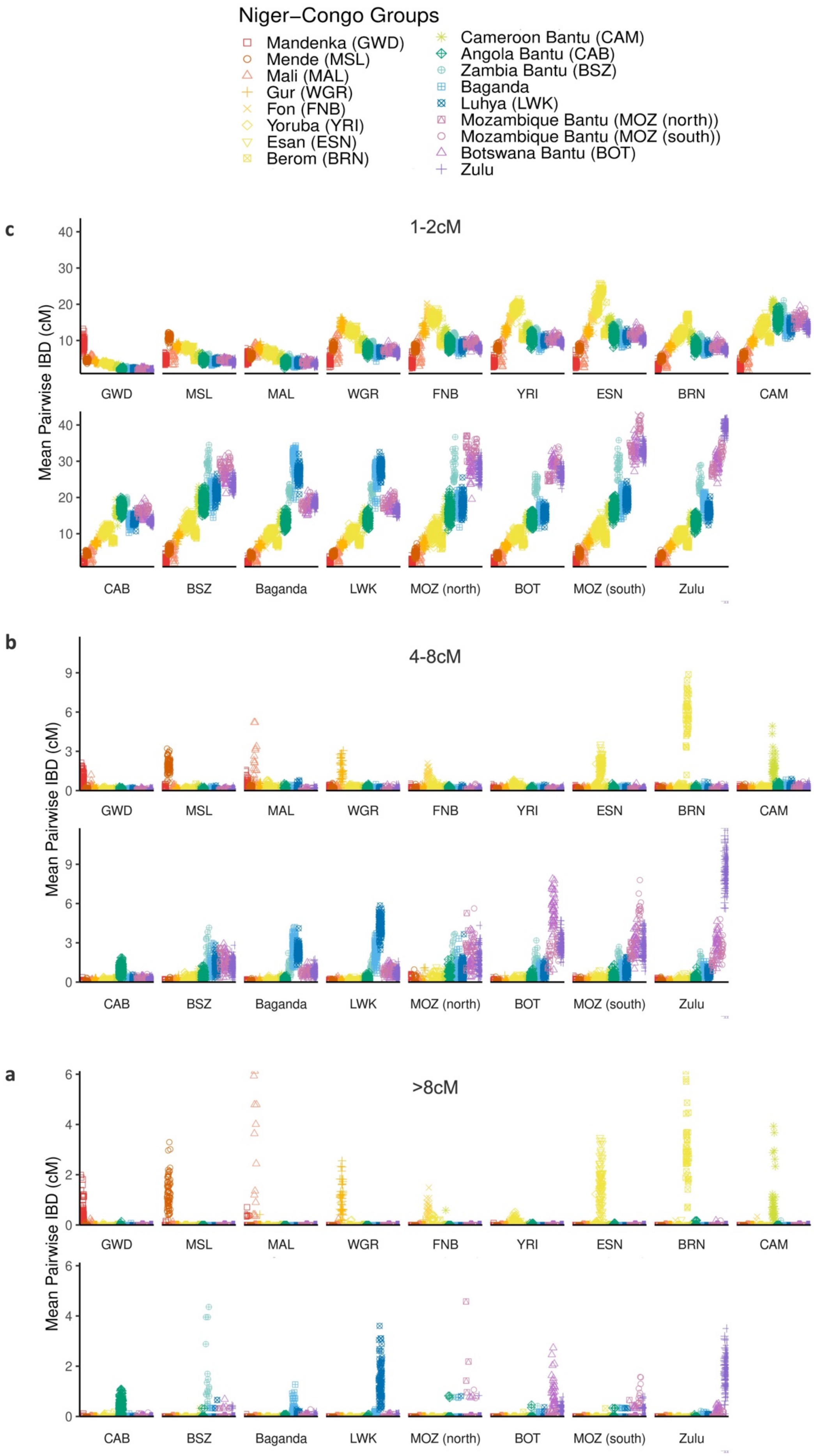
Niger–Congo Groups
Mandenka (GWD)
Mende (MSL)
Mali (MAL)
Gur (WGR)
Fon (FNB)
Yoruba (YRI)
Esan (ESN)
Berom (BRN)
Cameroon Bantu (CAM)
Angola Bantu (CAB)
Zambia Bantu (BSZ)
Baganda
Luhya (LWK)
Mozambique Bantu (MOZ (north))
Mozambique Bantu (MOZ (south))
Botswana Bantu (BOT)
Zulu
c
1-2cM
b
4-8cM
a
>8cM
Mean Pairwise IBD (cM)
GWD
MSL
MAL
WGR
FNB
YRI
ESN
BRN
CAM
CAB
BSZ
Baganda
LWK
MOZ (north)
BOT
MOZ (south)
Zulu

Supplementary Figure 4. Average cumulative IBD that each individual shares with another individual from each population the merged WGS dataset (Supplementary Table 3), specifically for Niger Congo speaking populations. **(a)** >8cM IBD **(b)** 4-8cM IBD **(c)** 1-2cM IBD.

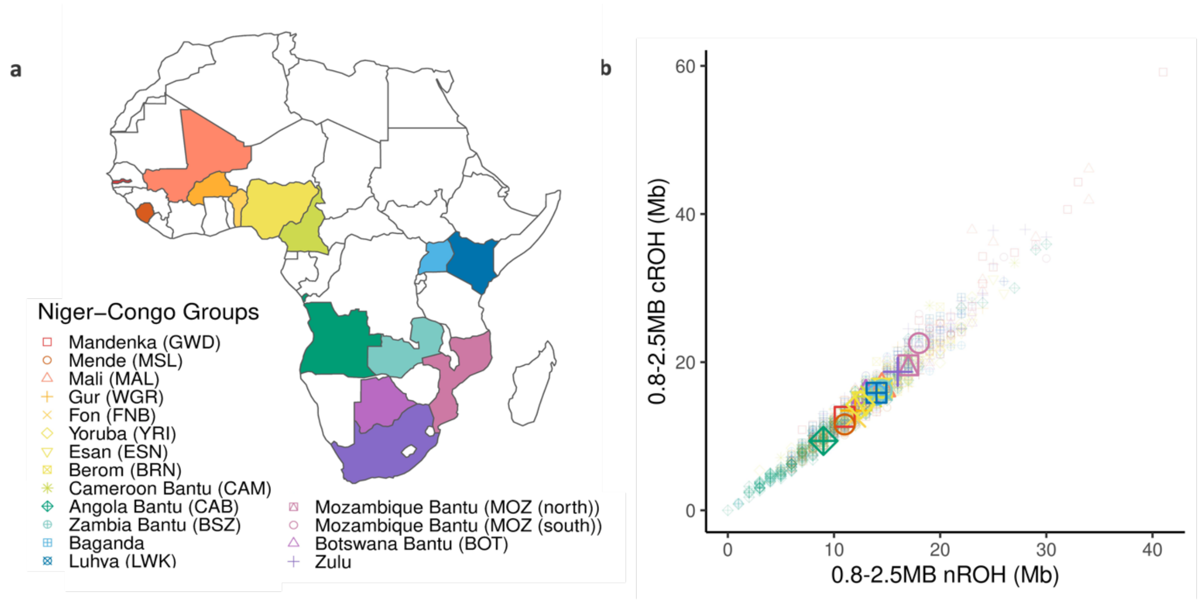

Supplementary Figure 5. (a) Legend denoting colour and shape of points corresponding to an individual from Niger-Congo populations present in our merged WGS dataset (Supplementary Table 3). Map made with Natural Earth. Free vector and raster map data @ naturalearthdata.com. **(b)** Average number of short Runs-Of-Homozygosity (nROH) and cumulative short ROH (cROH) per population in the merged dataset. It should be noted that significant recent admixture with autochthonous populations such as that in the Luhya (LWK), Baganda, and Zulu, or BOT is very likely to reduce ROH in these groups. Individuals from CAB and MOZ from ethnolinguistics groups with n<2, mixed parental or grandparental languages, or >5% European ancestry are not shown.

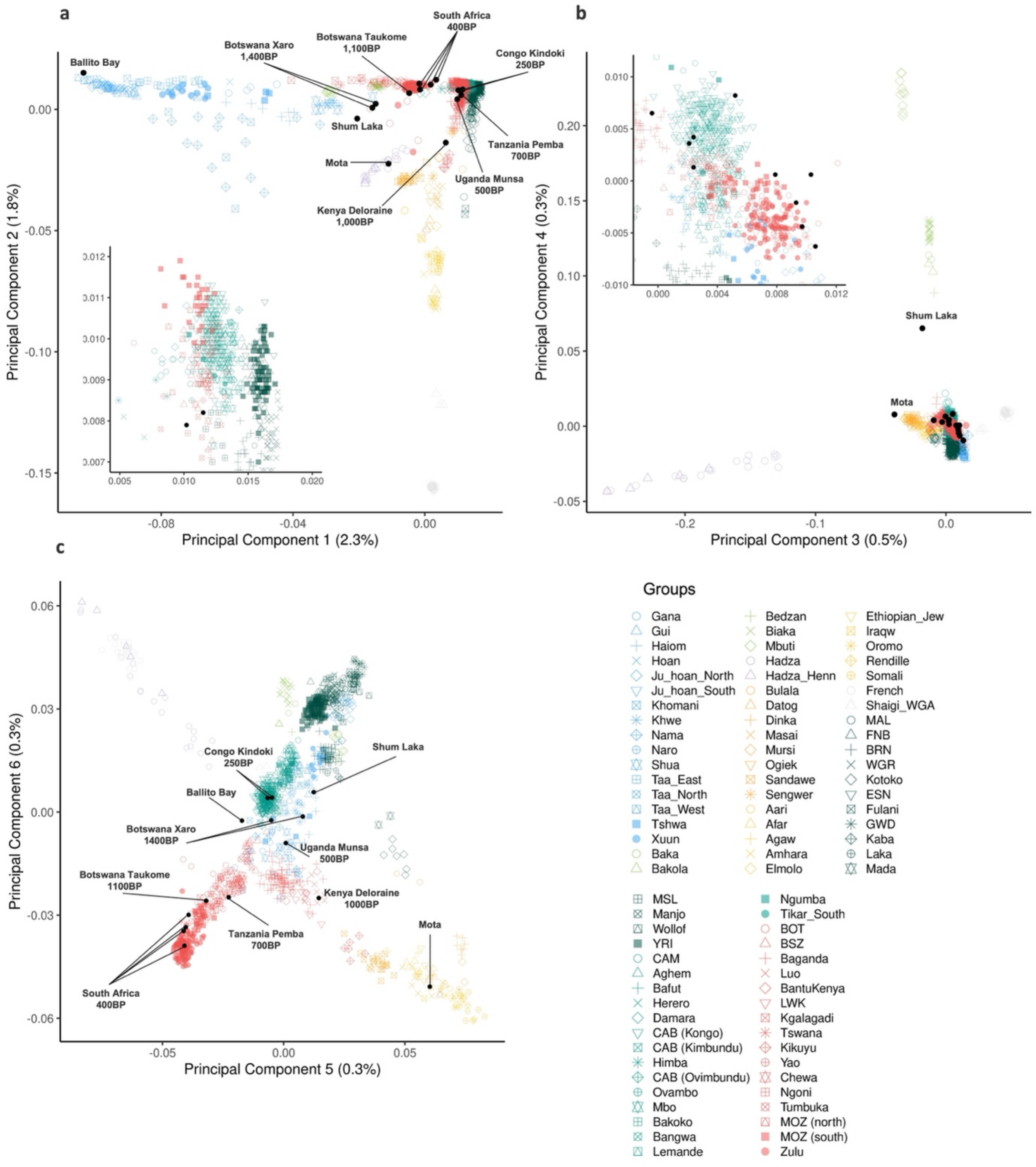

Supplementary Figure 6. (a) Principal Components Analysis (PCA) with eigenvectors (PC1 and PC2) constructed using modern groups (shown in legend) from a subset of the Human Origins Array (HOA) dataset enriched with African groups (Supplementary Table 2). Genomes introduced in this study are labelled with CAB and MOZ in the legend. Ancient African genomes (labelled on PCA axes in black) were projected onto these axes using least squares projection with correction

for PC shrinkage. Percentage of variation explained by each PC (eigenvalue K / sum of eigenvalues) are shown on each labelled axis. PCs zoomed on CAB and MOZ locations are shown to aid in visualisation. **(b)** As in (a) but showing PC3 and PC4. Note that only Shum Laka and Mota are labelled. **(c)** As in (a) but showing PC5 and PC6.

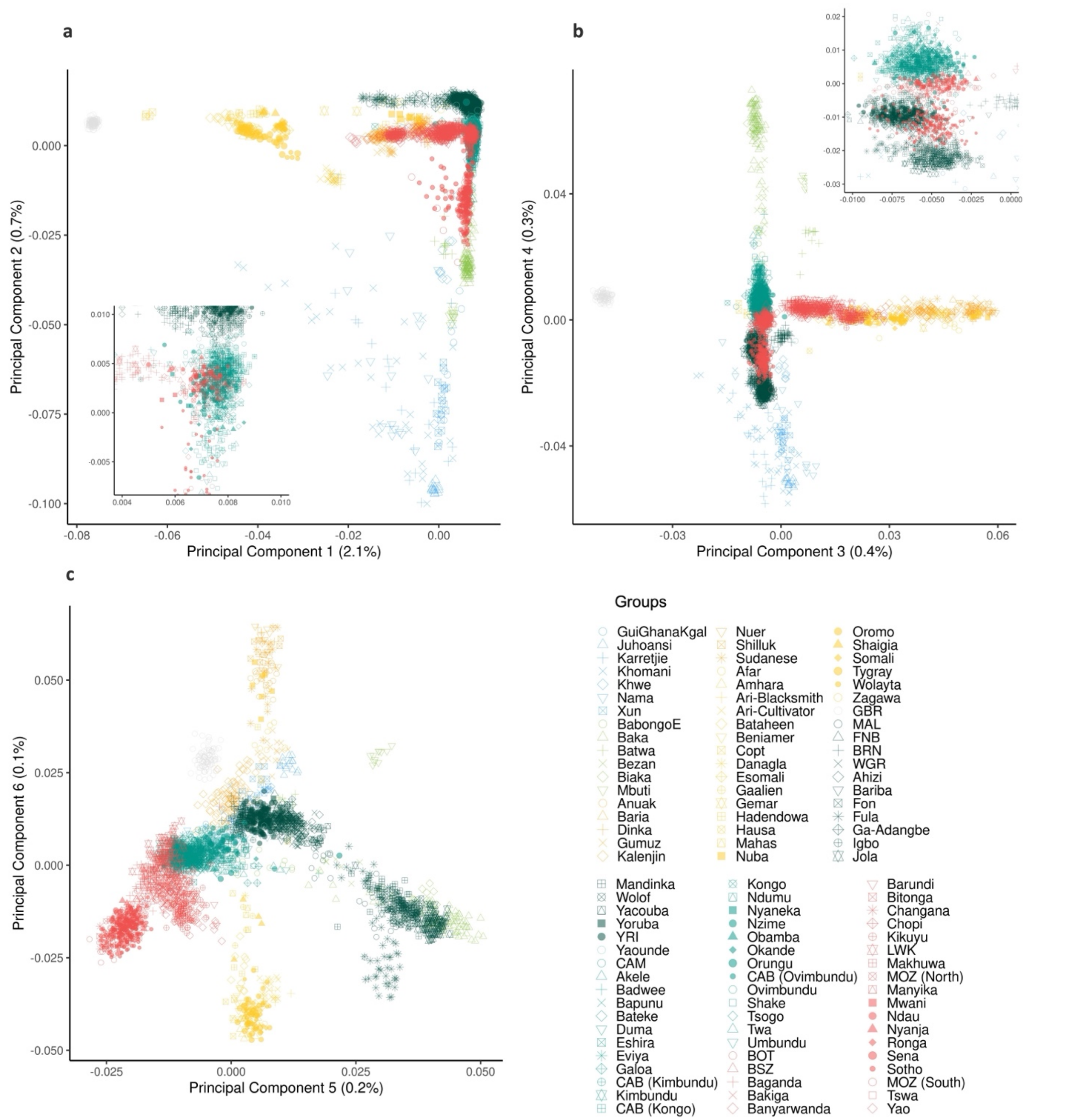

Supplementary Figure 7. **(a)** Principal Components Analysis (PCA) with eigenvectors (PC1 and PC2) constructed using groups (shown in legend) from the curated Illumina Array (Illumina) dataset (Supplementary Table 3). Genomes introduced in this study are labelled with CAB and MOZ in the legend. Percentage of variation explained by each PC (eigenvalue K / sum of eigenvalues) are shown on each labelled axis. PCs zoomed on CAB and MOZ locations are shown to aid in visualisation. **(b)** As in (a) but showing PC3 and PC4. (**c**) As in (a) but showing PC5 and PC6.

a

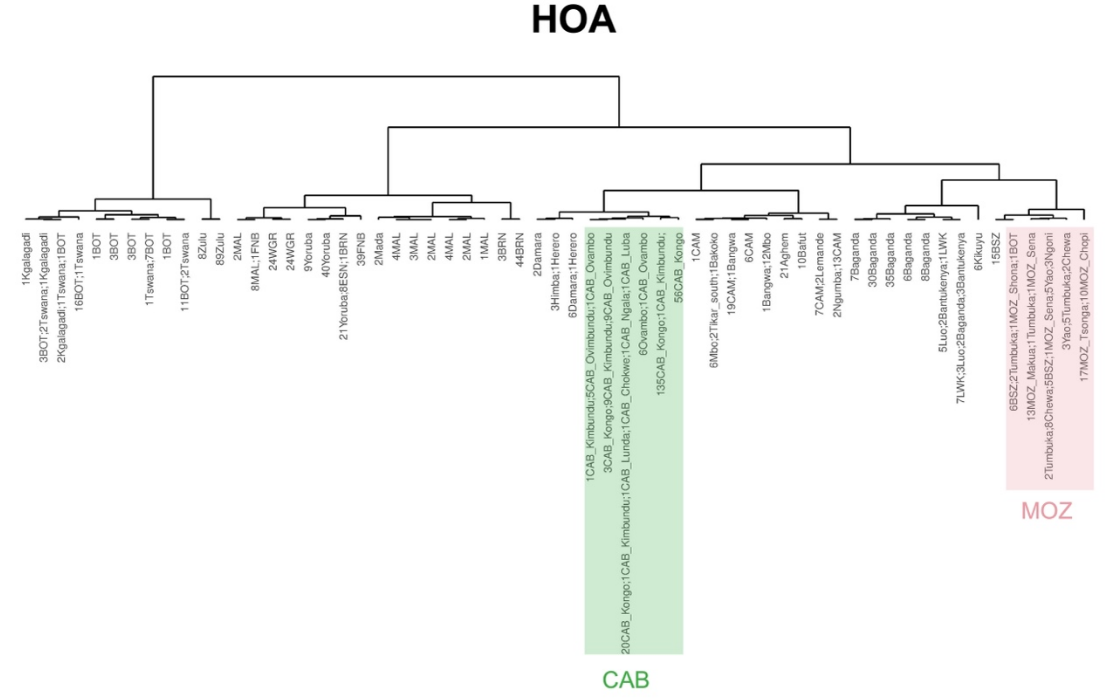

b

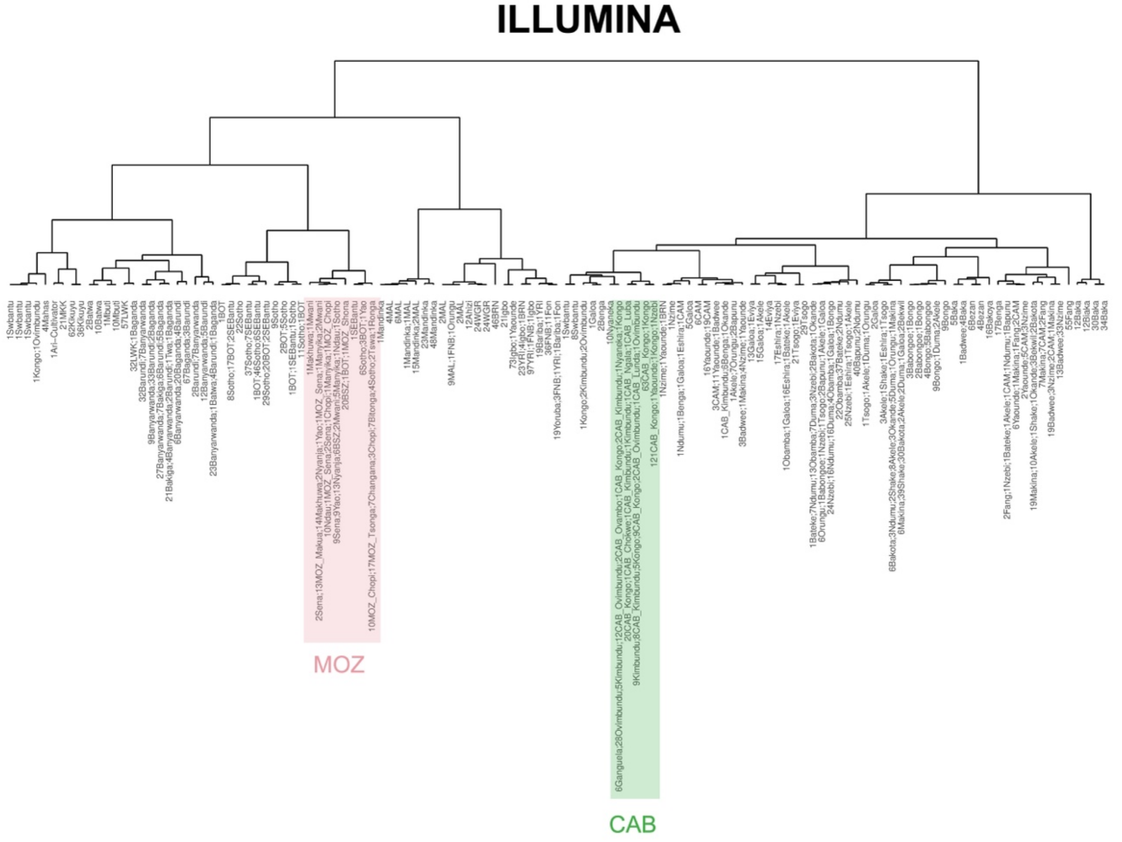

Supplementary Figure 8. Population structure of CAB and MOZ in the context of a subset of comparative groups present in the HOA (Supplementary Table 6) and ILLUMINA (Supplementary Table 7) datasets. **(a)** fineSTRUCTURE inferred dendrogram relating 61 clusters inferred using the chunk counts matrix generated by CHROMOPAINTER using the "*HOA all-Bantu-copying"* (see Methods 12). Note that this does not include ancient individuals due to the prerequisite of phased, diploid genotypes. **(b)** fineSTRUCTURE inferred dendrogram relating 126 clusters inferred using the chunk counts matrix generated CHROMOPAINTER using the "*ILLUMINA all-Bantu copying model*" (see Methods 12). We note that admixture with non-Bantu speaking sources (as shown in Supplementary Figures 8 & 9) is driving a large fraction of this observed population structure (e.g. Bantu-speakers from South Africa and Botswana appear early branching, however, this is likely a function of admixture with highly divergent Khoe/San groups). These trees should, therefore, not be taken as indicative of cladistic structure related to Bantu / Niger-Congo languages or separation of groups related to the Bantu expansion but rather as indicative of shared patterns of fine-scale population structure and local admixture among groups. Population labels beginning with CAB or MOZ e.g. CAB_Kongo and MOZ_Tsonga indicate newly sequenced individuals in this study.

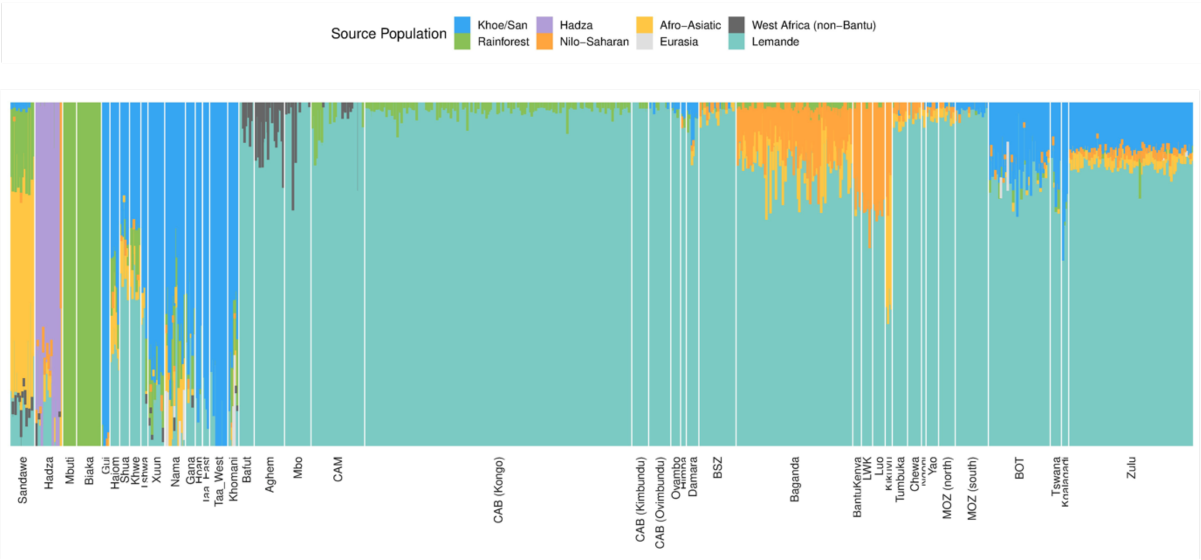

Supplementary Figure 9. SOURCEFIND inferred ancestry compositions of CAB and MOZ in a pan-African context. Performed using a matrix describing the total length of pairwise shared haplotype chunks generated using the HOA *"no-Bantu-copying model*". In this model, Bantu-speakers from across sub-Saharan Africa (see "Niger-Congo (B) - Bantu" in Supplementary Table 6) were excluded as possible donors to CHROMOPAINTER, other than the Lemande. This is to highlight the fraction of additional, autochthonous non-Bantu-related (Lemande) ancestry present in CAB and MOZ when compared to neighbouring groups that may have occurred e.g. through later interactions and admixture with local groups.

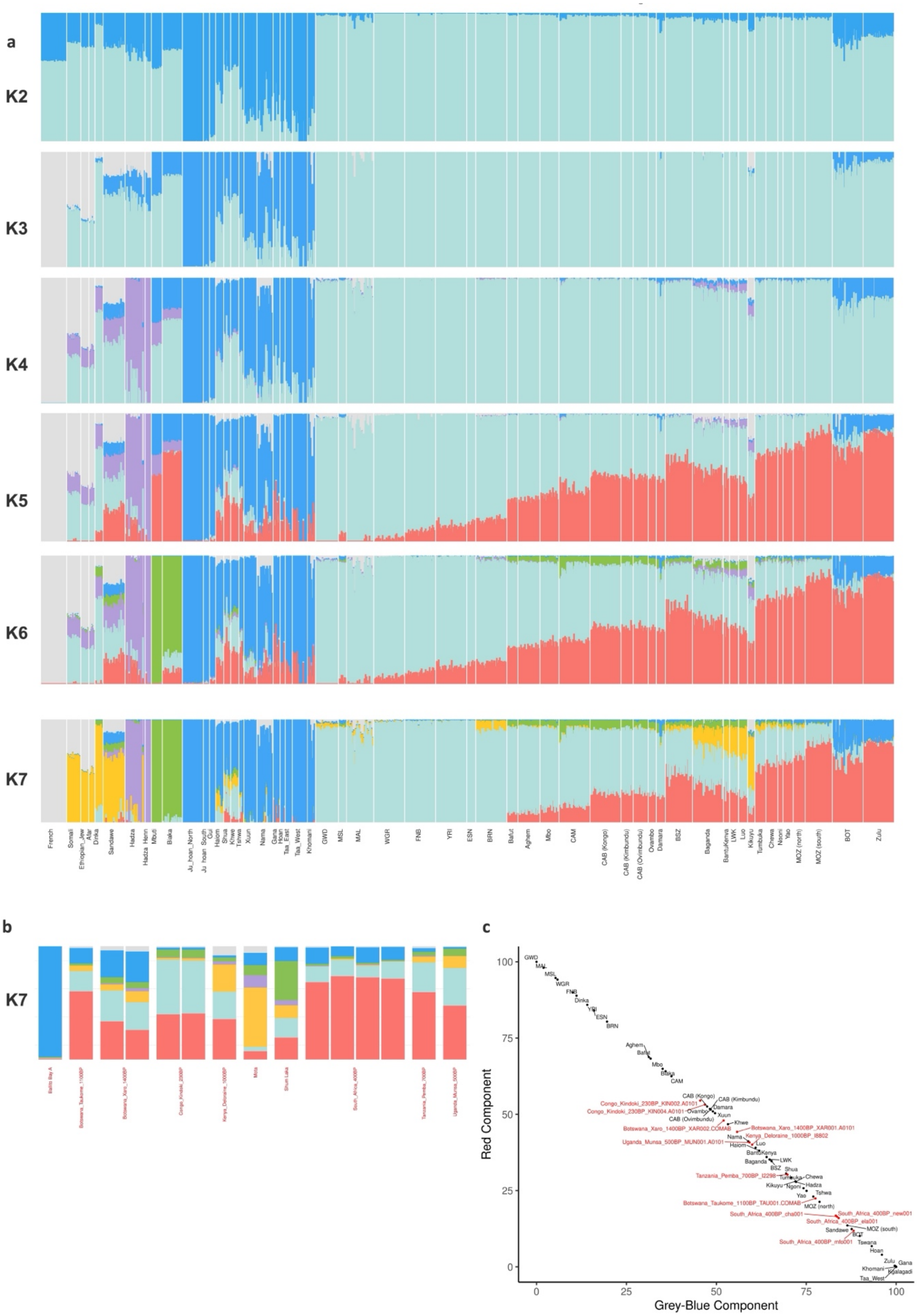
a
K2
K3
K4
K5
K6
K7
French
Somali
Ethiopian_Jew
Afar
Dinka
Sandawe
Hadza
Hadza_Henn
Mbuti
Biaka
Ju_hoan_North
Ju_hoan_South
Gui
Haiom
Shua
Khwe
Tshwa
Xuun
Nama
Gana
Hoan
Taa_East
Taa_West
Khomani
GWD
MSL
MAL
WGR
FNB
YRI
ESN
BRN
Bafut
Aghem
Mbo
CAM
CAB (Kongo)
CAB (Kimbundu)
CAB (Ovimbundu)
Ovambo
Damara
BSZ
Baganda
BantuKenya
LWK
Luo
Kikuyu
Tumbuka
Chewa
Ngoni
Yao
MOZ (north)
MOZ (south)
BOT
Zulu
b
K7
Ballito Bay A
Botswana_Taukome_1100BP
Botswana_Xaro_1400BP
Congo_Kindoki_230BP
Kenya_Deloraine_1000BP
Mota
Shum Laka
South_Africa_400BP
Tanzania_Pemba_700BP
Uganda_Munsa_500BP
c
Red Component
Grey-Blue Component
0
25
50
75
100
GWD
MAL
MSL
WGR
FNB
Dinka
YRI
ESN
BRN
Aghem
Bafut
Mbo
Biaka
CAM
CAB (Kongo)
CAB (Kimbundu)
Congo_Kindoki_230BP_KIN002.A0101
Damara
Congo_Kindoki_230BP_KIN004.A0101
Ovambo
Xuun
CAB (Ovimbundu)
Khwe
Botswana_Xaro_1400BP_XAR002.COMAB
Botswana_Xaro_1400BP_XAR001.A0101
Nama
Kenya_Deloraine_1000BP_I8802
Uganda_Munsa_500BP_MUN001.A0101
Luo
Haiom
BantuKenya
LWK
Baganda
BSZ
Shua
Tanzania_Pemba_700BP_I2298
Tumbuka
Chewa
Kikuyu
Ngoni
Hadza
Tshwa
Yao
Botswana_Taukome_1100BP_TAU001.COMAB
MOZ (north)
South_Africa_400BP_cha001
South_Africa_400BP_new001
South_Africa_400BP_ela001
Sandawe
MOZ (south)
BOT
South_Africa_400BP_mfo001
Tswana
Hoan
Zulu
Gana
Khomani
Kgalagadi
Taa_West

Supplementary Figure 10. Unsupervised ADMIXTURE clustering of Angolans and Mozambicans in a pan-African context. (**a**) Modern populations at *K*=2 to *K*=7. Groups were sub-sampled to a maximum of 30 individuals to aid in visualisation. *K=7* represents the best-guess at matching *K* to the number of true ancestral populations (lowest cross-validation error) (**b**) Ancient populations, including early hunter-gather populations from Cameroon (Shum Laka, 8,000 years old), Ethiopia (Mota, 4,000 years old) and South Africa (Ballito Bay, 2,000 years old) and Bantu-associated individuals whose DNA was extracted from remains present at sites from 1,400 to 240 years ago projected onto clusters generated by modern populations at *K*=7. (c) Normalised proportion (%) of grey-blue (West Africa related) vs red (Southern Bantu related) ancestry components at *K=7*. Ancient groups are highlighted in red. ADMIXTURE clusters can be taken as evidence of admixture components. However, such interpretations are likely to be affected by population-specific drift and unreasonable assumptions regarding the divergence history of populations across Africa. For example, the Mbuti here appear entirely unadmixed, but fastGLOBETROTTER results clearly shows recent admixture has occurred in the history of this group (Figure 2, Supplementary Table 8).

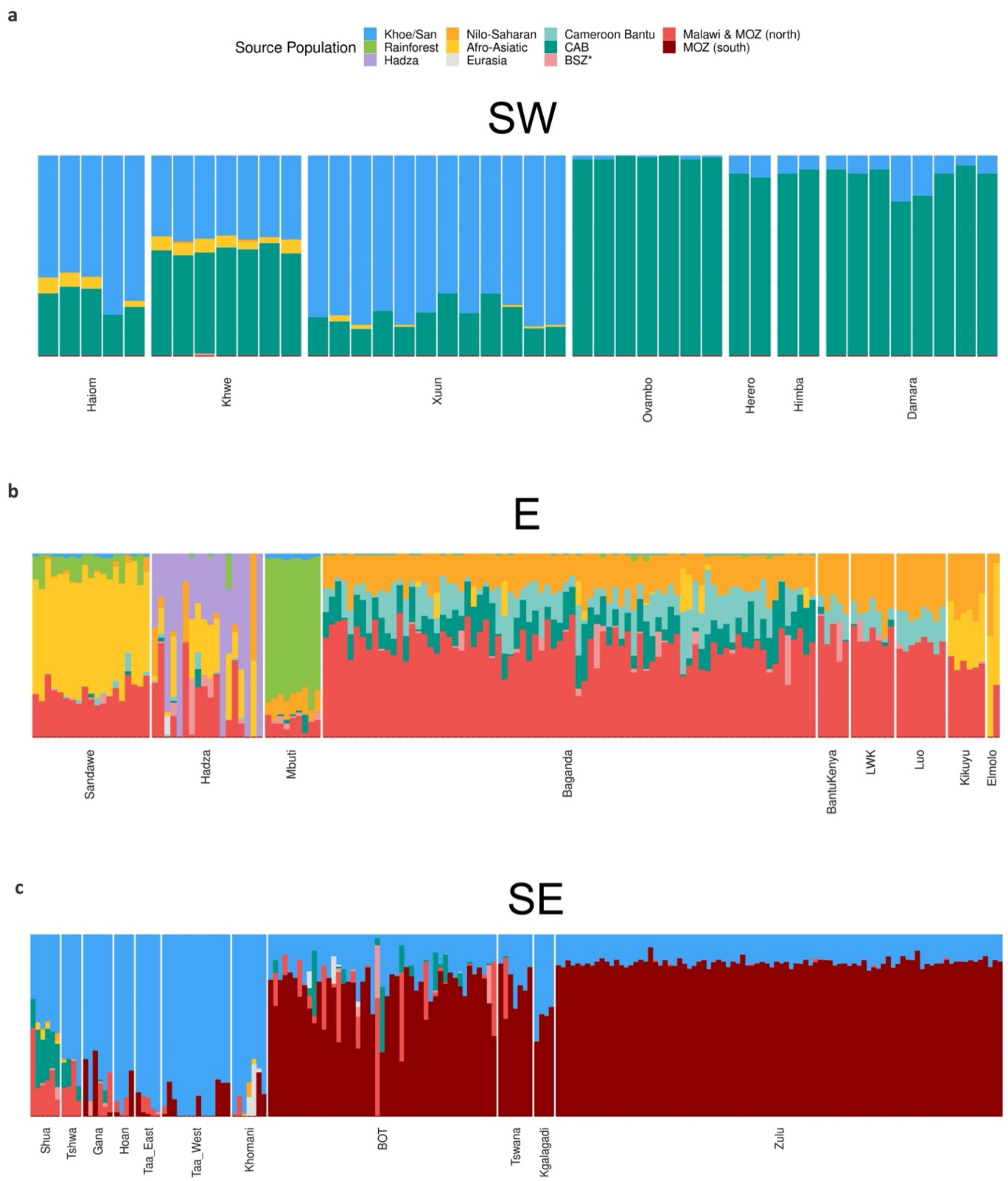

Supplementary Figure 11. Individual chromosome paintings inferred using SOURCEFIND in step 5 of our stepwise analysis. Colours are merged into the groups of individuals (regional Bantu speaking groups) included as sources. In addition to all other source groups present in the HOA dataset: Cameroon Bantu = step 1, CAB = step 2, BSZ (BSZ that cluster independently from Malawians and appear as an intermediate between Angolans and Malawians) = step 3, Malawian

Bantu speakers and MOZ (north) = step 4, MOZ (south) = step 5. See Supplementary Note 5 and Supplementary Table 8 for full details **(a)** South Western groups from Namibia. **(b)** Eastern groups from Tanzania, Kenya, and Uganda. **(c)** South Eastern or South Central groups from Botswana and South Africa. We note that BOT is an ethnolinguistically heterogeneous meta-population (see Choudhury et al.[18]), which is likely the cause of heterogeneity in ancestry matching in this step and makes interpretation challenging. However, within-group heterogeneity is also apparent among Khoe/San peoples from across Botswana – which may be evidence that these groups contain ancestries derived from both early and later migrations of Bantu speakers into Botswana and South Africa.

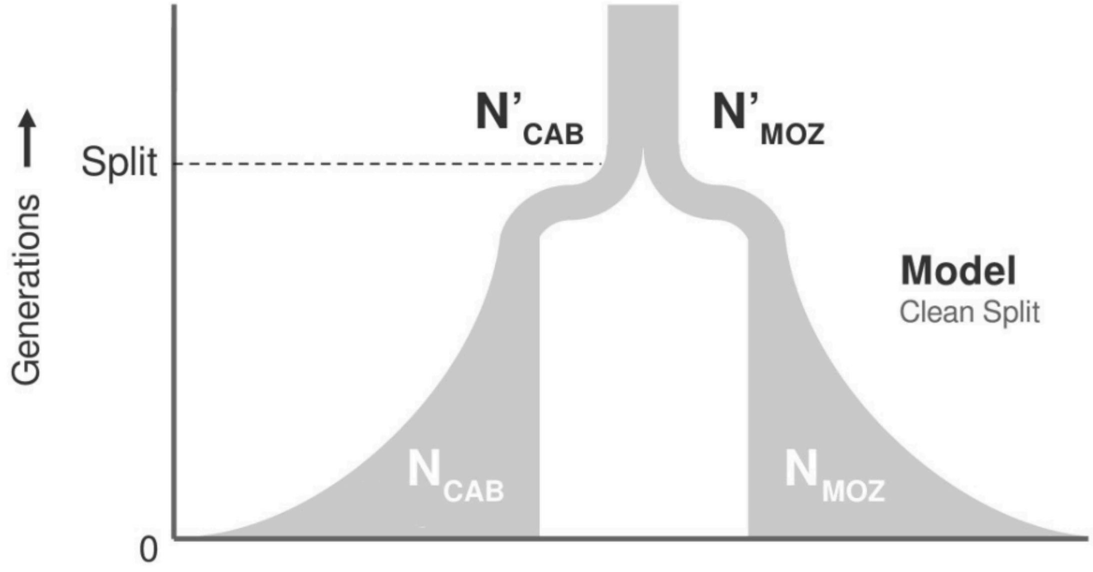

Supplementary Figure 12. Model of population separation between the ancestors of Bantu-speakers from CAB and MOZ. This Clean Split model includes as parameters: the divergence time generation split and the population sizes N'_{MOZ} and N'_{CAB} at time generation Split which grow/decay exponentially until generation 0 until size N_{MOZ} and N_{CAB} respectively as shown in grey.

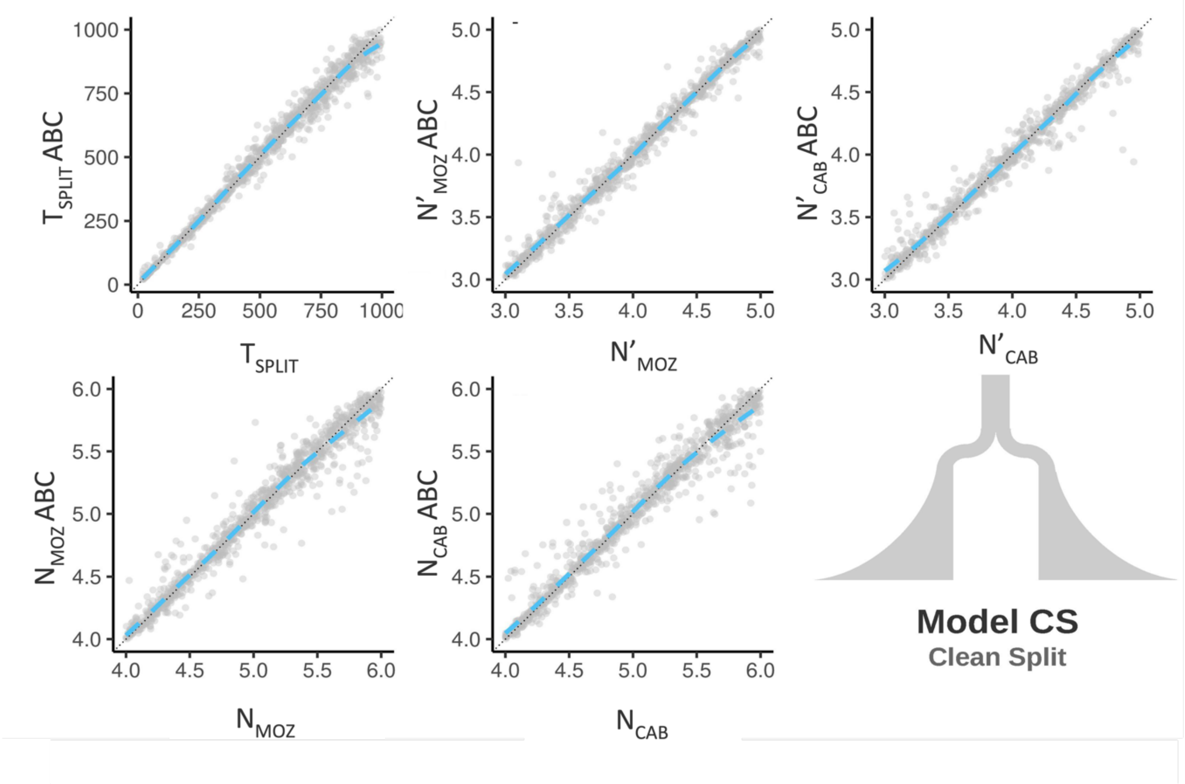

Supplementary Figure 13. Accuracy of *abc* ('neuralnet') at estimating the true parameter values using reference tables of summary statistics generated under the *clean split* demographic model (Supplementary Figure 10). For 1,000 pseudo-observed simulations from which the true values of the parameters were known, for each parameter value, *abc* was used to estimate the posterior distribution of the parameter, and the median of the distribution was taken as our estimate of the true parameter value. Blue line is the average of the median point estimates, dotted line is x=y.

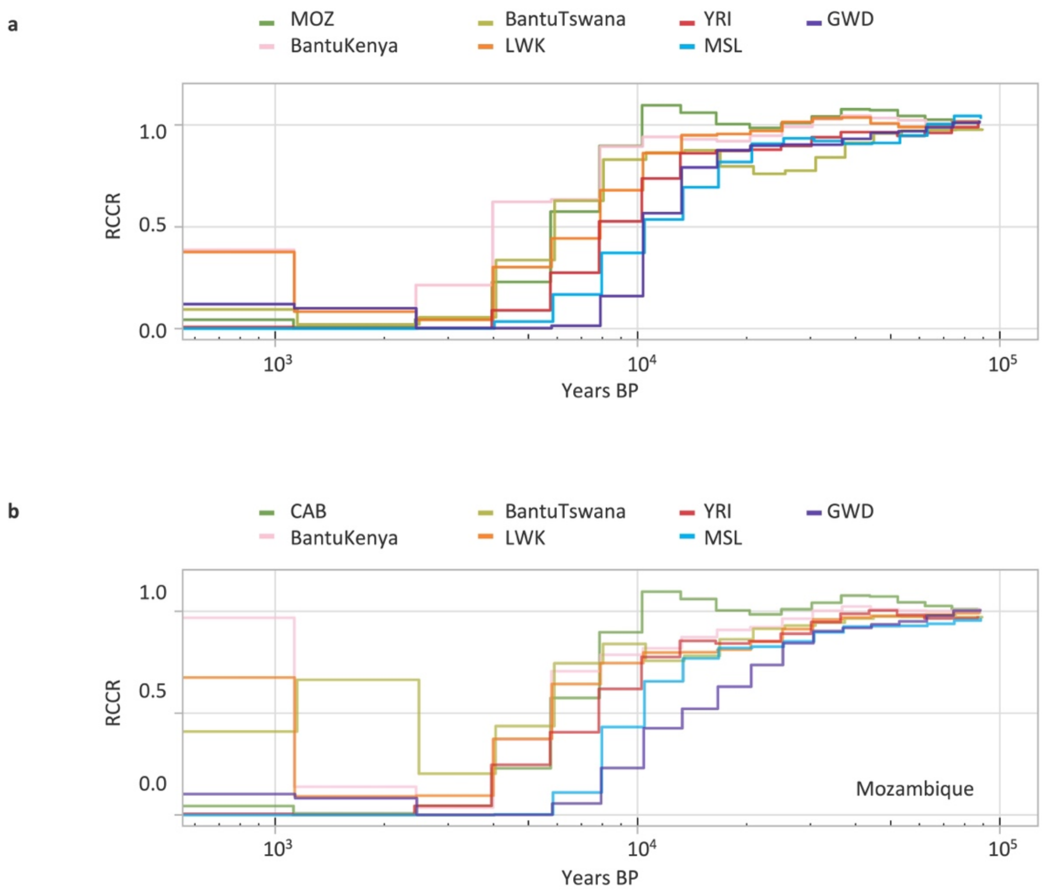

Supplementary Figure 14. (a) Separation history of CAB (1 Kongo, 1 Kimbundu) estimated MSMC2 with two-high coverage (37X) whole-genomes from CAB and two from either MOZ or Niger-Congo speaking populations from the Simons Genome Diversity Project (SGDP). Separation times were taken as the first generation going backwards-in-time in which RCCR is greater than or equal to 0.5 **(b)** As in a but for MAP (1 Tsonga, 1 Makua). See Supplementary Note 3.10 for details of these high-coverage (37X) genomes. LWK, Luhya from Kenya; YRI, Yoruba from Nigeria; MSL, Mende from Sierra Leone; GWD, Mandenka from The Gambia; BantuKenya, Bantu-speakers from Kenya (HGDP); BantuTswana, Tswana-speakers from South Africa.

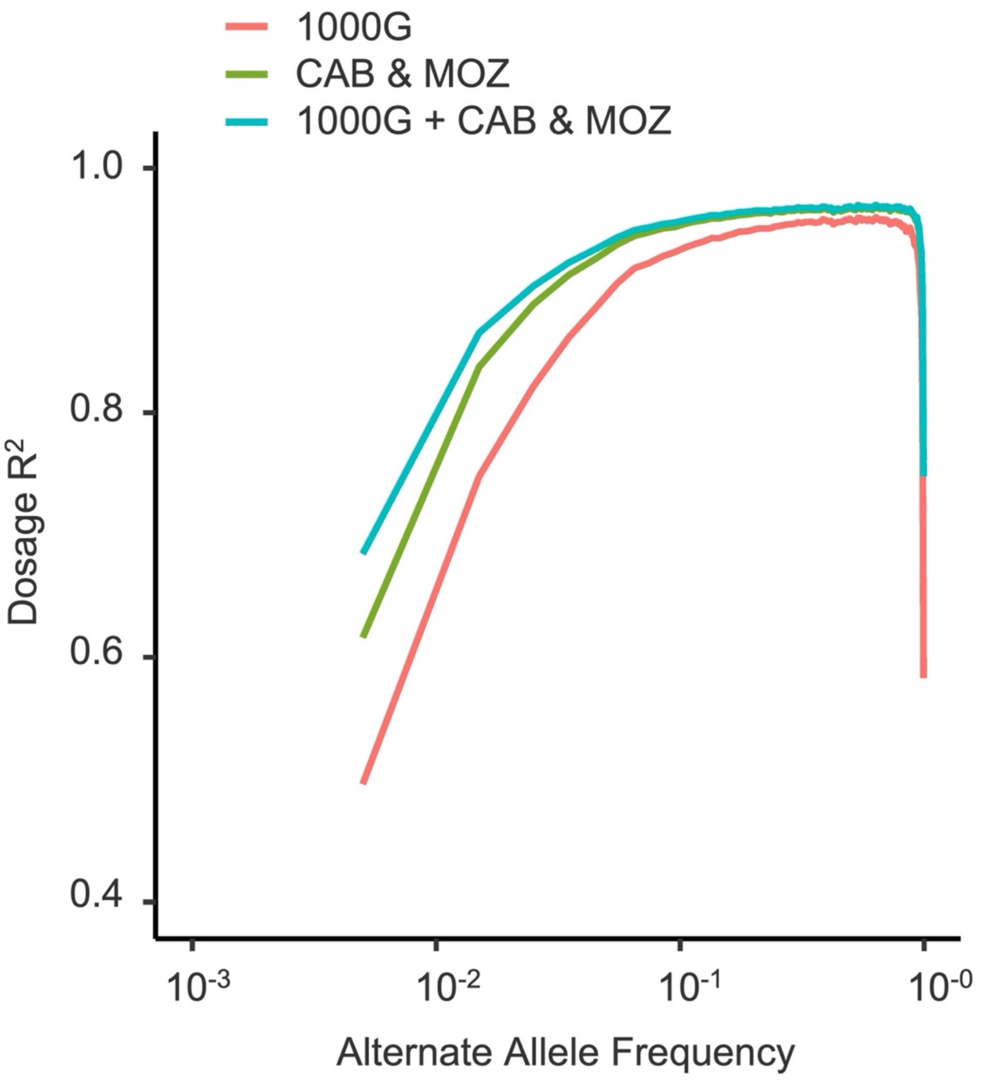

Supplementary Figure 15. Dosage R2 (Pearson's squared correlation coefficient) of called genotype vs genotypes imputed into 10 Mozambicans from MOZ and 50 Angolans from CAB using either the 1000G reference panel, the remaining 280 newly sequenced genomes from CAB and MOZ (unrelated to the 4th degree as estimated using KING), or a merged reference panel including the 1000G (full dataset, as compared to Figure 4 being subsampled) and newly sequenced genomes from CAB and MOZ as a function of alternate allele frequency. We only compared loci that appeared across all panels.

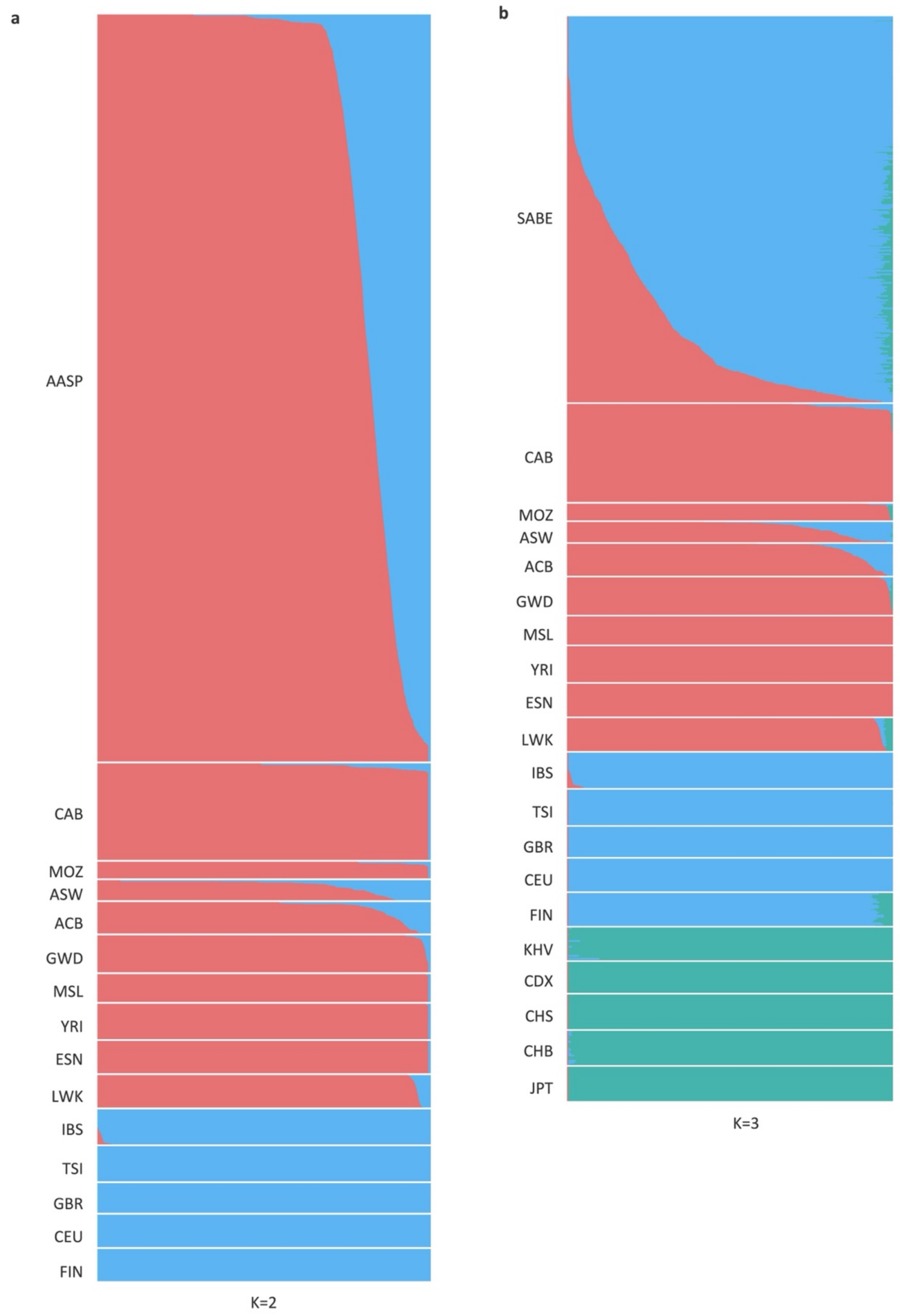

Supplementary Figure 16. (a) ADMIXTURE clustering at K=2 showing the proportion of West African-related (red) and European-related (blue) ancestry in individuals from the African

American Sequencing Project (AASP) alongside samples from CAB and MOZ, 1000G-AFR, and 1000G-EUR. **(b)** ADMIXTURE clustering at K=3 showing the proportion of West African-related (red), European-related (blue), and East Asian (also a proxy for Native Amerindian) (blue-green) ancestry in individuals from the Saúde Bem Estar e Envelhecimento project (SABE) project alongside samples from CAB and MOZ 1000G-AFR, 1000G-EUR, and 1000G-EAS. 1000G-EAS was not included as a reference when performing analysis on the AASP dataset, as there was no evidence of EAS-like ancestry at K=3.

a

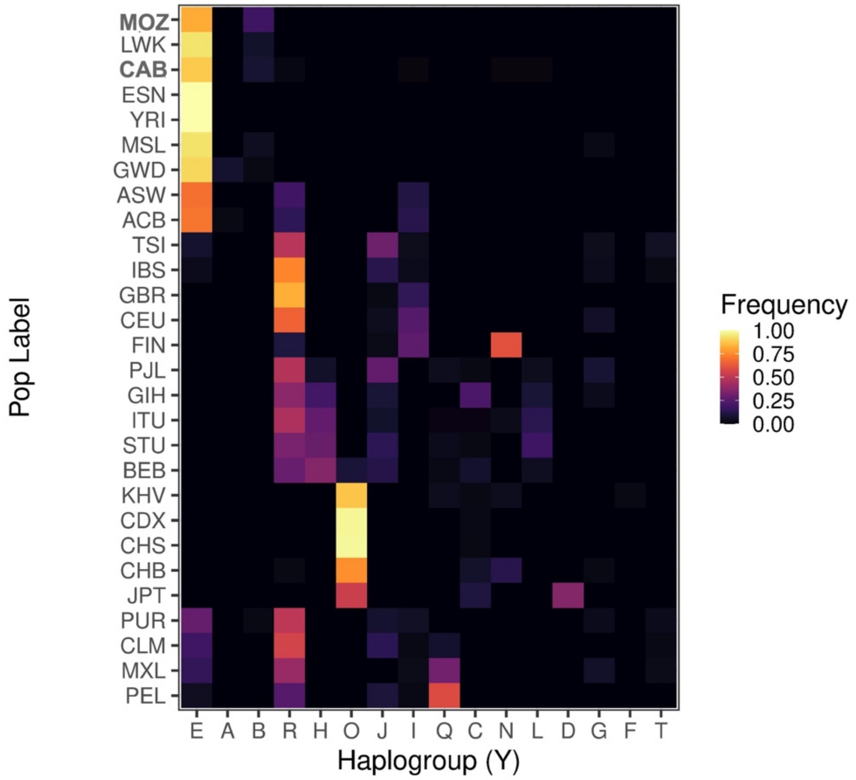
MOZ
LWK
CAB
ESN
YRI
MSL
GWD
ASW
ACB
TSI
IBS
GBR
CEU
FIN
PJL
GIH
ITU
STU
BEB
KHV
CDX
CHS
CHB
JPT
PUR
CLM
MXL
PEL
Pop Label
E A B R H O J I Q C N L D G F T
Haplogroup (Y)
Frequency
1.00
0.75
0.50
0.25
0.00

b

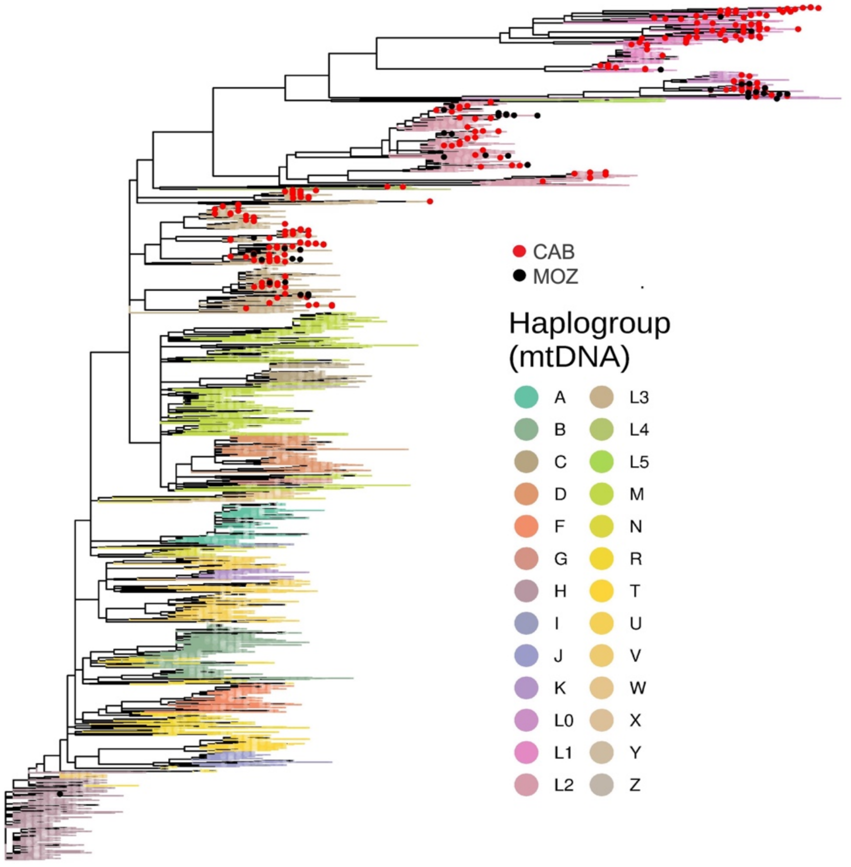
CAB
MOZ
Haplogroup (mtDNA)
A
B
C
D
F
G
H
I
J
K
L0
L1
L2
L3
L4
L5
M
N
R
T
U
V
W
X
Y
Z

Supplementary Figure 17. Uniparental markers in CAB and MOZ compared to global populations from the 1000G. **(a)** mtDNA tree relating genomes from CAB (red point) and MOZ (black point) alongside samples from the 1000G. **(b)** Major Y-chromosomal haplogroup frequency across CAB and MOZ compared to populations from the 1000G.

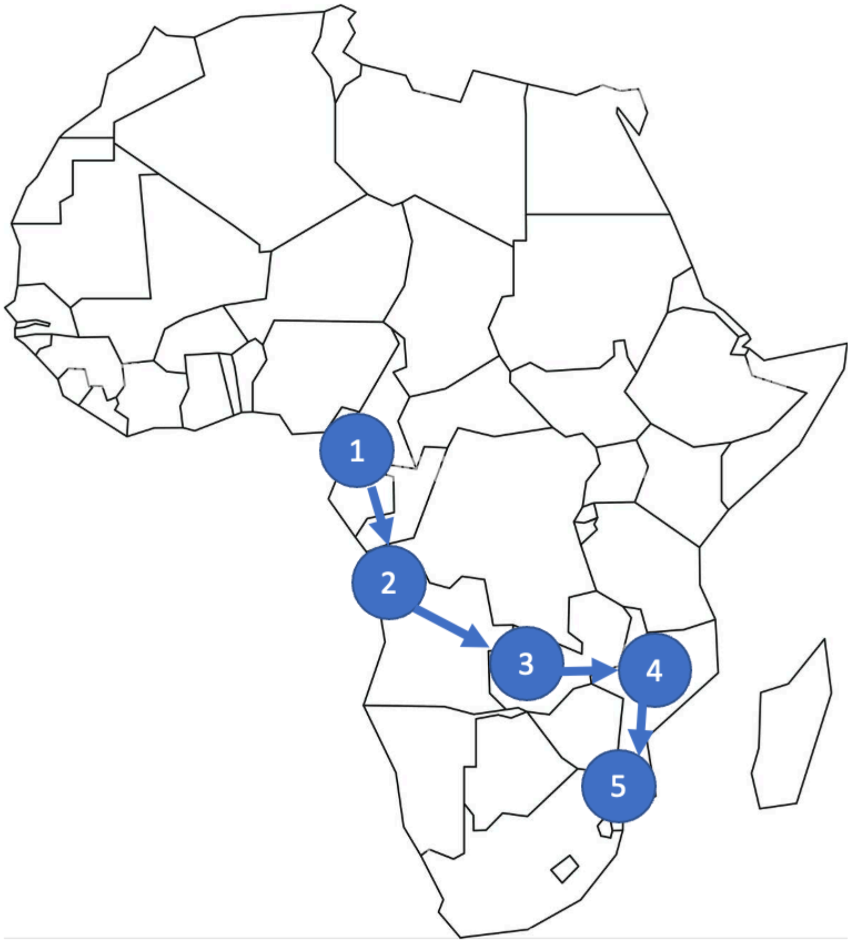

Supplementary Figure 18. Skeletal outline of the model of stepwise migration that the SOURCEFIND analysis described here is based on. Map made with Natural Earth. Free vector and raster map data @ naturalearthdata.com.

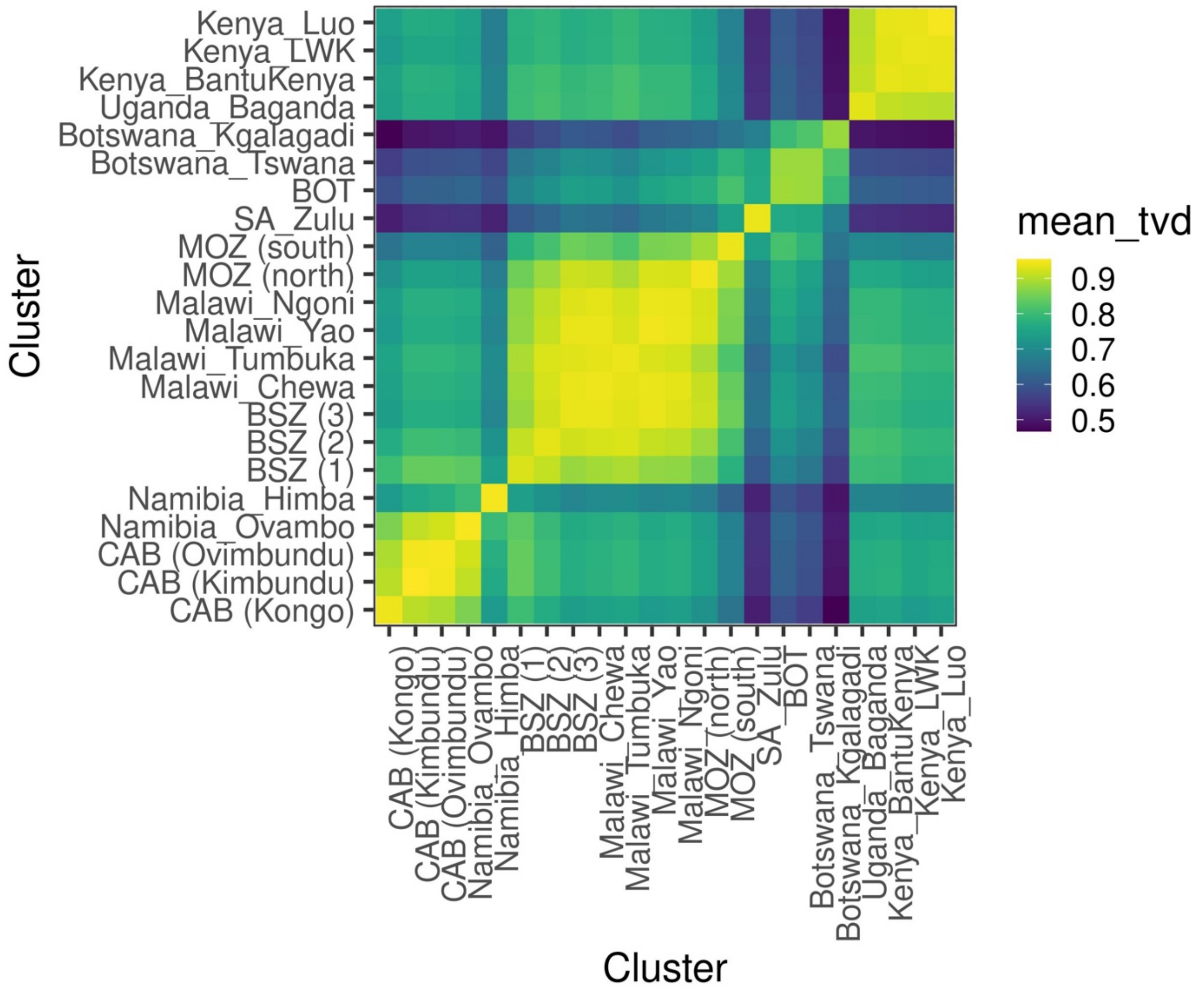

Supplementary Figure 19. TVD sharing matrix a subset of pairs of Bantu-speaking groups in the HOA dataset as calculated using the all-copying model. TVD was calculated with Supplementary Equation 1. BSZ (1) refers to BSZ labelled individuals that do not cluster with Malawians, whilst BSZ (2/3) refers to individuals that do cluster with Malawians according to fineSTRUCTURE.

URLs

One thousand genomes dataset (1000G): ftp://ftp.1000genomes.ebi.ac.uk/vol1/ftp/phase1/analysis_results/supporting/ancestral_alignments).
1000G genetic map: *https://github.com/joepickrell/1000-genomes-genetic-maps*
AADR: https://reich.hms.harvard.edu/allen-ancient-dna-resource-aadr-downloadable-genotypes-present-day-and-ancient-dna-data
Base R: https://www.r-project.org/
Beagle Genetic Map: http://bochet.gcc.biostat.washington.edu/beagle/genetic_maps/
boot.pval: https://cran.r-project.org/web/packages/boot.pval/index.html
Chromosome Painting: http://paintmychromosomes.com/
dbSNP150: ftp.ncbi.nih.gov/snp/organisms/human_9606/VCF/
ENCODE Blacklist: https://www.encodeproject.org/annotations/ENCSR636HFF/
Ethnologue : https://www.ethnologue.com/
GATK Best Practices Pipeline: https://www-ncbi-nlm-nih-gov.ezproxy3.lib.le.ac.uk/refseq/
hg19/GRCh37: https://www.ncbi.nlm.nih.gov/assembly/GCF_000001405.13/
hg38/GRCh38: https://www.ncbi.nlm.nih.gov/assembly/GCF_000001405.26/
ISOGG: https://isogg.org/tree/ISOGG_YDNA_SNP_Index.html
Low Complexity Regions Mask: https://github.com/lh3/varcmp/raw/master/scripts/LCR-hs37d5.bed.gz
NGX Bio: https://ngxbio.com/
Novogene: https://en.novogene.com/
panTro5: https://www-ncbi-nlm-nih-gov.ezproxy3.lib.le.ac.uk/assembly/GCF_000001515.7/
PLINK V2.0: https://www.cog-genomics.org/plink/2.0/
rCRS: https://www.mitomap.org/MITOMAP/HumanMitoSeq
RefSeq: https://www-ncbi-nlm-nih-gov.ezproxy3.lib.le.ac.uk/refseq/
SGDP: https://reichdata.hms.harvard.edu/pub/datasets/sgdp/
SOURCEFIND manual: https://github.com/hellenthal-group-UCL/sourcefindV2
UCSC Segmental Duplications: https://humanparalogy.gs.washington.edu/build37/build37.htm

Supplementary References

1. Li H. Minimap2: pairwise alignment for nucleotide sequences. *Bioinformatics* **34**, 3094-3100 (2018).

2. Li H*, et al*. The Sequence Alignment/Map format and SAMtools. *Bioinformatics* **25**, 2078-2079 (2009).

3. McKenna A*, et al*. The Genome Analysis Toolkit: A MapReduce framework for analyzing next-generation DNA sequencing data. *Genome Research* **20**, 1297-1303 (2010).

4. Bonfield JK, McCarthy SA, Durbin R. Crumble: reference free lossy compression of sequence quality values. *Bioinformatics* **35**, 337-339 (2019).

5. Pedersen BS, Quinlan AR. Mosdepth: quick coverage calculation for genomes and exomes. *Bioinformatics* **34**, 867-868 (2018).

6. Zhang F*, et al*. Ancestry-agnostic estimation of DNA sample contamination from sequence reads. *Genome Research* **30**, 185-194 (2020).

7. Altshuler DM*, et al*. A global reference for human genetic variation. *Nature* **526**, 68-74 (2015).

8. Breitwieser FP, Baker DN, Salzberg SL. KrakenUniq: confident and fast metagenomics classification using unique k-mer counts. *Genome Biology* **19**, 198 (2018).

9. Sherman RM*, et al*. Assembly of a pan-genome from deep sequencing of 910 humans of African descent. *Nat Genet* **51**, 30-35 (2019).

10. Samson CA, Whitford W, Snell RG, Jacobsen JC, Lehnert K. Contaminating DNA in human saliva alters the detection of variants from whole genome sequencing. *Sci Rep-Uk* **10**, 19255 (2020).

11. Kanderi T, Shrimanker I, Mansoora Q, Shah K, Yumen A, Komanduri S. Stenotrophomonas maltophilia: An Emerging Pathogen of the Respiratory Tract. *Am J Case Rep* **21**, e921466-921461–e921466-921464 (2020).

12. Rodriguez-Medina N, Barrios-Camacho H, Duran-Bedolla J, Garza-Ramos U. Klebsiella variicola: an emerging pathogen in humans. *Emerg Microbes Infec* **8**, 973-988 (2019).

13. Tom JA*, et al*. Identifying and mitigating batch effects in whole genome sequencing data. *Bmc Bioinformatics* **18**, 351 (2017).

14. Browning SR, Browning BL. Rapid and accurate haplotype phasing and missing-data inference for whole-genome association studies by use of localized haplotype clustering. *Am J Hum Genet* **81**, 1084-1097 (2007).

15. Fang H*, et al*. Reducing INDEL calling errors in whole genome and exome sequencing data. *Genome Med* **6**, 89 (2014).

16. Danecek P, *et al*. The variant call format and VCFtools. *Bioinformatics* **27**, 2156-2158 (2011).

17. Li H. A statistical framework for SNP calling, mutation discovery, association mapping and population genetical parameter estimation from sequencing data. *Bioinformatics* **27**, 2987-2993 (2011).

18. Choudhury A, *et al*. High-depth African genomes inform human migration and health. *Nature* **586**, 741-748 (2020).

19. Poznik GD. Identifying Y-chromosome haplogroups in arbitrarily large samples of sequenced or genotyped men. *bioRxiv*, 088716 (2016).

20. Berniell-Lee G, *et al*. Genetic and demographic implications of the Bantu expansion: insights from human paternal lineages. *Mol Biol Evol* **26**, 1581-1589 (2009).

21. Brito P, *et al*. Y-SNP Analysis in an Angola Population. *Forensic Science International: Genetics Supplement Series* **3**, 2 (2011).

22. Li MK, Schroder R, Ni SY, Madea B, Stoneking M. Extensive tissue-related and allele-related mtDNA heteroplasmy suggests positive selection for somatic mutations. *P Natl Acad Sci USA* **112**, 2491-2496 (2015).

23. Katoh K, Misawa K, Kuma K, Miyata T. MAFFT: a novel method for rapid multiple sequence alignment based on fast Fourier transform. *Nucleic Acids Res* **30**, 3059-3066 (2002).

24. Weissensteiner H, *et al*. HaploGrep 2: mitochondrial haplogroup classification in the era of high-throughput sequencing. *Nucleic Acids Res* **44**, W58-W63 (2016).

25. van Oven M. PhyloTree Build 17: Growing the human mitochondrial DNA tree. *Forens Sci Int-Gen S* **5**, E392-E394 (2015).

26. Price MN, Dehal PS, Arkin AP. FastTree 2-Approximately Maximum-Likelihood Trees for Large Alignments. *Plos One* **5**, 0009490 (2010).

27. Quintana-Murci L, *et al*. Maternal traces of deep common ancestry and asymmetric gene flow between Pygmy hunter-gatherers and Bantu-speaking farmers. *P Natl Acad Sci USA* **105**, 1596-1601 (2008).

28. Castri L, *et al*. mtDNA Variability in Two Bantu-Speaking Populations (Shona and Hutu) From Eastern Africa: Implications for Peopling and Migration Patterns in Sub-Saharan Africa. *Am J Phys Anthropol* **140**, 302-311 (2009).

29. Pakendorf B, Stoneking M. Mitochondrial DNA and human evolution. *Annu Rev Genom Hum G* **6**, 165-183 (2005).

30. Marks SJ, *et al*. Static and Moving Frontiers: The Genetic Landscape of Southern African Bantu-Speaking Populations. *Molecular Biology and Evolution* **32**, 29-43 (2015).

31. de Filippo C, Bostoen K, Stoneking M, Pakendorf B. Bringing together linguistic and genetic evidence to test the Bantu expansion. *Proc Biol Sci* **279**, 3256-3263 (2012).

32. Manichaikul A, Mychaleckyj JC, Rich SS, Daly K, Sale M, Chen WM. Robust relationship inference in genome-wide association studies. *Bioinformatics* **26**, 2867-2873 (2010).

33. Purcell S*, et al*. PLINK: A tool set for whole-genome association and population-based linkage analyses. *American Journal of Human Genetics* **81**, 559-575 (2007).

34. Schiffels S, Durbin R. Inferring human population size and separation history from multiple genome sequences. *Nat Genet* **46**, 919-925 (2014).

35. Mallick S*, et al*. The Simons Genome Diversity Project: 300 genomes from 142 diverse populations. *Nature* **538**, 201-206 (2016).

36. Li H, Durbin R. Fast and accurate short read alignment with Burrows-Wheeler transform. *Bioinformatics* **25**, 1754-1760 (2009).

37. Bostoen K. Kikongo dialect continuum: internal and external classification. (2012).

38. Schlebusch CM*, et al*. Southern African ancient genomes estimate modern human divergence to 350,000 to 260,000 years ago. *Science* **358**, 652-655 (2017).

39. Llorente MG*, et al*. Ancient Ethiopian genome reveals extensive Eurasian admixture throughout the African continent. *Science* **350**, 820-822 (2015).

40. Lipson M*, et al*. Ancient West African foragers in the context of African population history. *Nature* **577**, 665-670 (2020).

41. Asuni N, Wilder S. VariantKey: A Reversible Numerical Representation of Human Genetic Variants. *bioRxiv*, 473744 (2019).

42. Quinlan AR, Hall IM. BEDTools: a flexible suite of utilities for comparing genomic features. *Bioinformatics* **26**, 841-842 (2010).

43. Gurdasani D*, et al*. The African Genome Variation Project shapes medical genetics in Africa. *Nature* **517**, 327-332 (2015).

44. Alexander DH, Novembre J, Lange K. Fast model-based estimation of ancestry in unrelated individuals. *Genome Res* **19**, 1655-1664 (2009).

45. Patin E*, et al*. Dispersals and genetic adaptation of Bantu-speaking populations in Africa and North America. *Science* **356**, 543-546 (2017).

46. Wang K*, et al*. Ancient genomes reveal complex patterns of population movement, interaction, and replacement in sub-Saharan Africa. *Sci Adv* **6**, eaaz0183 (2020).

47. Skoglund P*, et al*. Reconstructing Prehistoric African Population Structure. *Cell* **171**, 59-71 (2017).

48. Prendergast ME*, et al*. Ancient DNA reveals a multistep spread of the first herders into sub-Saharan Africa. *Science* **365**, eaaw6275 (2019).

49. Fan SH*, et al*. African evolutionary history inferred from whole genome sequence data of 44 indigenous African populations *Genome Biology* **20**, 82 (2019).

50. Busby GBJ*, et al*. Admixture into and within sub-Saharan Africa. *Elife* **5**, e15266 (2016).

51. Semo A, *et al*. Along the Indian Ocean Coast: Genomic Variation in Mozambique Provides New Insights into the Bantu Expansion. *Molecular Biology and Evolution* **37**, 406-416 (2020).

52. Sengupta D, *et al*. Genetic substructure and complex demographic history of South African Bantu speakers. *Nat Commun* **12**, (2021).

53. Chacon-Duque JC, *et al*. Latin Americans show wide-spread Converso ancestry and imprint of local Native ancestry on physical appearance. *Nat Commun* **9**, 5388 (2018).

54. Hellenthal G, *et al*. A genetic atlas of human admixture history. *Science* **343**, 747-751 (2014).

55. Lawson DJ, Hellenthal G, Myers S, Falush D. Inference of Population Structure using Dense Haplotype Data. *Plos Genet* **8**, e1002453 (2012).

56. Lopez S, *et al*. Evidence of the interplay of genetics and culture in Ethiopia. *Nat Commun* **12**, 3581 (2021).

57. Gusev A, *et al*. Whole population, genome-wide mapping of hidden relatedness. *Genome Research* **19**, 318-326 (2009).
