# Supplementary material for: Whole genomes from Angola and Mozambique inform about the origins and dispersals of major African migrations
Source: Nat Commun. 2023 Dec 2;14:7967. doi: 10.1038/s41467-023-43717-x (PMC10693643; doi:10.1038/s41467-023-43717-x)
Supplement: Supplementary file 2 — Description of Additional Supplementary Files [file 41467_2023_43717_MOESM2_ESM.pdf]

Description of Additional Supplementary Files

File Name: Supplementary Data 1
Description: Per sample information of the data presented in Table 1 in the main text as well as quality control metrics calculated as described in Supplementary Note.

File Name: Supplementary Data 2
Description: Table containing WGS populations from the 1000G. AGVP, H3AB, SGDP, and high-coverage ancient African genomes datasets merged with newly sequenced Angolans and Mozambicans for population structure in a pan Africa context in the main text or utilised to identify EU ancestry among CAB and MOZ (see Supplementary Data 1, EUROPEAN ancestry ADMIXTURE (%))

File Name: Supplementary Data 3
Description: Table containing modern and ancient populations genotyped at sites present on the Human Origins Array (HOA) dataset. SNPs in newly sequenced (This study) CAB and MOZ were recalled specifically at HOA sites as described in Supplementary Note. We excluded individuals with significant proportions (>5%) of European ancestry among CAB and MAP (see Supplementary Data 1) as estimated by ADMIXTURE, such admixture is likely to have occurred within the last 4-5 generations and can mask signals related to more ancient mixture/haplotype sharing. We also exclude individuals who are unrelated to the <4th degree as estimated by KING across the merged HOA dataset.

File Name: Supplementary Data 4
Description: Table containing modern groups genotyped across various Illumina SNP Arrays panels from many previous studies (ILLUMINA) and combined across an intersection of sites with <5% missingness across the sample set. This dataset was also filtered for individuals <4th degree relatives using KING.

File Name: Supplementary Data 5
Description: Table containing the results of our stepwise SOURCEFIND and fastGLOBETROTTER analysis described in Supplementary Note 5 and summarised in Figure 2 of the main text. Below we detail the Population IDs of Bantu/Bantoid speaking groups (Niger-Congo (B)) that were included (from our HOA dataset, see Supplementary Data 6) as source populations in each step, in addition to all other groups as potential surrogates. FastGLOBETROTTER was only run on those groups where Cameroon/Bantoid is replaced in step 1, using the source groups described in the most recent step in which they were analysed. We only retain those runs with an r2 best fit of >0.5 (strong evidence of admixture). Those below 0.5 are highlighted in red.

STEP 1: Surrogates include all non-Bantu/Bantoid speaking groups in HOA dataset + CAM (separated into 4 fineSTRUCTURE clusters labelled 1, 2, 3, 4), Lemande, Tikar South, Ngumba, Mbo, Aghem, Bafut, Bakoko, Bangwa, Mada, Baka, Bakola, Bedzan.
STEP 2: CAB (Kongo), CAB (Ovimbundu), CAB (Kimbundu) added as surrogates.
STEP 3: BSZ* added as surrogates. BSZ* includes only individuals in BSZ that clustered entirely independently from Malawians, and appeared as an intermediate between CAB and Malawians/MOZ as well as those individuals in BSZ that cluster with Malawians/MOZ (see Supplementary Note 5.3).
STEP 3 (alt): Baganda added as surrogates. (Retained CAB ancestries in Malawians, MOZ, BSZ may suggest the Baganda does not well model an intermediate between CAB and these groups).
STEP 4: Tumbuka, Chewa, Ngoni, Yao, MOZ (north) added as surrogates. MOZ (north) contains Makua peoples among MOZ.

STEP 4 (alt): Kgalagadi, Tswana, BOT added as surrogates. (Retained BSZ* ancestries in MOZ & Malawians may suggest Botswanans do not well model an intermediate between BSZ* and these groups).
STEP 5: MOZ (south) added as surrogates, which includes Chopi and Tsonga peoples among MOZ.
We note that fractions of ancestry should not be interpreted as admixture proportions, but rather as an attempt by SOURCEFIND to best model haplotype sharing apparent across the dataset using the surrogates provided (e.g. MOZ (south) shows 18% ancestry matched to Ballito Bay A in step 1, but this is reduced to <1% when MOZ (north) is added as a surrogate). See Supplementary Figure 6 for a more robust analysis of admixture fractions.
