# Supplementary material for: Whole genomes from Angola and Mozambique inform about the origins and dispersals of major African migrations
Source: Nat Commun. 2023 Dec 2;14:7967. doi: 10.1038/s41467-023-43717-x (PMC10693643; doi:10.1038/s41467-023-43717-x)
Supplement: Supplementary file 4 — Reporting Summary [file 41467_2023_43717_MOESM4_ESM.pdf]

nature portfolio

Corresponding author(s): Sandra Beleza

Last updated by author(s): Jul 19, 2023

Reporting Summary

Nature Portfolio wishes to improve the reproducibility of the work that we publish. This form provides structure for consistency and transparency in reporting. For further information on Nature Portfolio policies, see our Editorial Policies and the Editorial Policy Checklist.

Statistics

For all statistical analyses, confirm that the following items are present in the figure legend, table legend, main text, or Methods section.

n/a	Confirmed	
☐	☒	The exact sample size (*n*) for each experimental group/condition, given as a discrete number and unit of measurement
☐	☒	A statement on whether measurements were taken from distinct samples or whether the same sample was measured repeatedly
☐	☒	The statistical test(s) used AND whether they are one- or two-sided<br>*Only common tests should be described solely by name; describe more complex techniques in the Methods section.*
☐	☒	A description of all covariates tested
☐	☒	A description of any assumptions or corrections, such as tests of normality and adjustment for multiple comparisons
☐	☒	A full description of the statistical parameters including central tendency (e.g. means) or other basic estimates (e.g. regression coefficient) AND variation (e.g. standard deviation) or associated estimates of uncertainty (e.g. confidence intervals)
☐	☒	For null hypothesis testing, the test statistic (e.g. *F*, *t*, *r*) with confidence intervals, effect sizes, degrees of freedom and *P* value noted<br>*Give P values as exact values whenever suitable.*
☐	☒	For Bayesian analysis, information on the choice of priors and Markov chain Monte Carlo settings
☐	☒	For hierarchical and complex designs, identification of the appropriate level for tests and full reporting of outcomes
☐	☒	Estimates of effect sizes (e.g. Cohen's *d*, Pearson's *r*), indicating how they were calculated

Our web collection on statistics for biologists contains articles on many of the points above.

Software and code

Policy information about availability of computer code

Data collection	No software was used in data collection
Data analysis	abc R-package (https://cran.r-project.org/web/packages/abc/vignettes/abcvignette.pdf)<br>ADMIXTOOLSv7.0.2(https://github.com/DReichLab/AdmixTools)(contains R package admixr)<br>ADMIXTURE v1.22 (https://dalexander.github.io/admixture/index.html)<br>ANNOVAR (https://annovar.openbioinformatics.org/en/latest/user-guide/download/)<br>Beagle v4.1 (https://faculty.washington.edu/browning/beagle/b4_1.html)<br>CHROMOPAINTERv2 (https://github.com/sahwa/ChromoPainterv2)<br>EIGENSOFT v7.2.1 (https://www.hsph.harvard.edu/alkes-price/software/)<br>fastGLOBETROTTER (https://github.com/sahwa/fastGLOBETROTTER)<br>fineSTRUCTURE v2.1.3 (https://people.maths.bris.ac.uk/—madjl/finestructure/finestructure.html)<br>GATK v4.0.2.1 (https://gatk.broadinstitute.org/hc/en-us)<br>genotyping error-rates simulation custom R script (https://github.com/spTallman/vcfErr()<br>geosphere R package (https://github.com/rspatial/geosphere)<br>GERMLINE (https://github.com/gusevlab/germline (for ABC simulation; supl. Table 5))<br>IBDseq version r1206 (https://faculty.washington.edu/browning/ibdseq.html)<br>IMPUTE2 (https://mathgen.stats.ox.ac.uk/impute/impute_v2.html)<br>KrakenUniq (https://github.com/fbreitwieser/krakenuniq)<br>Metrics R-package (https://github.com/mfrasco/Metrics)<br>minimap2 v2.11-r797 (https://github.com/lh3/minimap2)<br>mosdepth v0.2.3, (https://github.com/brentp/mosdepth)

MSMC2(https://github.com/stschiff/msmc)
msprime release 1.2(https://tskit.dev/msprime)
PLINK v1.9(https://www.cog-genomics.org/plink/)
PLINK v2.0 (https://www.cog-genomics.org/plink/2.0/)
qpAdm, R package admixr (https://github.com/bodkan/admixr)
Relate v1.1 (https://myersgroup.github.io/relate/).
samtools v1.9 (http://www.htslib.org/)
Scikit allel python package (https://scikit-allel.readthedocs.io/en/stable/)(for ABC simulation; Supl. Table 5)
seqkt (https://github.com/lh3/seqtk; https://docs.csc.fi/apps/seqtk/)
SHAPEITv2 (https://mathgen.stats.ox.ac.uk/genetics_software/shapeit/shapeit.html)
Shared f2 calculation custom R script (https://github.com/spTallman/f2)
SOURCEFINDv2 (https://github.com/sahwa/sourcefindV2)
VCFTOOLS v1.014 (https://vcftools.sourceforge.net/)
VerifyBamID2(https://github.com/Griffan/VerifyBamID)
To draw the map figures, rnaturalearth R package (https://cran.r-project.org/web/packages/rnaturalearth/index.html)

For manuscripts utilizing custom algorithms or software that are central to the research but not yet described in published literature, software must be made available to editors and reviewers. We strongly encourage code deposition in a community repository (e.g. GitHub). See the Nature Portfolio guidelines for submitting code & software for further information.

Data

Policy information about availability of data

All manuscripts must include a data availability statement. This statement should provide the following information, where applicable:

- Accession codes, unique identifiers, or web links for publicly available datasets
- A description of any restrictions on data availability
- For clinical datasets or third party data, please ensure that the statement adheres to our policy

Individual-level sequence datasets (compressed BAM files or CRAM files) and variant calling datasets (VCF files) generated in this study have been deposited at the European Genome-phenome Archive (EGA) under under EGA Study accession number EGAS00001007458 (EGA Data accession number and hyperlink to make available before paper publication) Corresponding data on ethnolinguistic group is reported on Supplementary Data 1. This data is for research use only. The African Genome Variation Project (AGVP) genomic data is available in EGA under accession code EGAD00001001663 (https://ega-archive.org/datasets/EGAD00001001663). H3Africa-Baylor (H3A) data was obtained is available in EGA under accession codes: EGAD00001004220 (https://ega-archive.org/datasets/EGAD00001004220); EGAD00001004316 (https://ega-archive.org/datasets/EGAD00001004316; EGAD00001004393 (https://ega-archive.org/datasets/EGAD00001004393); EGAD00001004533 (https://ega-archive.org/datasets/EGAD00001004533); EGAD00001004505 (https://ega-archive.org/datasets/EGAD00001004505); EGAD00001004334 (https://ega-archive.org/datasets/EGAD00001004334); EGAD00001004557 (https://ega-archive.org/datasets/EGAD00001004557); and EGAD00001004448 (https://ega-archive.org/datasets/EGAD00001004448). Three high coverage African Ancient Genomes were obtained from https://www.ebi.ac.uk/ena/browser/view/PRJNA295861; https://www.ebi.ac.uk/ena/browser/view/PRJEB22660; https://reich.hms.harvard.edu/datasets. The SGDP was obtained from https://reichdata.hms.harvard.edu/pub/datasets/sgdp/. The HOA was obtained from https://reich.hms.harvard.edu/datasets; https://ega-archive.org/datasets/EGAD00010002100; https://www.ebi.ac.uk/ena/browser/view/PRJEB36063. The ILLUMINA dataset was obtained from https://ega-archive.org/datasets/EGAD00010000965; https://ega-archive.org/datasets/EGAD00010000496; https://www.ebi.ac.uk/biostudies/arrayexpress/studies/E-MTAB-8450; https://datadryad.org/stash/dataset/doi:10.5061/dryad.bs06h; http://sbimb.core.wits.ac.za/data/SNPgenotyping_01.html; http://mega.bioanth.cam.ac.uk/data/Ethiopia; https://ega-archive.org/datasets/EGAD00010000616; https://www.ebi.ac.uk/biostudies/arrayexpress/studies/E-MTAB-1259; https://github.com/bmhenn/khoesan_arraydata. AASP genomic dataset was obtained from dbGAP: dataset no. phs001102.v1.p1, and SABE genomic dataset was kindly provided by the author but can now be obtained from EGA under accession number EGAD00001008640 (https://ega-archive.org/datasets/EGAD00001008640).

Research involving human participants, their data, or biological material

Policy information about studies with human participants or human data. See also policy information about sex, gender (identity/presentation), and sexual orientation and race, ethnicity and racism.

Reporting on sex and gender	The study's main goal is to evaluate how major recent migrations and subsequent population history (e.g. admixture) have influenced the distribution of genomic (autosomal) variation across Sub-Saharan African populations. The evolutionary history of females and male lineages of these populations is not the main focus of the paper. Therefore, our analyses were not conditioned on sex. Because of this we do not to report the sex of the individuals.
Reporting on race, ethnicity, or other socially relevant groupings	The participants provided their, their parents', and their grandparents' ethnolinguistic affiliation. Individuals who reported speaking the same language as their parents and grandparents were classified into major linguistic groups using the Ethnologue database (www.ethnologue.com). Other geographic partitions of the data were also considered such as collection site and place-of-birth. These partitions (language and/or geography) were used in both supervised analyses, where individuals are grouped according to those partitions and the genomic variation is compared among groups (e.g. via calculation of f2 alleles, IBD, FST, ROH), and in unsupervised analyses, where the partitions are only considered a posteriori, to evaluate if the distribution of genomic data obtained in the analyses is associated with any of the partitions of the data (e.g. PCA, Chromopainter/Finestructure, ADMIXTURE analyses) .
Population characteristics	Participants were of both sexes (53% males) and ages ranging between 18 -73 years old, original from Angola and Mozambique and living in Cabinda and Maputo cities. Self-reported ethnolinguistic affiliation, sex and age were collected for each individual, their parents and grandparents. Individuals were classified into 15 language groups/Population labels using the Ethnologue database (www.ethnologue.com) (supplementary Data 1). Whole genomes sequences were obtained from these individuals and their variation analysed as described above. The study focuses in the characterization of the population groups. No other phenotypic information is included.

Recruitment	Participants were informed volunteers over the age of 18, with DNA samples and demographic characteristics collected via visits to Cabinda and Mozambique. To obtain a random sampling of the population, sample collection was done in several public places in Cabinda and Maputo cities, after public talks about the study subject. Public places varied from popular market places to local Universities.
Ethics oversight	This study was approved by the ethics committees of the University 11th of November ("Universidade 11 de Novembro"), Cabinda, Angola (REf: UoN/2016), Pedagogic University ("Universidade Pedagegica"), Maputo, Mozambique (REf: UP/2017), and the Medicine and Biological Sciences ethics committee at UoL (REf: 11334-sdsbl-genetics). All study participants provided their informed consent.

Note that full information on the approval of the study protocol must also be provided in the manuscript.

Field-specific reporting

Please select the one below that is the best fit for your research. If you are not sure, read the appropriate sections before making your selection.

☒ Life sciences ☐ Behavioural & social sciences ☐ Ecological, evolutionary & environmental sciences

For a reference copy of the document with all sections, see nature.com/documents/nr-reporting-summary-flat.pdf

Life sciences study design

All studies must disclose on these points even when the disclosure is negative.

Sample size	All samples for which we generated sequencing data were used. Sample sizes for these new samples are within the range of sample sizes of comparison datasets collected. According to Hellenthal et al. 2014. Science 343.747, these are sufficient for the population structure analyses performed. Random sampling to same size samples was performed for population history simulations and calculation of population genetics parameters such as population effective sizes, split times, and gene flow rates, needed to calculate comparable summary statistics from the data.
Data exclusions	Following data collection, we excluded related individuals based on kinship coefficients estimated using KING v2.2.5, and individuals with evidence of recent European ancestry estimated using ADMIXTURE v1.22
Replication	Using whole genome sequencing data and haplotype-based inference tools we were able to replicate (and extend) conclusions from previous studies, namely the timing and origin of the Bantu dispersals towards East Africa (the late split hypothesis), and the timing and intensity of (more recent) admixture with local populations at the edge of the Bantu expansion (in Southwest and Southeast Africa).
Randomization	There were no experimental groups. Individuals were selected and labeled based on having concordant ethnolinguistic affiliation reported for their parents and grandparents (found in table 1 and supplementary Data 1).
Blinding	Blinding does not apply to our study design, given that our study looks into how ethnolinguistic labels associate with genetics. We used genetics-based cluster algorithms that avoid the use of preconceived ethnolinguistic labels to define genetic sampling units, which clustered individuals largely based on their ethnolinguistic affiliation.

Reporting for specific materials, systems and methods

We require information from authors about some types of materials, experimental systems and methods used in many studies. Here, indicate whether each material, system or method listed is relevant to your study. If you are not sure if a list item applies to your research, read the appropriate section before selecting a response.

Materials & experimental systems

n/a	Involved in the study	
☒	☐	Antibodies
☒	☐	Eukaryotic cell lines
☒	☐	Palaeontology and archaeology
☒	☐	Animals and other organisms
☒	☐	Clinical data
☒	☐	Dual use research of concern
☒	☐	Plants

Methods

n/a	Involved in the study	
☒	☐	ChIP-seq
☒	☐	Flow cytometry
☒	☐	MRI-based neuroimaging
